# Exosome-transmitted circVMP1 facilitates the progression and cisplatin resistance of non-small cell lung cancer by targeting miR-524-5p-METTL3/SOX2 axis

**DOI:** 10.1080/10717544.2022.2057617

**Published:** 2022-04-25

**Authors:** Hongya Xie, Jie Yao, Yuxuan Wang, Bin Ni

**Affiliations:** a Department of Thoracic Surgery, The First Affiliated Hospital of Soochow University, Suzhou, China; b Department of Thoracic Surgery, The Affiliated Suzhou Hospital of Nanjing Medical University, Suzhou, China

**Keywords:** circVMP1, cisplatin, miR-524-5p, METTL3, SOX2, exosome

## Abstract

**Background:**

Circular RNAs (circRNAs) play important regulatory roles in multiple human malignancies, including non-small cell lung cancer (NSCLC). Here, we explored the role of circRNA vacuole membrane protein 1 (circVMP1) in NSCLC progression and cisplatin (DDP) resistance.

**Methods:**

The DDP resistance, proliferation, sphere formation ability, migration, invasion, and apoptosis of NSCLC cells were analyzed by Cell Counting Kit-8 (CCK8) assay, 5-ethynyl-2′-deoxyuridine (EdU) assay, sphere formation assay, wound healing assay, Transwell assay, and flow cytometry. Methylated RIP-qPCR (MeRIP-qPCR) was conducted to analyze the m^6^A modification level of SRY-box transcription factor 2 (SOX2). Dual-luciferase reporter assay, RNA immunoprecipitation (RIP) assay, and RNA-pull down assay were performed to confirm the intermolecular interaction. Exosomes were identified by transmission electron microscopy (TEM) and characterized by nanoparticle tracking analysis (NTA).

**Results:**

CircVMP1 expression was markedly elevated in DDP-resistant NSCLC cell lines compared with their parental cell lines. CircVMP1 absence restrained the proliferation, sphere formation, migration, invasion, and DDP resistance and promoted the apoptosis of DDP-resistant NSCLC cells. CircVMP1 acted as microRNA-524-5p (miR-524-5p) sponge to up-regulate the expression of methyltransferase 3, N6-adenosine-methyltransferase complex catalytic subunit (METTL3) and SOX2. CircVMP1 silencing restrained the malignant behaviors and DDP resistance of A549/DDP and H1299/DDP cells by targeting miR-524-5p. Exosomal circVMP1 disseminated the malignant properties and DDP resistance to DDP-sensitive cells. Exosomal circVMP1 elevated the DDP resistance of xenograft tumors *in vivo*. Exosomal circVMP1 was up-regulated in the serum samples of DDP-resistant NSCLC patients compared with DDP-sensitive patients.

**Conclusion:**

Exosome-mediated transmission of circVMP1 promoted NSCLC progression and DDP resistance by targeting miR-524-5p-METTL3/SOX2 axis.

**Highlights**
- CircVMP1 level is up-regulated in DDP-resistant NSCLC cell lines compared with DDP-sensitive cell lines.
- CircVMP1 absence restrains the malignant behaviors and DDP resistance of A549/DDP and H1299/DDP cells.
- CircVMP1-miR-524-5p/METTL3/SOX2 axis is identified for the first time.
- CircVMP1 plays an oncogenic role by targeting miR-524-5p-METTL3/SOX2 axis in A549/DDP and H1299/DDP cells.
- Exosomal circVMP1 transmits the malignant properties and DDP resistance to DDP-sensitive cells.

## Introduction

Non-small cell lung cancer (NSCLC) constitutes 85% of all lung cancer cases (Sosa Iglesias et al., 2018). Although the clinical outcomes of NSCLC patients have markedly improved, the development of chemo-resistance and metastasis are still two major obstacles for NSCLC treatment (Herbst et al., 2018; Duma et al., 2019). Hence, exploring the molecular mechanism behind the drug resistance and identifying novel effective bio-markers are important for NSCLC therapy.

Exosomes are small extracellular nano-vesicles and are implicated in intercellular communication through the delivery of vesicle contents, including RNAs and proteins (Théry et al., 2002). Cells-derived exosomes can be widely detected in blood, urine, saliva, and ascites (Simpson et al., 2009). Accumulating evidence have demonstrated that exosomes play important roles in multiple biological processes, including tumorigenesis (Zhang & Yu, 2019). For instance, exosomal circular RNA_0014235 (circ_0014235) is reported to elevate the DDP resistance and promote the development of NSCLC by mediating microRNA-520a-5p (miR-520a-5p)/CDK4 signaling (Xu et al., 2020).

Previous studies have indicated that circRNAs are implicated in tumor progression and affect cell proliferation, metastasis, and apoptosis (Patop & Kadener, 2018; Shang et al., 2019; Wang et al., 2020). CircRNAs are stable in exosomes, and serum exosomal circRNAs may be novel indicators for cancer diagnosis (Li et al., 2015). A previous study revealed that serum exosomal circRNA vacuole membrane protein 1 (circVMP1) is notably up-regulated in patients with lung adenocarcinoma compared with healthy controls (Lin et al., 2020). However, the biological significance of up-regulated circVMP1 in serum exosomes of lung cancer patients remains largely unclear.

CircRNAs have microRNA (miRNA) response elements (MREs), which can serve as miRNA sponges to suppress miRNA activity (Dori & Bicciato, 2019). Meanwhile, miRNAs can directly bind to the 3′ untranslated region (3′UTR) of messenger RNAs (mRNAs) to suppress the translation or induce the degradation of mRNAs (Fabian et al., 2010). Up to now, numerous articles have described the working mechanism of circRNAs in many biological processes through miRNAs-mRNAs signaling (Bi et al., 2018; Chen et al., 2018; Lu et al., 2020). Through bioinformatics prediction and functional experiments, circVMP1-miR-524-5p-methyltransferase 3, N6-adenosine-methyltransferase complex catalytic subunit (METTL3)/SRY-box transcription factor 2 (SOX2) axis was established, and their functional correlation was explored in this study.

In this study, we first assessed the roles of circVMP1 in the proliferation, stemness, migration, invasion, apoptosis, and chemo-resistance of NSCLC cells. CircVMP1-miR-524-5p-METTL3/SOX2 axis was established to illustrate the working mechanism of circVMP1 in NSCLC cells. Additionally, the role of exosomal circVMP1 in NSCLC progression was explored, and we also analyzed the diagnostic value of serum exosomal circVMP1 in the DDP resistance of NSCLC.

## Materials and methods

### Cell lines

Two NSCLC cell lines, including A549 (catalog number: BNCC337696) and H1299 (catalog number: BNCC100859), and 293 T (catalog number: BNCC341976) were purchased from BeNa culture collection (Beijing, China). DDP was purchased from Sigma (St. Louis, MO, USA). A549/DDP and H1299/DDP cell lines were established based on the protocol of a previous article (Jiang et al., 2014). All cell lines were cultured with 90% Roswell Park Memorial Institute-1640 (RPMI-1640; Hyclone, Logan, UT, USA) added with 10% fetal bovine serum (FBS, Gibco, Carlsbad, CA, USA) and 1% penicillin/streptomycin mixture (Sigma). A549/DDP and H1299/DDP cell lines were cultured with 1 μg/mL DDP to maintain DDP resistance.

### Cell counting kit-8 (CCK8) assay

A commercial CCK8 kit (Beyotime, Jiangsu, China) was used to analyze DDP resistance of NSCLC cells. Transfected NSCLC cells in 96-well plates were treated with DDP at the dose of 0.25, 0.5, 1, 2, 4, 8, or 16 μg/mL. Afterwards, NSCLC cells were incubated with 20 μL CCK8 reagent for 4 h. Cell viability was determined at the wavelength of 450 nm, and the 50% inhibiting concentration (IC50) value was then calculated. CCK8 assay was conducted three times with six biological repetitions each time.

### Reverse-transcription quantitative polymerase chain reaction (RT-qPCR)

RNA samples were isolated with Trizol reagent (Invitrogen, Waltham, MA, USA). For miR-524-5p, RNA was reversely transcribed using Mir-X^TM^ miRNA First-Strand Synthesis Kit (Takara, Dalian, China), and qPCR reaction was conducted using TaqMan MicroRNA Assay Kit (Applied Biosystems, Foster City, CA, USA). For circVMP1, METTL3, and SOX2, reverse transcription was performed using First-Strand cDNA Synthesis kit (Thermo Fisher Scientific, Waltham, MA, USA) and qPCR reaction was performed using SYBR Premix Ex Taq (Takara). All primers were shown in Table 1. The relative expression of circVMP1, miR-524-5p, METTL3, and SOX2 was analyzed by the 2^−ΔΔCt^ method. U6 and β-actin served as the internal references for miR-524-5p and circVMP1/METTL3/SOX2, respectively. RT-qPCR assay was conducted three times with three biological repetitions each time.

**Table 1.** Primer sequences used for qPCR.

Name		Primers for qPCR (5′–3′)
circVMP1（hsa_circ_0005077）	Forward	AGTCTGCACAACTCCTCAAGA
	Reverse	CAAGGTAATGAGCGGCTGTC
METTL3	Forward	TTGTCTCCAACCTTCCGTAGT
	Reverse	CCAGATCAGAGAGGTGGTGTAG
SOX2	Forward	TACAGCATGTCCTACTCGCAG
	Reverse	GAGGAAGAGGTAACCACAGGG
miR-524-5p	Forward	GTATGACTACAAAGGGAAGCAC
	Reverse	CTCAACTGGTGTCGTGGAG
U6	Forward	CTTCGGCAGCACATATACT
	Reverse	AAAATATGGAACGCTTCACG
β-actin	Forward	CTTCGCGGGCGACGAT
	Reverse	CCACATAGGAATCCTTCTGACC
18S rRNA	Forward	GGAGTATGGTTGCAAAGCTGA
	Reverse	ATCTGTCAATCCTGTCCGTGT

### RNase R degradation and subcellular fractionation

RNase R (Applied Biological Materials, Vancouver, Canada) was used to test the circular structure of circVMP1, and β-actin was used as the control. RNA samples were incubated with RNase R (100 μg/mL) at 37 °C for 20 min. RT-qPCR was conducted to detect the level of circVMP1.

The subcellular localization of circVMP1 was analyzed using the PARIS^TM^ Kit Protein and RNA Isolation system (Thermo Fisher Scientific), and U6 and 18 s rRNA were served as the nuclear and cytoplasmic controls, respectively.

### Cell transfection

Short hairpin (sh)RNA targeting circVMP1 (sh-circVMP1), shRNA targeting METTL3 (sh-METTL3), negative control of shRNA (sh-NC), mimics of miR-524-5p (miR-524-5p), miR-NC, inhibitor of miR-524-5p (anti-miR-524-5p), and anti-NC were purchased from GenePharma (Shanghai, China). Transient transfection was conducted with Lipofectamine 3000 (Invitrogen).

### 5-Ethynyl-2’-deoxyuridine (EdU) assay

NSCLC cells were incubated with 50 μM EdU reagent (RiboBio, Guangzhou, China) for 2 h for EdU incorporation during DNA synthesis. Afterwards, cell nucleus was stained with 4, 6-diamino-2-phenylindole (DAPI; Sigma). The fluorescence images were captured under a fluorescence microscope (Olympus, Tokyo, Japan). EdU assay was conducted three times with five biological repetitions each time.

### Sphere formation assay

NSCLC cells were seeded onto Clear Round Bottom Ultra Low Attachment Microplate (Corning) and incubated for 7 d. The diameter of spheres was assessed under a microscope. Sphere formation assay was conducted three times with three biological repetitions each time.

### Wound healing assay

NSCLC cells were seeded onto the 6-well plates. When cell confluence reached about 95%, 200 μL pipette tip was used to create the scratches on cell monolayer, and cell images were taken at 24 h after creating the scratches under an optical microscope. Wound healing assay was conducted three times with three biological repetitions each time.

### Transwell assay

Transwell plates coated with Matrigel reagent (BD Biosciences, Franklin Lakes, NJ, USA) were used to analyze cell invasion ability. Transfected NSCLC cells suspended in serum-free medium were added to the upper compartments, and the lower compartments were filled with culture medium plus 10% FBS. After cultivation for 24 h, cells still remaining on the upper compartments were removed with cotton swabs, and cells penetrated through the membrane were stained with 0.5% crystal violet (Sigma), and the number of invaded cells was manually counted under a light microscope (Olympus). Transwell assay was conducted three times with three biological repetitions each time.

### Flow cytometry

The apoptotic rate of NSCLC cells was analyzed by fluorescein isothiocyanate (FITC) and propidium iodide (PI) double-staining using a commercial Apoptosis Detection Kit (Qcbio Science & Technologies, Shanghai, China). Transfected NSCLC cells were collected with PBS and suspended in 100 μL of binding buffer. A total of 10 μL of Annexin V-FITC and PI were simultaneously added to the binding buffer to incubate with NSCLC cells for 15 min at room temperature in the dark. Cell samples were loaded onto the FACS CantoII flow cytometer (BD Biosciences), and cell apoptotic rate was assessed by BD FACSDiva software (BD Biosciences). Flow cytometry was conducted three times with three biological repetitions each time.

### Western blot assay

Protein samples were prepared with radio-immunoprecipitation assay (RIPA) buffer (Beyotime). The concentrations of protein samples were analyzed using the BCA method. Protein samples (35 μg/lane) were loaded onto 10% separating gel and blotted onto a PVDF membrane (Millipore, Billerica, MA, USA). After blocking with 5% skimmed milk for 1 h at room temperature, the membrane was incubated with the primary antibodies overnight at 4 °C, including anti-c-myc (ab32072, Abcam, Cambridge, MA, USA), anti-N-cadherin (ab280375, Abcam), anti-vimentin (ab92547, Abcam), anti-METTL3 (ab195352, Abcam), anti-SOX2 (ab92494, Abcam), anti-TSG101 (ab125011, Abcam), anti-CD63 (ab134045, Abcam), anti-CD81 (ab59477, Abcam), and anti-β-actin (ab8226, Abcam). Afterwards, the membrane was incubated with the secondary antibody (Abcam) for 1 h at room temperature. The protein bands were visualized using an enhanced chemiluminescence (ECL) kit (Pierce, Waltham, MA, USA), and the intensities of protein bands were analyzed using the Image Lab analysis software (National Institutes of Health, Bethesda, MD, USA). Western blot assay was conducted three times.

### Methylated RIP-qPCR (MeRIP-qPCR)

mRNA was fragmented and immuno-precipitated with Protein A beads (Thermo Fisher Scientific) and m^6^A antibody (ab151230; Abcam). Afterwards, the beads were washed with RIP buffer twice. After elution, RNA level was measured by qPCR. MeRIP-qPCR was conducted three times.

### Actinomycin D treatment

The stability of SOX2 mRNA was analyzed using transcriptional inhibitor Actinomycin D (Sigma). A549/DDP and H1299/DDP cells were incubated with 2 mg/mL Actinomycin D for 0 h, 2 h, 4 h, or 6 h. Cells were disrupted with Trizol reagent (Invitrogen), and RT-qPCR was applied to analyze RNA levels.

### Bioinformatics analysis

circBank database (http://www.circbank.cn) was used to predict the interacted miRNAs of circVMP1, and the interacted miRNAs of METTL3 and SOX2 were predicted by starBase database (http://starbase.sysu.edu.cn).

### RNA immunoprecipitation (RIP) assay

A commercial EZ-Magna RIP^TM^ RNA-Binding Protein Immunoprecipitation Kit (Millipore) was used to perform RIP assay. Cell extracts were mixed with beads coated with Argonaute2 (Ago2; Millipore) or Immunoglobulin G (IgG; Millipore) antibody. The enrichment of circVMP1, miR-524-5p, METTL3, and SOX2 was analyzed by RT-qPCR. RIP assay was performed three times.

### RNA-pull down assay

The probe for wild-type or mutant miR-524-5p with biotin labeling (bio-miR-524-5p-wt and bio-miR-524-5p-mut) was constructed by GenePharma. Cell lysates were incubated with the probe and beads (Invitrogen). The enrichment of circVMP1, METTL3, and SOX2 was analyzed by RT-qPCR. RNA pull down assay was performed three times.

### Dual-luciferase reporter assay

The fragment of circVMP1, METTL3 3’UTR, or SOX2 3’UTR, including the wild-type or mutant binding sites with miR-524-5p, was sub-cloned into pmirGLO (Promega, Madison, WI, USA). The constructed reporters were termed as circVMP1-wt/mut, METTL3 3’UTR-1-wt/mut, METTL3 3’UTR-2-wt/mut, and SOX2 3’UTR-wt/mut. 293 T cells were co-transfected with miR-524-5p or miR-NC and reporter plasmids. The luciferase intensities were determined using the dual-luciferase reporter assay kit (Promega). *Firefly* luciferase intensity was normalized to *Renilla* luciferase activity. Dual-luciferase reporter assay was conducted three times with three biological repetitions each time.

### The isolation and identification of exosomes

Exosomes were isolated from the culture supernatant of NSCLC cells or serum samples using a commercial ExoQuick precipitation kit (System Biosciences, Mountain view, CA, USA) according to the manufacturer’s instructions. The morphological characteristics of exosomes were captured under a transmission electron microscopy (TEM). The size distribution of exosomes was analyzed by Nanoparticle tracking analysis (NTA). Western blot assay was conducted to verify the presence of exosomal markers.

### Exosome uptake experiment

A total of 10 μM dioctadecyloxacarbocyanine (DIO) membrane dye (Sigma) was pipetted to the exosomal suspension in a 1:1 ratio for 30 min. NSCLC cells were incubated with 50 μg/mL DIO-stained exosomes for 30 min. Afterwards, the cells were immobilized with 4% paraformaldehyde (Sangon Biotech, Shanghai, China) for 10 min and then dyed with DAPI for 5 min. The uptake of exosome was observed under a fluorescence microscope.

### Xenograft tumor model

A total of 18 BALB/c female nude mice were purchased from Vital River Laboratory Animal Technology (Beijing, China). All nude mice were subcutaneously injected with A549 cells (2 × 10^6^). When tumor volume reached about 100 mm^3^, the nude mice were randomly divided into three groups (*n* = 6). From now on (day 0), DDP (20 mg/kg) was intraperitoneally injected into the mice twice a week, and exosome (10 μg) derived from A549/DDP cells transfected with sh-NC or sh-circVMP1 was intratumorally injected into the mice once every two days. Tumor dimension was analyzed every 5 d as length × width^2^×0.5. At 25 d, the nude mice were sacrificed by the CO_2_ asphyxia method, and tumors were weighed. Immunohistochemistry (IHC) assay was performed to analyze the protein levels of METTL3 and SOX2 in tumor tissues. Animal experiment was authorized by the Animal Ethical Committee of the First Affiliated Hospital of Soochow University.

### Clinical samples

Thirty-one DDP-resistant NSCLC patients and 22 DDP-sensitive NSCLC patients at the First Affiliated Hospital of Soochow University were recruited in the clinical study. A total of 5 mL of venous blood was collected by vena puncture, and serum samples were obtained through centrifugation at 1600 ×g for 10 min at room temperature followed by 12000 ×g for 10 min at 4 °C. All serum samples were stored at −80 °C. This study was authorized by the Ethical Committee of The First Affiliated Hospital of Soochow University. Written informed consent had been signed by all the participants before blood collection.

### Statistical analysis

All data were expressed as mean ± standard deviation. The statistical significances were analyzed by Student’s *t*-test or one-way analysis of variance (ANOVA) followed by Tukey’s test. *p <*.05 was considered statistically significant.

## Results

### CircVMP1 expression is up-regulated in DDP-resistant NSCLC cells

**Figure 1.**
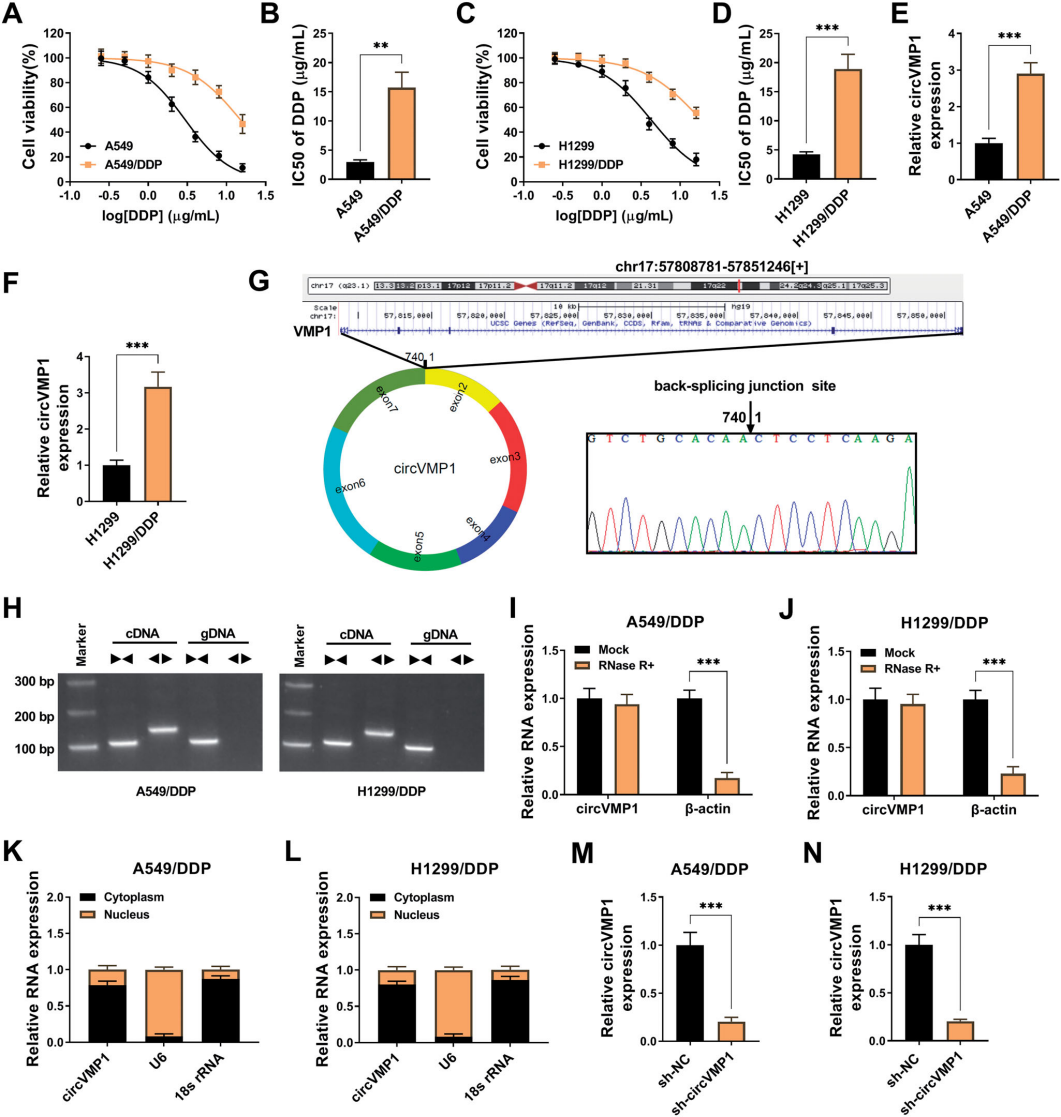
CircVMP1 expression is up-regulated in DDP-resistant NSCLC cells. (A and B) A549/DDP cell line was established, and CCK8 assay was performed to analyze the DDP resistance of A549/DDP and parental A549 cell lines. (C and D) H1299/DDP cell line was established, and the IC50 values of DDP in H1299/DDP and parental H1299 cell lines were determined by CCK8 assay. (E and F) The expression of circVMP1 was examined in DDP-resistant NSCLC cell lines (A549/DDP and H1299/DDP) and parental cell lines (A549 and H1299) by RT-qPCR. (G) A schematic diagram of the genomic location and splicing pattern of circVMP1. The back-splicing sites were verified by Sanger sequencing. (H) Divergent primers were used to confirm the circular structure of circVMP1. (I and J) The RNase R resistance of circVMP1 was analyzed, and the level of linear β-actin was used as the control. (K and L) The subcellular distribution of circVMP1 was analyzed, and U6 and 18 s rRNA were used as the internal references for cellular nucleus and cytoplasm. (M and N) The transfection efficiency of sh-circVMP1 was analyzed in A549/DDP and H1299/DDP cells by RT-qPCR. **p < .01, ***p < .001.

To explore the molecular mechanism of DDP resistance in NSCLC, we established two DDP-resistant NSCLC cell lines, including A549/DDP and H1299/DDP. DDP-resistant NSCLC cell lines and parental cell lines were treated with different doses of DDP (0.25, 0.5, 1, 2, 4, 8, or 16 μg/mL), and CCK8 assay was performed to analyze the IC50 values by measuring cell viability. The IC50 values of A549/DDP and H1299/DDP were significantly elevated compared with their parental cell lines (Figure 1(A–D)), suggesting the successful establishment of DDP-resistant NSCLC cell lines. CircVMP1 expression was notably up-regulated in A549/DDP and H1299/DDP cell lines compared with their parental cell lines (Figure 1(E,F)), implying the important role of circVMP1 in the development of DDP resistance. The genomic location of circVMP1 was shown in Figure 1(G). CircVMP1 was derived from the back-splicing of exon 2–7 of its host gene VMP1, and the existence of back-splicing sites was confirmed by Sanger sequencing (Figure 1(G)). CircVMP1 can be amplified by divergent primers in cDNA group, not in gDNA group (Figure 1(H)), suggesting its circular structure. CircVMP1 was resistant to RNase R+ (Figure 1(I,J)), further demonstrating the circular structure of circVMP1. CircVMP1 was majorly distributed in the cytoplasm of NSCLC cells (Figure 1(K,L)), suggesting that circVMP1 might function in post-transcriptional level. RT-qPCR assay verified the knockdown efficiency of sh-circVMP1 in NSCLC cells (Figure 1(M,N)). Taken together, circVMP1 might be implicated in the development of DDP resistance in NSCLC.

### CircVMP1 absence suppresses the proliferation, sphere formation, migration, invasion, and DDP resistance and induces the apoptosis of NSCLC cells

**Figure 2.**
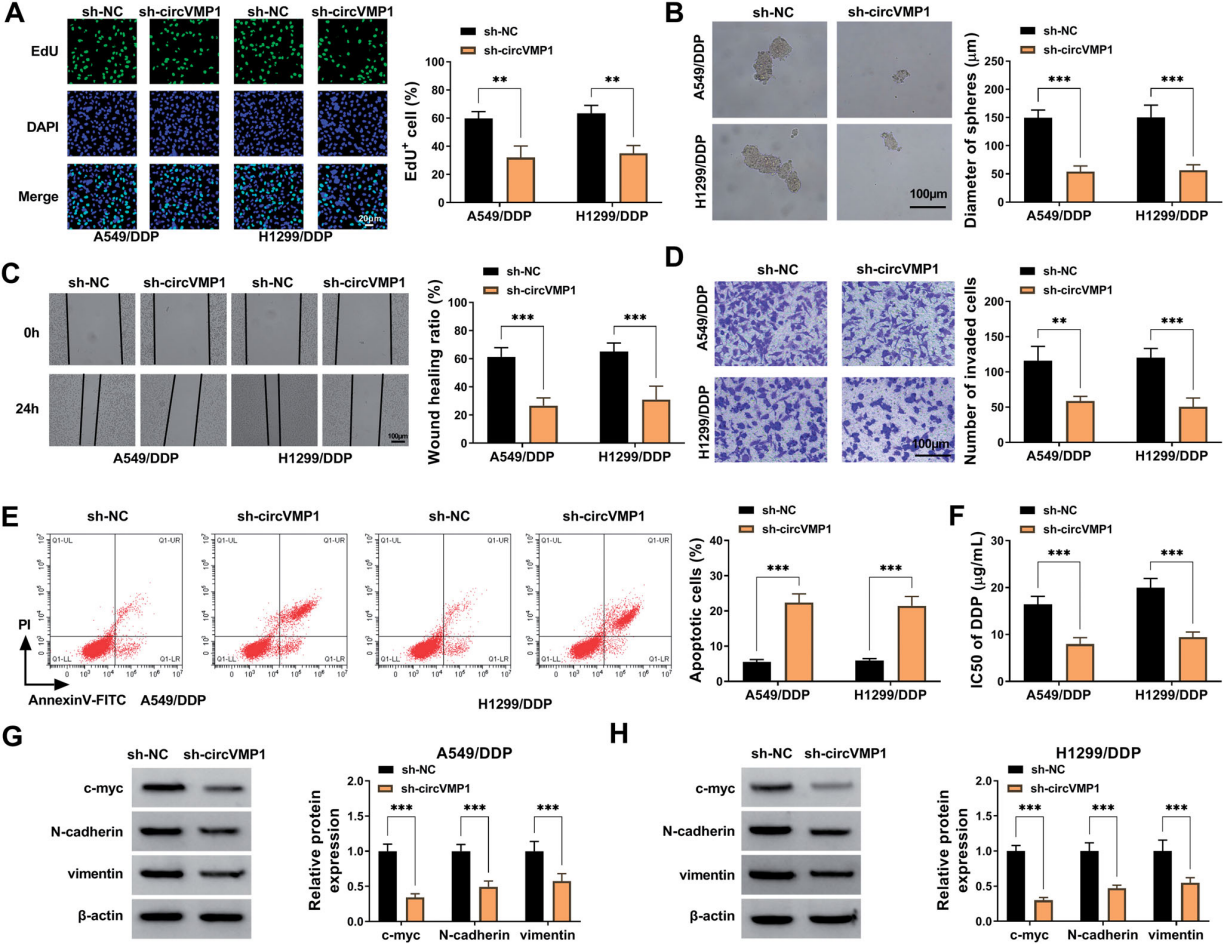
CircVMP1 absence suppresses the proliferation, sphere formation, migration, invasion, and DDP resistance and induces the apoptosis of NSCLC cells. (A–H) A549/DDP and H1299/DDP cells were transiently transfected with sh-NC or sh-circVMP1. (A) EdU assay was performed to analyze DNA synthesis rate of transfected A549/DDP and H1299 cells to assess cell proliferation ability. (B) Sphere formation assay was conducted to assess the sphere formation efficiency of transfected NSCLC cells to evaluate cell stemness. (C) Wound healing assay was performed to analyze the migration ability of transfected NSCLC cells. (D) Transwell assay was carried out to analyze the invasion ability of transfected NSCLC cells. (E) The apoptotic rate of transfected NSCLC cells was analyzed by flow cytometry. (F) CCK8 assay was performed to analyze the IC50 values of DDP in transfected A549/DDP and H1299/DDP cells. (G and H) Western blot assay was conducted to measure the protein levels of proliferation-associated indicator (c-myc) and EMT-associated indicators (N-cadherin and vimentin) in transfected NSCLC cells. **p < .01, ***p < .001.

We performed loss-of-function experiments to analyze the roles of circVMP1 in regulating NSCLC cell malignant behaviors and DDP resistance. Cell proliferation, sphere formation efficiency, migration, invasion, apoptosis, and DDP resistance were analyzed by EdU assay, sphere formation assay, wound healing assay, transwell assay, flow cytometry, and CCK8 assay. CircVMP1 absence conspicuously reduced the incorporation of EdU in the genome of A549/DDP and H1299/DDP cells (Figure 2(A)), suggesting that circVMP1 silencing inhibited the proliferation of NSCLC cells. The sphere formation efficiency of NSCLC cells was reduced by silencing circVMP1 (Figure 2(B)), indicating that circVMP1 knockdown restrained the stemness of NSCLC cells. The distance of cell migration was deceased in circVMP1-silenced group (Figure 2(C)), indicating that circVMP1 absence restrained the migration of NSCLC cells. Transwell assay revealed that circVMP1 interference reduced the number of invaded cells (Figure 2(D)), demonstrating that circVMP1 absence inhibited the invasion ability of NSCLC cells. CircVMP1 knockdown markedly induced the apoptosis of NSCLC cells (Figure 2(E)). Subsequently, we analyzed the effect of circVMP1 silencing on the chemo-resistance of A549/DDP and H1299/DDP cells by CCK8 assay. The data showed that circVMP1 knockdown significantly reduced the IC50 value of DDP (Figure 2(F)), proving that circVMP1 absence elevated the DDP sensitivity of A549/DDP and H1299/DDP cells. The levels of proliferation-associated indicator c-myc and epithelial-mesenchymal transition (EMT)-associated indicators (N-cadherin and vimentin) were detected in transfected NSCLC cells by Western blot assay. The results uncovered that circVMP1 absence reduced the expression of c-myc, N-cadherin, and vimentin (Figure 2(G,H)). Overall, circVMP1 played an oncogenic role in NSCLC cells.

### CircVMP1 silencing reduces the protein expression of METTL3 and SOX2 in NSCLC cells

**Figure 3.**
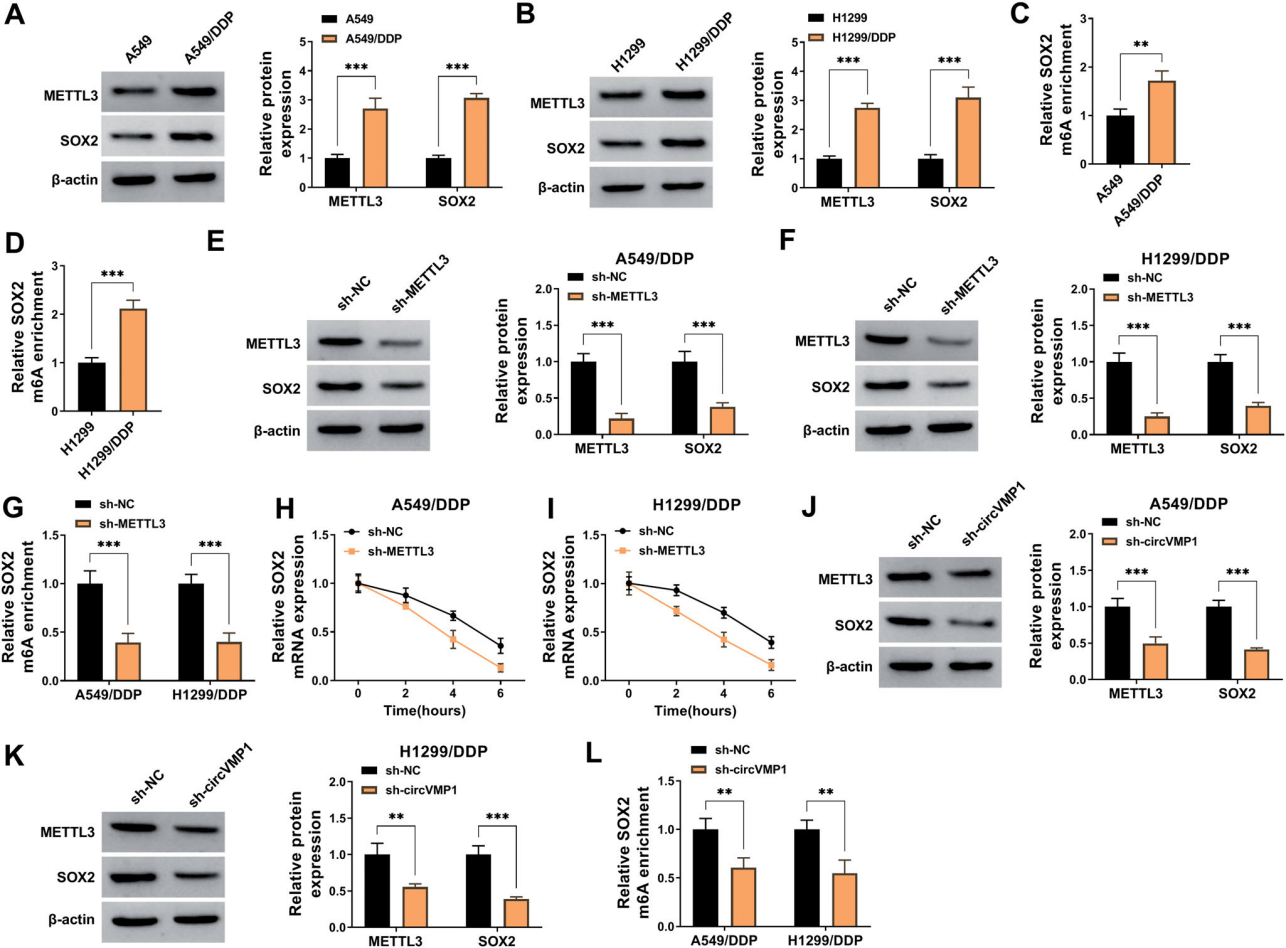
CircVMP1 silencing reduces the protein expression of METTL3 and SOX2 in NSCLC cells. (A and B) Western blot assay was performed to detect the protein expression of METTL3 and SOX2 in DDP-resistant NSCLC cell lines (A549/DDP and H1299/DDP) and parental NSCLC cell lines (A549 and H1299). (C) MeRIP-qPCR assay was performed to analyze the m^6^A modification level of SOX2 in A549 and A549/DDP cell lines. (D) The m^6^A modification level of SOX2 in H1299 and H1299/DDP cell lines was assessed by MeRIP-qPCR assay. (E and F) Western blot assay was conducted to analyze the protein levels of METTL3 and SOX2 in A549/DDP and H1299/DDP cells transfected with sh-NC or sh-METTL3. (G) MeRIP-qPCR assay was implemented to analyze the m^6^A modification level of SOX2 in A549/DDP and H1299/DDP cells transfected with sh-NC or sh-METTL3. (H and I) Transcriptional inhibitor Actinomycin D was used to analyze the stability of SOX2 mRNA in A549/DDP and H1299/DDP cells transfected with sh-NC or sh-METTL3. (J and K) The protein levels of METTL3 and SOX2 were determined in A549/DDP and H11299/DDP cells transfected with sh-NC or sh-circVMP1 by Western blot assay. (L) The m^6^A modification level of SOX2 in A549/DDP and H1299/DDP cells transfected with sh-NC or sh-circVMP1 was assessed by MeRIP-qPCR assay. **p < .01, ***p < .001.

N6-methylladenosine (m^6^A) is a specific manner of RNA modification, which is ubiquitous in many eukaryotes (Rottman et al., 1976; Wang et al., 2018). m^6^A methylation is reported to function throughout the whole RNA life cycle, such as RNA splicing, nuclear transportation, RNA stability, and translation (Shafik et al., 2016; Roignant & Soller, 2017; Yang et al., 2017). The dysregulation of m^6^A methylation has shown to be associated with multiple pathological processes, including chemo-resistance (Geula et al., 2015). METTL3 is one of the most well-known m^6^A methyltransferases and is reported to contribute to the DDP resistance of bladder cancer, oral cancer, and seminoma through its methyltransferase activity (Wei et al., 2020; Qiao et al., 2021; Wei et al., 2021). SOX2 is a key transcription factor that plays an important role in maintaining stem cell characteristics and endowing drug resistance (Chaudhary et al., 2019; Mamun et al., 2020). Moreover, previous studies showed that METTL3 could elevate the stability of SOX2 by increasing its m^6^A level, thus contributing to the progression of glioma and colorectal cancer (Visvanathan et al., 2018; Li et al., 2019). In our study, Western blot assay showed that METTL3 and SOX2 were up-regulated in DDP-resistant NSCLC cell lines compared with sensitive cell lines (Figure 3(A,B)). MeRIP-qPCR assay revealed that the m^6^A modification level of SOX2 was elevated in A549/DDP and H1299/DDP cells compared with sensitive cell lines (Figure 3(C,D)). Transfection with sh-METTL3 significantly decreased the protein levels of METTL3 and SOX2 in NSCLC cells (Figure 3(E,F)). METTL3 absence also reduced the m^6^A modification level of SOX2 in NSCLC cells (Figure 3(G)). To analyze the role of METTL3 in regulating the stability of SOX2 mRNA, transcriptional inhibitor Actinomycin D was used. The results revealed that METTL3 silencing reduced the stability of SOX2 (Figure 3(H,I)). These data showed that METTL3 enhanced the stability of SOX2 mRNA by increasing its m^6^A modification level. Subsequently, we analyzed the regulatory relationship between circVMP1 and METTL3 or SOX2 in NSCLC cells. CircVMP1 silencing markedly down-regulated the protein levels of METTL3 and SOX2 in NSCLC cells (Figure 3(J,K)). Meanwhile, the m^6^A modification level of SOX2 was also decreased by silencing circVMP1 in NSCLC cells (Figure 3(L)). Taken together, circVMP1 can positively regulate the expression of METTL3 and SOX2 in NSCLC cells.

### CircVMP1-miR-524-5p-METTL3/SOX2 axis is established in NSCLC cells

**Figure 4.**
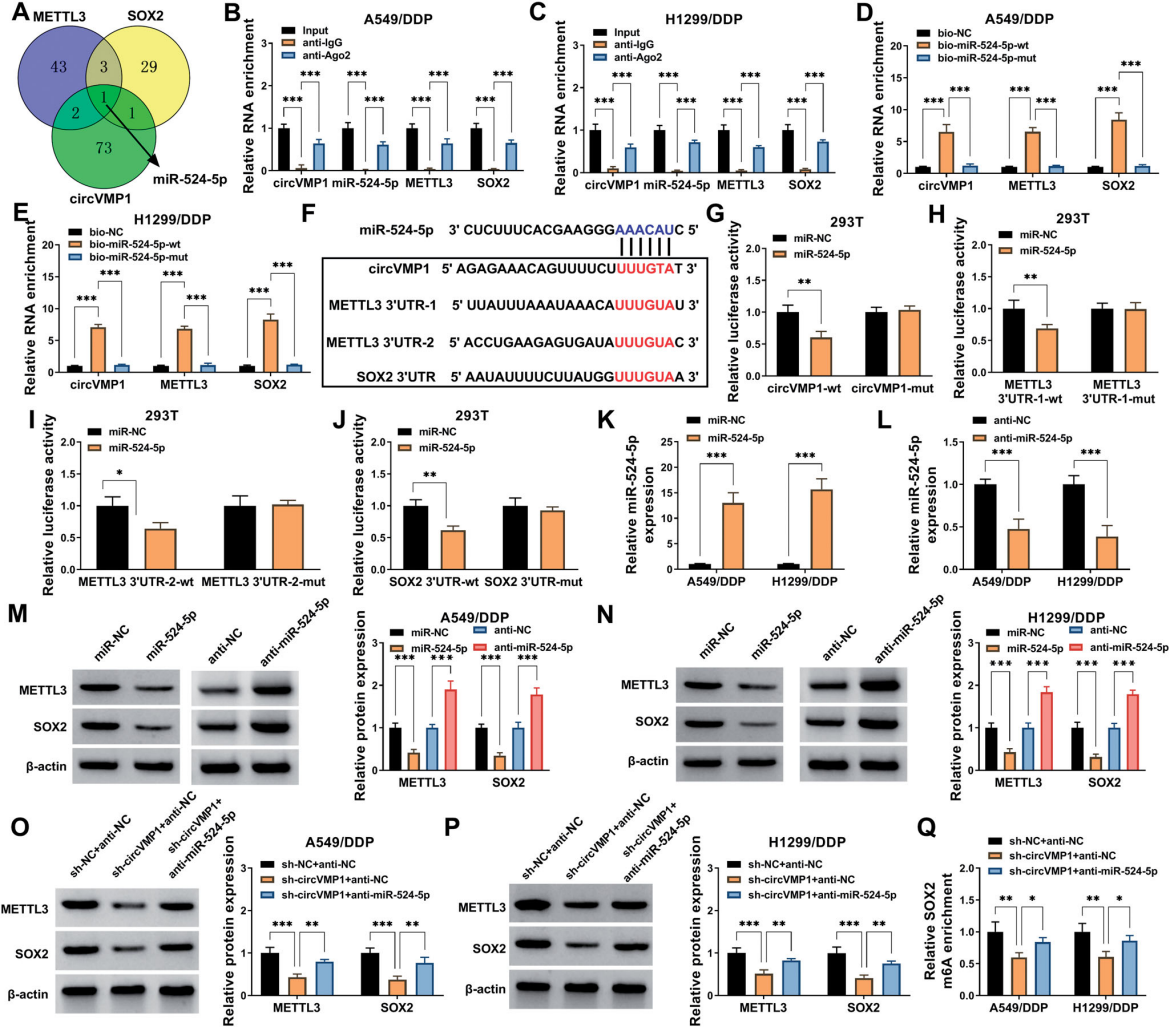
CircVMP1 can positively regulate the expression of METTL3 and SOX2 by sponging miR-524-5p in NSCLC cells. (A) circBank database was used to predict the interacted miRNAs of circVMP1, and the interacted miRNAs of METTL3 and SOX2 were predicted by starBase database. Venn diagram showed the predicted common miRNA targets of circVMP1, METTL3, and SOX2. (B and C) RIP assay was performed to analyze the interaction between miR-524-5p and circVMP1, METTL3, or SOX2. (D and E) RNA-pull down assay was performed to confirm the target relationship between miR-524-5p and circVMP1, METTL3, or SOX2. (F) The putative binding sites between miR-524-5p and circVMP1, METTL3, or SOX2 were shown. (G–J) The interaction between miR-524-5p and circVMP1, METTL3, or SOX2 was verified by dual-luciferase reporter assay. (K) RT-qPCR was conducted to analyze the overexpression efficiency of miR-524-5p mimics in NSCLC cells. (L) The knockdown efficiency of anti-miR-524-5p was analyzed in NSCLC cells by RT-qPCR. (M and N) A549/DDP and H1299/DDP cells were transfected with miR-NC, miR-524-5p, anti-NC, or anti-miR-524-5p, and the protein expression of METTL3 and SOX2 was detected by Western blot assay. (O and P) The protein levels of METTL3 and SOX2 were determined in A549/DDP and H1299/DDP cells transfected with sh-NC + anti-NC, sh-circVMP1 + anti-NC, or sh-circVMP1 + anti-miR-524-5p by Western blot assay. (Q) The m^6^A modification level of SOX2 in A549/DDP and H1299/DDP cells transfected with sh-NC + anti-NC, sh-circVMP1 + anti-NC, or sh-circVMP1 + anti-miR-524-5p was analyzed by MeRIP-qPCR assay. *p < .05, **p < .01, ***p < .001.

It has been widely accepted that circRNAs can act as miRNA sponges to release the downstream mRNAs from the inhibition of miRNAs, resulting in the up-regulation of mRNAs (Qi et al., 2015; Liang et al., 2020). To explore the working mechanism by which circVMP1 positively regulated the expression of METTL3 and SOX2, the interacted miRNAs were predicted by bioinformatics analysis. circBank database was used to predict the interacted miRNAs of circVMP1, and the interacted miRNAs of METTL3 and SOX2 were predicted by starBase database. As shown in Figure 4(A), Venn diagram showed that miR-524-5p was the only miRNA that could simultaneously interact with circVMP1, METTL3, and SOX2. RIP assay revealed that circVMP1, miR-524-5p, METTL3, and SOX2 were all enriched in precipitated complex when using Ago2 antibody (Figure 4(B,C)). RNA-pull down assay uncovered that circVMP1, METTL3, and SOX2 were all pulled down when using wild-type miR-524-5p probe (bio-miR-524-5p wt) (Figure 4(D,E)), further demonstrating the intermolecular interaction between miR-524-5p and circVMP1, METTL3, or SOX2. The putative binding sequence between miR-524-5p and circVMP1, METTL3, or SOX2 was shown in Figure 4(F). Dual-luciferase reporter assay revealed that miR-524-5p overexpression markedly reduced the luciferase activity of wild-type reporter (circVMP1-wt) but not that of mutant reporter (circVMP1-mut) (Figure 4(G)), suggesting that circVMP1 interacted with miR-524-5p via the predicted sequence. Similarly, we found that miR-524-5p interacted with METTL3 via the two predicted sequences (Figure 4(H,I)). miR-524-5p was also found to interact with SOX2 via the putative sites (Figure 4(J)). RT-qPCR confirmed the transfection efficiencies of miR-524-5p mimics and anti-miR-524-5p in NSCLC cells (Figure 4(K,L)). miR-524-5p overexpression reduced the protein expression of METTL3 and SOX2 in A549/DDP and H1299/DDP cells, while miR-524-5p knockdown cased the opposite effects (Figure 4(M,N)), suggesting that miR-524-5p negatively regulated the protein expression of METTL3 and SOX2 in NSCLC cells. CircVMP1 absence reduced the protein expression of METTL3 and SOX2, and the addition of anti-miR-524-5p largely restored the protein levels of METTL3 and SOX2 in NSCLC cells (Figure 4(O,P)). The m^6^A modification level of SOX2 was reduced by circVMP1 absence, and the addition of anti-miR-524-5p largely rescued the m^6^A modification level of SOX2 in NSCLC cells (Figure 4(Q)). These results suggested that circVMP1 acted as miR-524-5p sponge to up-regulate METTL3 and SOX2 in NSCLC cells.

### CircVMP1 absence restrains NSCLC progression and DDP resistance partly by up-regulating miR-524-5p *in vitro*

**Figure 5.**
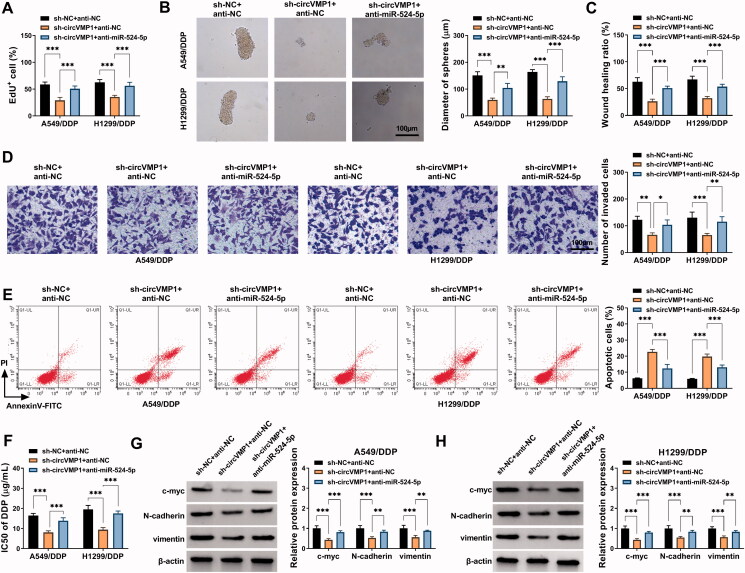
CircVMP1 absence restrains the malignant behaviors and chemo-resistance of DDP-resistant NSCLC cells partly by up-regulating miR-524-5p. (A–H) A549/DDP and H1299/DDP cells were transfected with sh-NC + anti-NC, sh-circVMP1 + anti-NC, or sh-circVMP1 + anti-miR-524-5p. (A) Cell proliferation ability was analyzed by EdU assay. (B) The sphere formation efficiency of NSCLC cells was analyzed by sphere formation assay. (C and D) Wound healing assay and transwell assay were performed to assess the migration and invasion abilities of NSCLC cells. (E) Flow cytometry was conducted to analyze the apoptotic rate of NSCLC cells. (F) CCK8 assay was carried out to analyze the I550 value of DDP in transfected NSCLC cells. (G and H) The protein levels of c-myc, N-cadherin, and vimentin in transfected NSCLC cells were measured by Western blot assay. *p < .05, **p < .01, ***p < .001.

We performed rescue experiments to explore whether circVMP1 regulated the malignant behaviors and DDP resistance of NSCLC cells by targeting miR-524-5p. A549/DDP and H1299/DDP cells were transfected with sh-NC + anti-NC, sh-circVMP1 + anti-NC, or sh-circVMP1 + anti-miR-524-5p. CircVMP1 silencing-induced suppressive effects on the proliferation and sphere formation efficiency of NSCLC cells were largely counteracted by the addition of anti-miR-524-5p (Figure 5(A,B)). The introduction of anti-miR-524-5p also largely restored the migration and invasion abilities in circVMP1-silenced NSCLC cells (Figure 5(C,D)). CircVMP1 absence-induced cell apoptosis was largely attenuated by the introduction of anti-miR-524-5p (Figure 5(E)). CircVMP1 knockdown suppressed the DDP resistance of NSCLC cells, and the chemo-resistance of NSCLC cells was largely recovered in sh-circVMP1 and anti-miR-524-5p co-transfected group (Figure 5(F)). The introduction of anti-miR-524-5p also largely restored the protein levels of c-myc, N-cadherin, and vimentin in circVMP1-silenced NSCLC cells (Figure 5(G,H)). Taken together, circVMP1 silencing-mediated anti-tumor effects in NSCLC cells were largely based on the up-regulation of miR-524-5p.

### DDP-resistant NSCLC cells transmit circVMP1 to parental NSCLC cells through exosomes

**Figure 6.**
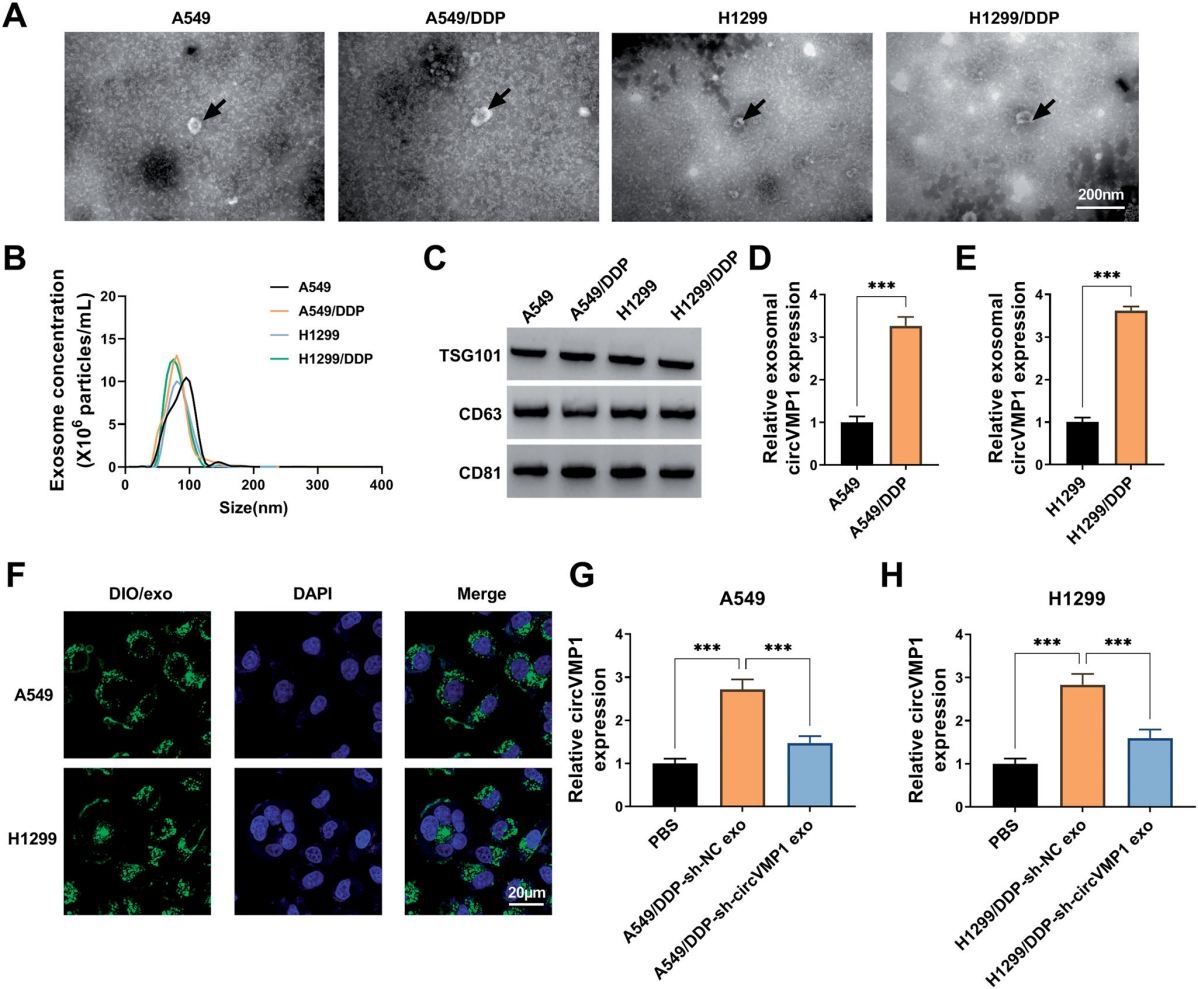
DDP-resistant NSCLC cells transmit circVMP1 to parental NSCLC cells through exosomes. (A) TEM scanning showed the images of exosomes derived from A549, A549/DDP, H1299, and H1299/DDP cells. (B) NTA revealed the size distribution of exosomes released by A549, A549/DDP, H1299, and H1299/DDP cells. (C) Western blot assay was conducted to measure the expression of exosomal marker proteins (TSG101, CD63, and CD81). (D and E) RT-qPCR was conducted to measure the expression of exosomal circVMP1 released by A549, A549/DDP, H1299, and H1299/DDP cells. (F) A549 and H1299 cells were incubated with DIO-stained exosomes for 30 min, and then stained with DAPI for 5 min. The uptake of exosome was observed under a fluorescence microscope. (G and H) A549 and H1299 cells were incubated with exosomes derived from A549/DDP or H1299/DDP cells transfected with sh-NC or sh-circVMP1. The level of circVMP1 in recipient cells was determined by RT-qPCR. ***p < .001.

The morphological characteristics of exosomes released by A549, A549/DDP, H1299, and H1299/DDP cells were observed by TEM. Exosomes were round or oval membranous vesicle with the diameter of 30-60 nm (Figure 6(A)). NTA revealed the size distribution and concentration of the isolated exosomes (Figure 6(B)). The presence of exosomal protein markers (TSG101, CD63, and CD81) was verified by Western blot assay (Figure 6(C)). It was observed that the expression of circVMP1 was prominently up-regulated in exosomes derived from DDP-resistant cell lines compared with that in parental cell lines (Figure 6(D,E)). A549 and H1299 cells were incubated with DIO-stained exosomes, and the uptake of exosomes was observed under a fluorescence microscope. As shown in Figure 6(F), it was observed that exosomes can be taken up by recipient cells. Parental NSCLC cells were incubated with exosomes released by A549/DDP or H1299/DDP cells transfected with sh-NC or sh-circVMP1. The data revealed that the expression of circVMP1 in recipient A549 and H1299 cells was markedly up-regulated after incubating with exosomes derived from A549/DDP-sh-NC and H1299/DDP-sh-NC (Figure 6(G,H)). CircVMP1-silenced DDP-resistant NSCLC cells-derived exosomes transmitted fewer circVMP1 that that in sh-NC-transfected cells (Figure 6(G,H)). Overall, these data revealed that DDP-resistant NSCLC cells transmit circVMP1 to parental NSCLC cells via exosomes.

### Exosome-mediated transfer of circVMP1 disseminates the chemo-resistance

**Figure 7.**
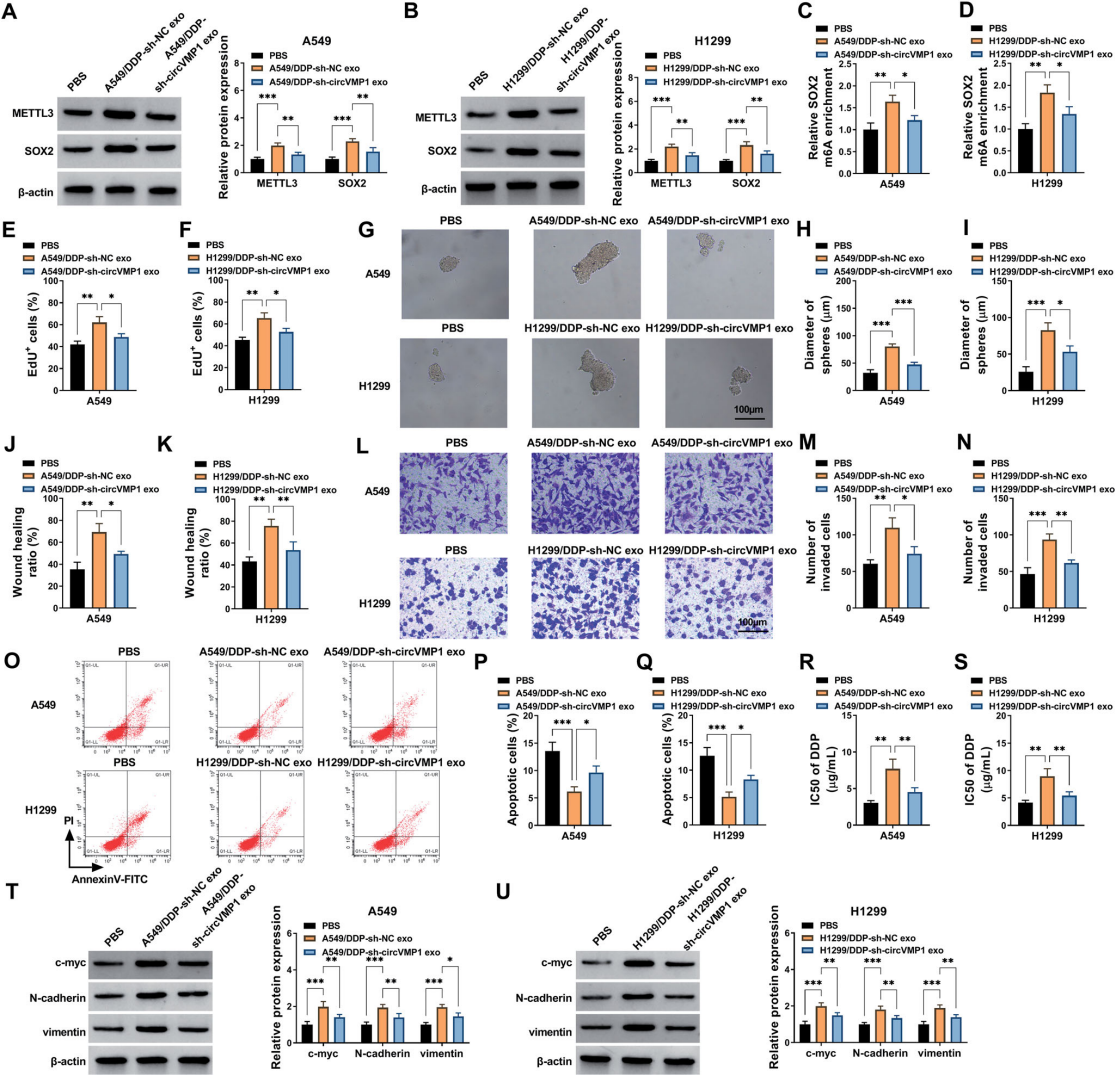
Exosome-mediated transfer of circVMP1 disseminates the chemo-resistance. (A–U) A549 and H1299 cells were incubated with exosomes derived from A549/DDP or H1299/DDP cells transfected with sh-NC or sh-circVMP1. (A and B) The protein expression of METTL3 and SOX2 was determined in recipient cells by Western blot assay. (C and D) MeRIP-qPCR assay was conducted to analyze the m^6^A modification level of SOX2. (E and F) EdU assay was conducted to assess the proliferation ability of NSCLC cells. (G-I) Sphere formation assay was performed to analyze the sphere formation efficiency of NSCLC cells. (J and K) Wound healing assay was carried out to analyze the migration ability of NSCLC cells. (L–N) Transwell assay was performed to assess the invasion capacity of NSCLC cells. (O–Q) Flow cytometry was conducted to analyze the apoptosis of NSCLC cells. (R and S) The DDP resistance of NSCLC cells was analyzed by CCK8 assay. (T and U) Western blot assay was employed to measure the protein levels of c-myc, N-cadherin, and vimentin in NSCLC cells. *p < .05, **p < .01, ***p < .001.

Considering the oncogenic role of circVMP1 in NSCLC cells, we investigated whether DDP-resistant NSCLC cells-derived exosomal circVMP1 can elevate the malignant behaviors and DDP resistance of parental NSCLC cells. The protein expression of METTL3 and SOX2 was markedly up-regulated in recipient NSCLC cells after incubating with circVMP1-enriched exosomes (Figure 7(A,B)). Meanwhile, the m^6^A modification level of SOX2 was also up-regulated in recipient NSCLC cells after incubating with circVMP1-enriched exosomes (Figure 7(C,D)). Exosomal circVMP1 promoted the proliferation and sphere formation efficiency of parental NSCLC cells (Figure 7(E–I)). Exosomal circVMP1 facilitated the migration and invasion abilities and suppressed the apoptosis of parental NSCLC cells (Figure 7(J–Q)). We also found that exosomal circVMP1 elevated the DDP resistance of parental A549 and H1299 cells (Figure 7(R,S)). Exosomal circVMP1 up-regulated the expression of proliferation-associated indicator (c-myc) and EMT-related indicators (N-cadherin and vimentin) in parental NSCLC cells (Figure 7(T,U)). Taken together, DDP-resistant NSCLC cells-derived exosomal circVMP1 disseminated the malignant potential and chemo-resistance to parental NSCLC cells.

### Exosomal circVMP1 elevates the DDP resistance of xenograft tumors *in vivo*

**Figure 8.**
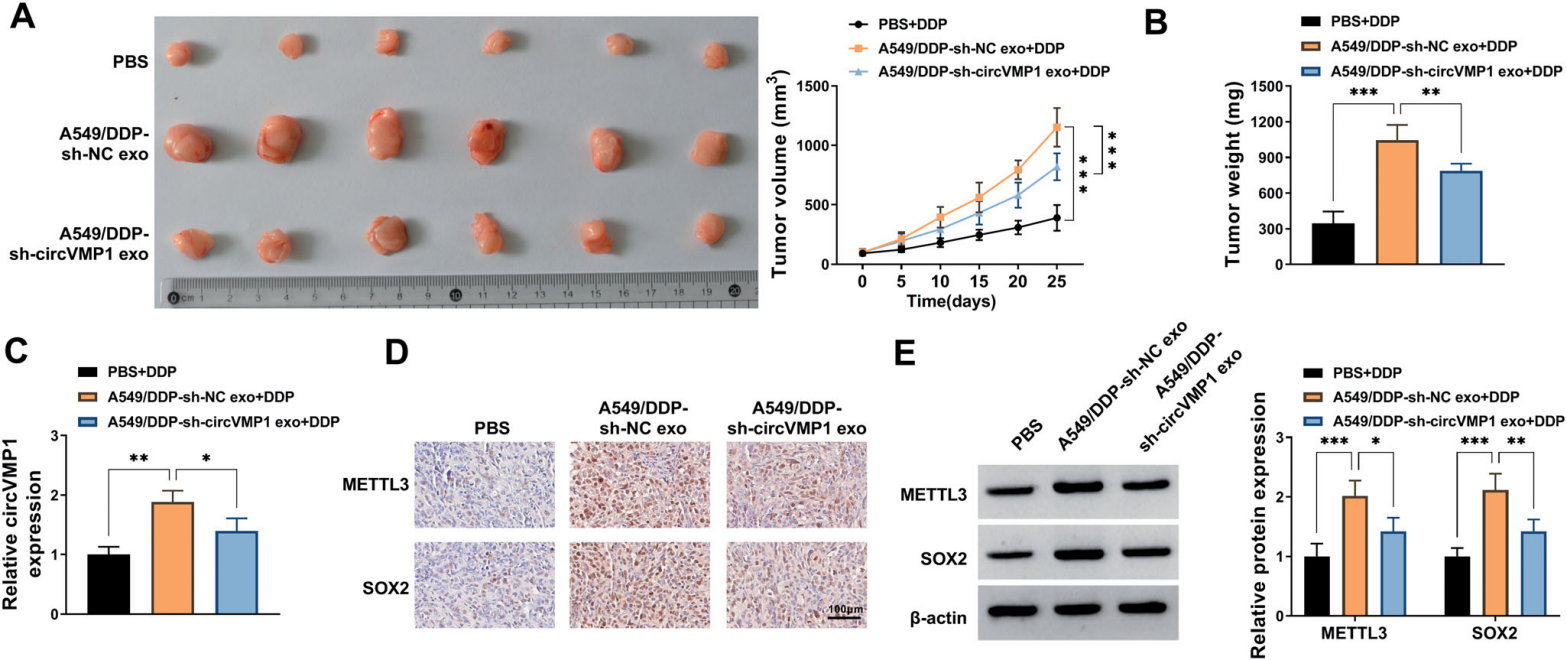
Exosomal circVMP1 elevates the DDP resistance of NSCLC cells *in vivo*. All nude mice were subcutaneously injected with A549 cells (2 × 10^6^). When tumor volume reached about 100 mm^3^, the nude mice were randomly divided into three groups (*n* = 6). DDP (20 mg/kg) was intraperitoneally injected into the mice twice a week, and exosome (10 μg) derived from A549/DDP cells transfected with sh-NC or sh-circVMP1 was intratumorally injected into the mice once every two days. (A) Tumor volume was monitored every 5 d as length × width^2^×0.5. (B) The nude mice were sacrificed at 25 d, and tumor weight was recorded. (C) RT-qPCR was performed to measure the expression of circVMP1 in tumor tissues in three groups. (D) IHC assay was carried out to analyze the protein levels of METTL3 and SOX2 in tumor tissues in three groups. (E) Western blot assay was conducted to analyze the protein levels of METTL3 and SOX2 in tumor tissues in three groups. *p < .05, **p < .01, ***p < .001.

To investigate the role of exosomal circVMP1 on the DDP resistance of parental NSCLC cells *in vivo*, we established xenograft tumor model in nude mice. The nude mice were subcutaneously injected with A549 cells (2 × 10^6^) and then injected with DDP (20 mg/kg) twice a week and exosome (10 μg) once every two days. After incubation with exosomes derived from sh-NC-transfected A549/DDP cells, tumors exhibited more DDP resistance than that in PBS + DDP group and A549/DDP + sh-circVMP1 exo + DDP group (Figure 8(A,B)), suggesting that exosomal circVMP1 disseminated DDP resistance to parental NSCLC cells *in vivo*. The expression of circVMP1 in tumor tissues was elevated after injecting with exosomes derived from sh-NC-transfected A549/DDP cells (Figure 8(C)). IHC assay and Western blot assay revealed that the protein levels of METTL3 and SOX2 were both up-regulated in A549/DDP-sh-NC exo group (Figure 8(D,E)). Taken together, exosomal circVMP1 elevated the DDP resistance of parental NSCLC cells *in vivo*.

### The high expression of circVMP1 in serum exosomes may be a novel bio-marker for DDP resistance in NSCLC patients

**Figure 9.**
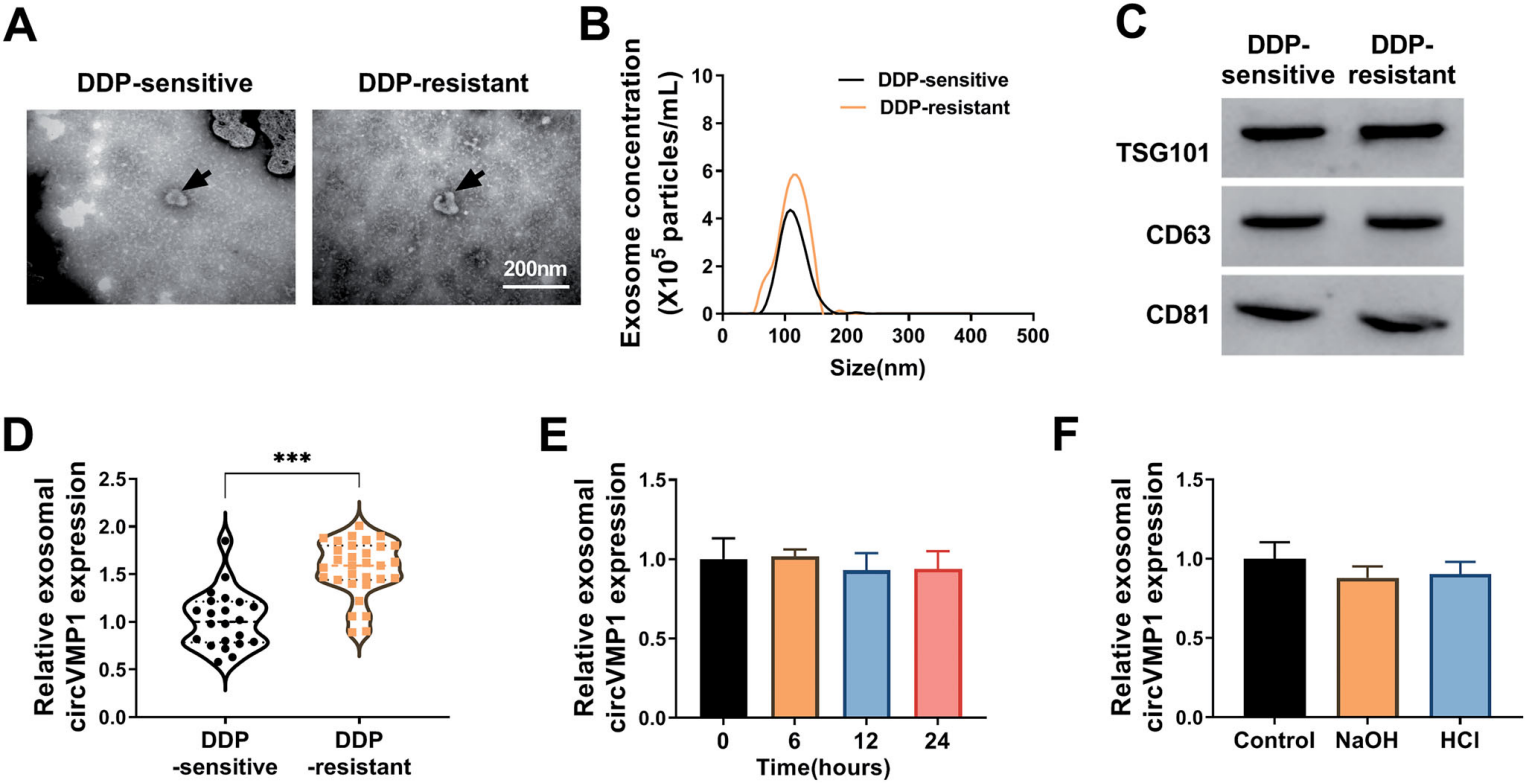
CircVMP1 expression is markedly increased in the serum exosomes of DDP-resistant NSCLC patients compared with DDP-sensitive NSCLC patients. (A) The TEM images of serum exosomes in DDP-resistant/sensitive NSCLC patients were shown. (B) NTA uncovered the size and concentrations of serum exosomes in DDP-resistant/sensitive NSCLC patients. (C) Western blot assay was carried out to measure the expression of exosomal marker proteins (TSG101, CD63, and CD81). (D) RT-qPCR was performed to analyze the expression of circVMP1 in the serum exosomes in DDP-resistant/sensitive NSCLC patients. (E and F) Serum exosomes were exposed to different conditions including incubation at room temperature (0, 6, 12, or 24 h) and low (pH = 1) or high (pH = 13) pH solution for 3 h at room temperature. The stability of circVMP1 was analyzed by RT-qPCR. ***p < .001.

We further explored whether serum exosomal circVMP1 could serve as a novel bio-marker for DDP sensitivity in NSCLC patients. TEM revealed the morphological characteristics of serum exosomes derived from DDP-resistant and sensitive NSCLC patients (Figure 9(A)). NTA uncovered the size distribution and concentration of serum exosomes in DDP-resistant NSCLC patients and DDP-sensitive patients (Figure 9(B)). Western blot assay confirmed the presence of exosomal markers (TSG101, CD63, and CD81) (Figure 9(C)). The expression of exosomal circVMP1 was markedly up-regulated in the serum samples of DDP-resistant NSCLC patients compared with that in DDP-sensitive patients, which was consistent with the results in cellular level (Figure 9(D)). We then assessed the stability of circVMP1 in the serum exosomes by exposing exosomes to different conditions including incubation at room temperature (0, 6, 12, or 24 h) and low (pH = 1) or high (pH = 13) pH solution for 3 h at room temperature. The results showed that circVMP1 level was unaffected by any of the experimental conditions (Figure 9(E,F)). These results demonstrated that exosomal circVMP1 was stably existed in serum, and it might be a novel promising bio-marker for NSCLC patients.

## Discussion

Exosomes are rich in circRNAs, which are considered as novel noninvasive tumor diagnostic biomarkers (Fanale et al., 2018). Previous studies have demonstrated that tumor-derived exosomal circRNAs promote the chemo-resistance and progression of NSCLC (Ding et al., 2020; Xu et al., 2020). For instance, Xu et al., reported that exosomes-transmitted circ_0014235 facilitates the chemo-resistance and development of NSCLC by targeting miR-520a-5p/CDK4 cascade (Xu et al., 2020). A previous study reported that circVMP1 is up-regulated in the serum exosomes in patients with lung adenocarcinoma compared with healthy controls (Lin et al., 2020). Nevertheless, the biological function of circVMP1 in NSCLC progression remains to be clarified. We established A549/DDP and H1299/DDP cell lines, and observed that circVMP1 expression was conspicuously elevated in DDP-resistant NSCLC cell lines. To explore the biological role of circVMP1, we conducted a series of knockdown experiments. CircVMP1 silencing restrained the proliferation, sphere formation, motility, and triggered the apoptosis of DDP-resistant NSCLC cells. Meanwhile, circVMP1 absence also sensitized DDP-resistant NSCLC cells to DDP.

N6-methyladenosine (m^6^A) is the most common post-transcriptional modification in mRNA, which controls the fate of mRNA by regulating various metabolic processes such as mRNA splicing, translation, and degradation (Fustin et al., 2013; Lin et al., 2016). METTL3 is a key member of the m^6^A methyltransferase complex, and is also a pivotal oncogene for tumor development in multiple malignancies (Zhang et al., 2017; Chen et al., 2018; Weng et al., 2018). SOX2 is a key transcription factor that maintains stem cell characteristics and endows drug resistance (Chaudhary et al., 2019; Mamun et al., 2020). SOX2 is identified as a downstream target of METTL3, and METTL3 is reported to maintain the expression of SOX2 by facilitating its methylation, thereby leading to the progression of glioma (Visvanathan et al., 2018) and colorectal cancer (Li et al., 2019). Here, METTL3 and SOX2 expression was increased in DDP-resistant NSCLC cells. Furthermore, the m^6^A modification level of SOX2 was also elevated in DD-resistant NSCLC cells. After transfecting with sh-METTL3, the protein level of METTL3 was reduced, and the m^6^A modification level of SOX2 was decreased, resulting in the degradation of SOX2 mRNA and the down-regulation of SOX2 protein level. We further observed that circVMP1 absence reduced the protein levels of METTL3 and SOX2 and the m^6^A modification level of SOX2 in NSCLC cells.

CircRNAs/miRNAs/mRNAs axis has been established in numerous studies to illustrate the working mechanism of circRNAs in multiple biological processes (Li et al., 2019; Gong et al., 2020; Liang et al., 2020). To explore the mechanism by which circVMP1 regulated the expression of METTL3 and SOX2, bioinformatics analysis was conducted. miR-524-5p was the only miRNA that was predicted to interact simultaneously with circVMP1, METTL3, and SOX2, and these intermolecular interactions were then verified by functional experiments. We found that circVMP1 positively regulated the expression of METTL3 and SOX2 by sequestering miR-524-5p in NSCLC cells. Through rescue experiments, it was observed that circVMP1 absence suppressed the malignant behaviors and DDP resistance of A549/DDP and H1299/DDP cells partly by up-regulating miR-524-5p.

CircVMP1 expression was prominently elevated in DDP-resistant NSCLC cells, and we further investigated whether DDP-resistant NSCLC cells-derived exosomes could transmit circVMP1 to sensitive cells, thereby regulating the behaviors and DDP resistance of sensitive cells. The results revealed that DDP-resistant NSCLC cells-derived exosomal circVMP1 can be transmitted to sensitive cells, and exosomal circVMP1 elevated the malignant behaviors and DDP resistance of sensitive cells. Moreover, DDP-resistant NSCLC cells-derived exosomal circVMP1 also increased the DDP resistance of xenograft tumors *in vivo*.

We observed that the level of serum exosomal circVMP1 was notably up-regulated in DDP-resistant NSCLC patients. Additionally, exosomal circVMP1 was stable in the serum samples. Therefore, it is a potential promising bio-marker for NSCLC patients.

**Figure 10.**
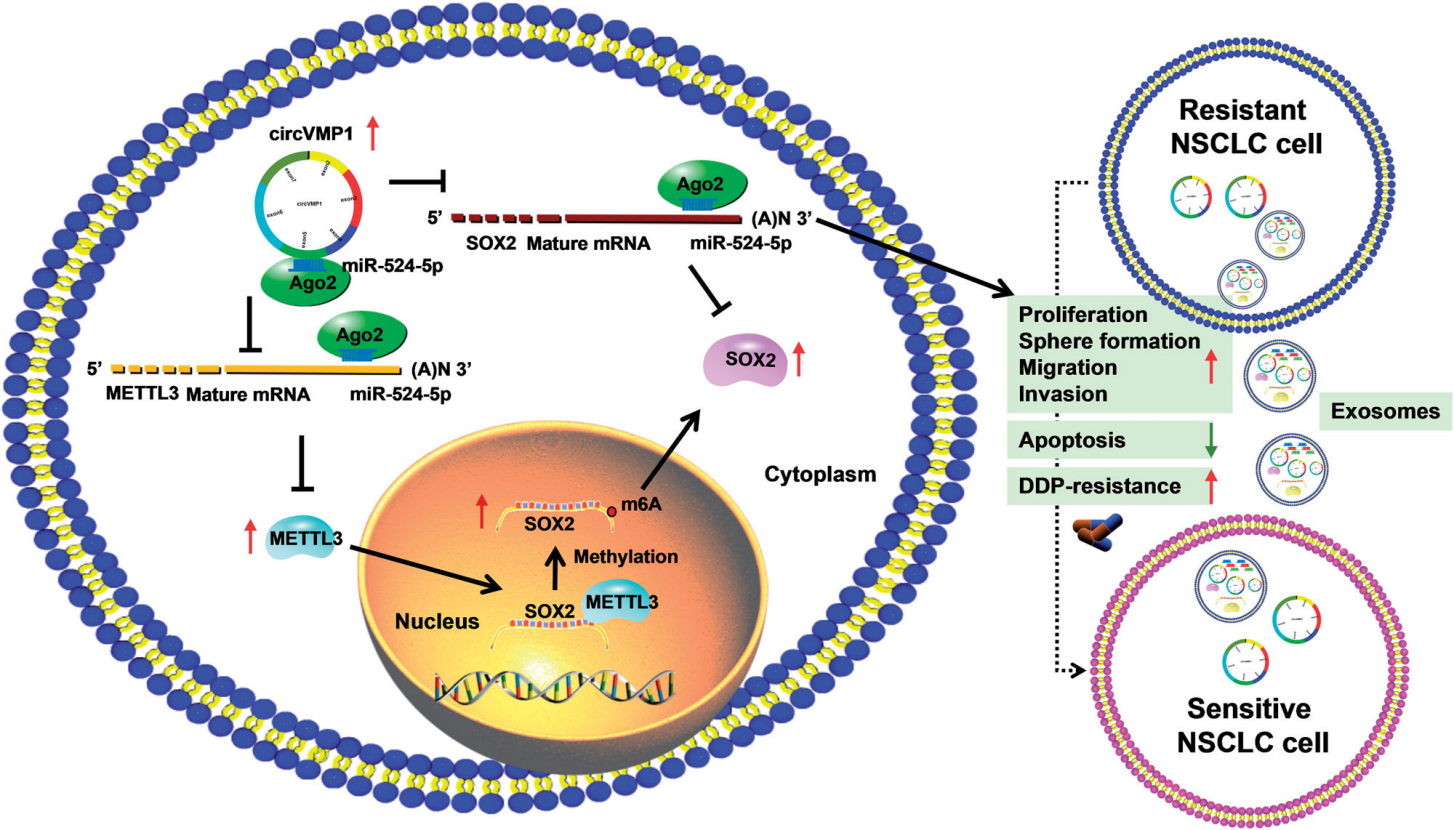
CircVMP1 promoted the proliferation, sphere formation, migration, invasion, and DDP resistance and suppressed the apoptosis of DDP-resistant NSCLC cells by regulating miR-524-5p-METTL3/SOX2 axis.

In conclusion, circVMP1 promoted the proliferation, sphere formation, migration, invasion, and DDP resistance and suppressed the apoptosis of DDP-resistant NSCLC cells by regulating miR-524-5p-METTL3/SOX2 axis (Figure 10). miR-524-5p could not only indirectly regulate SOX2 expression by interacting with its methyltransferase METTL3, but also could directly regulate SOX2 expression by binding to its 3’UTR (Figure 10). DDP-resistant NSCLC cells-derived exosomal circVMP1 conferred the malignant potential and DDP resistance to DDP-sensitive cells (Figure 10). Exosomal circVMP1 in the serum samples of patients might be a novel bio-marker for NSCLC therapy.

## Supplementary Material

Supplemental Material
