# Supplementary material for: Exosome-transmitted circVMP1 facilitates the progression and cisplatin resistance of non-small cell lung cancer by targeting miR-524-5p-METTL3/SOX2 axis
Source: Drug Deliv. 2022 Apr 25;29(1):1257–71. doi: 10.1080/10717544.2022.2057617 (PMC9045767; doi:10.1080/10717544.2022.2057617)

Fig2G 1 sh-NC 2 sh-circVMP1

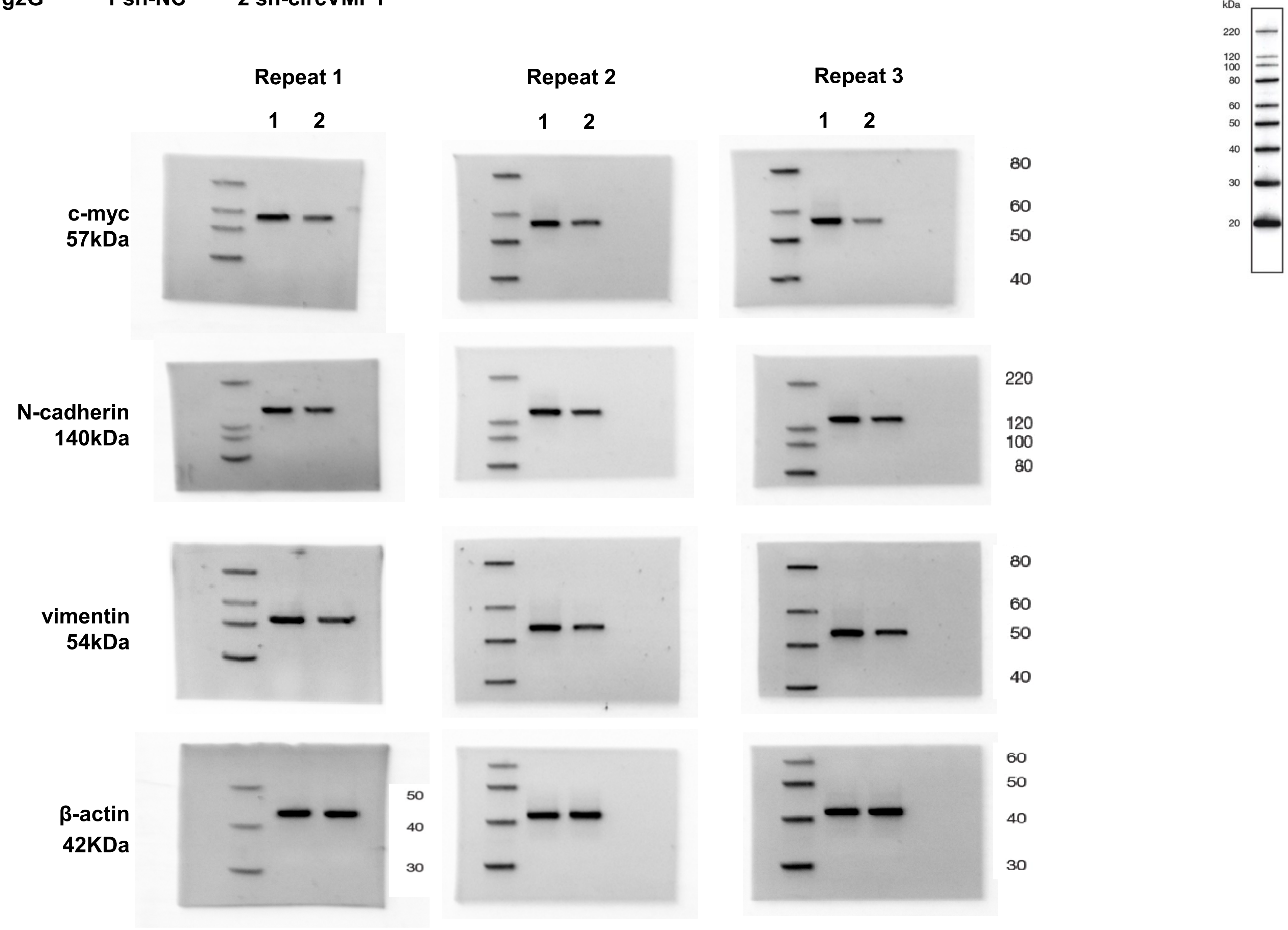

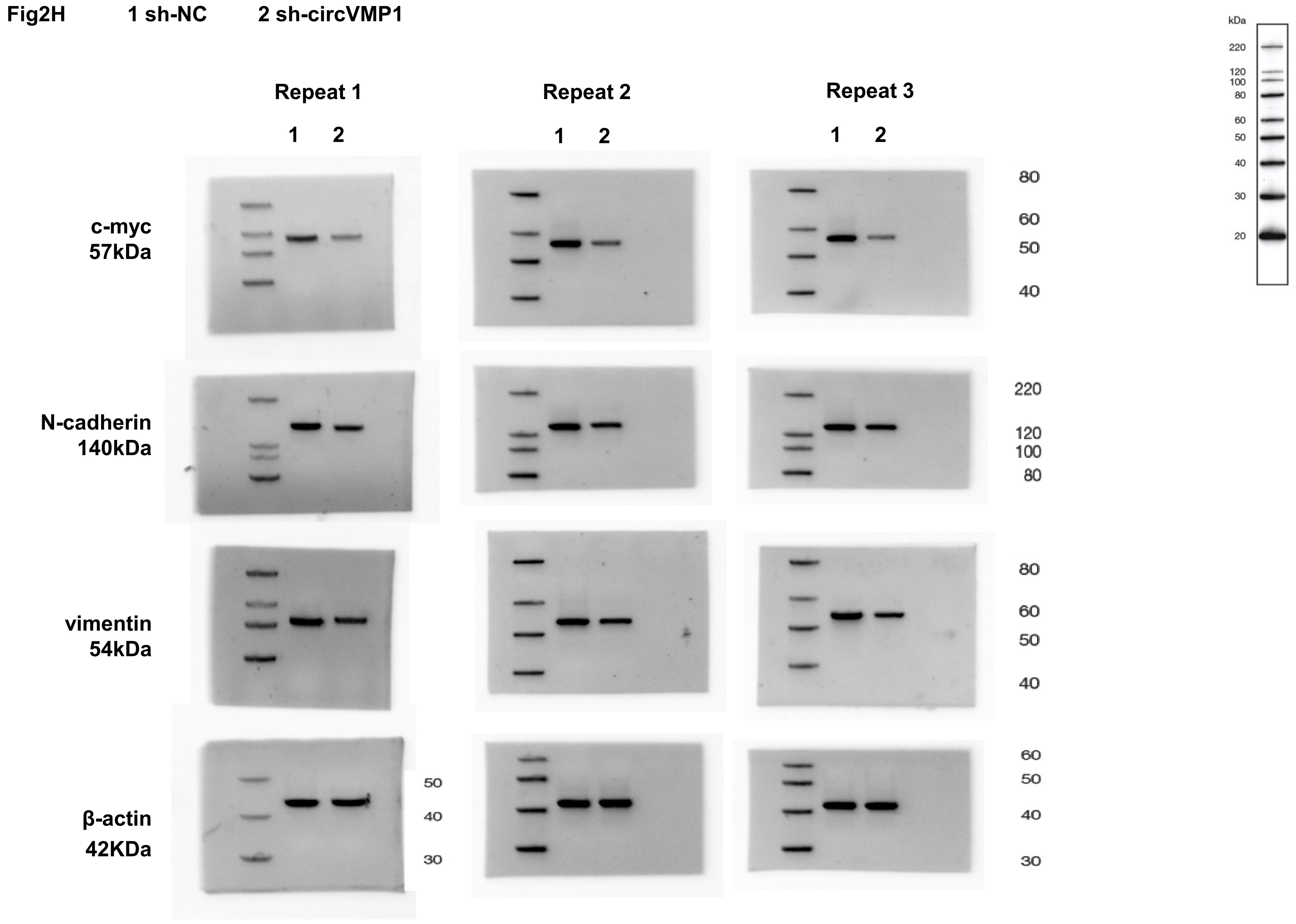

Fig2H
1 sh-NC
2 sh-circVMP1
Repeat 1
Repeat 2
Repeat 3
1
2
c-myc
57kDa
N-cadherin
140kDa
vimentin
54kDa
β-actin
42KDa
80
60
50
40
220
120
100
30
kDa
20

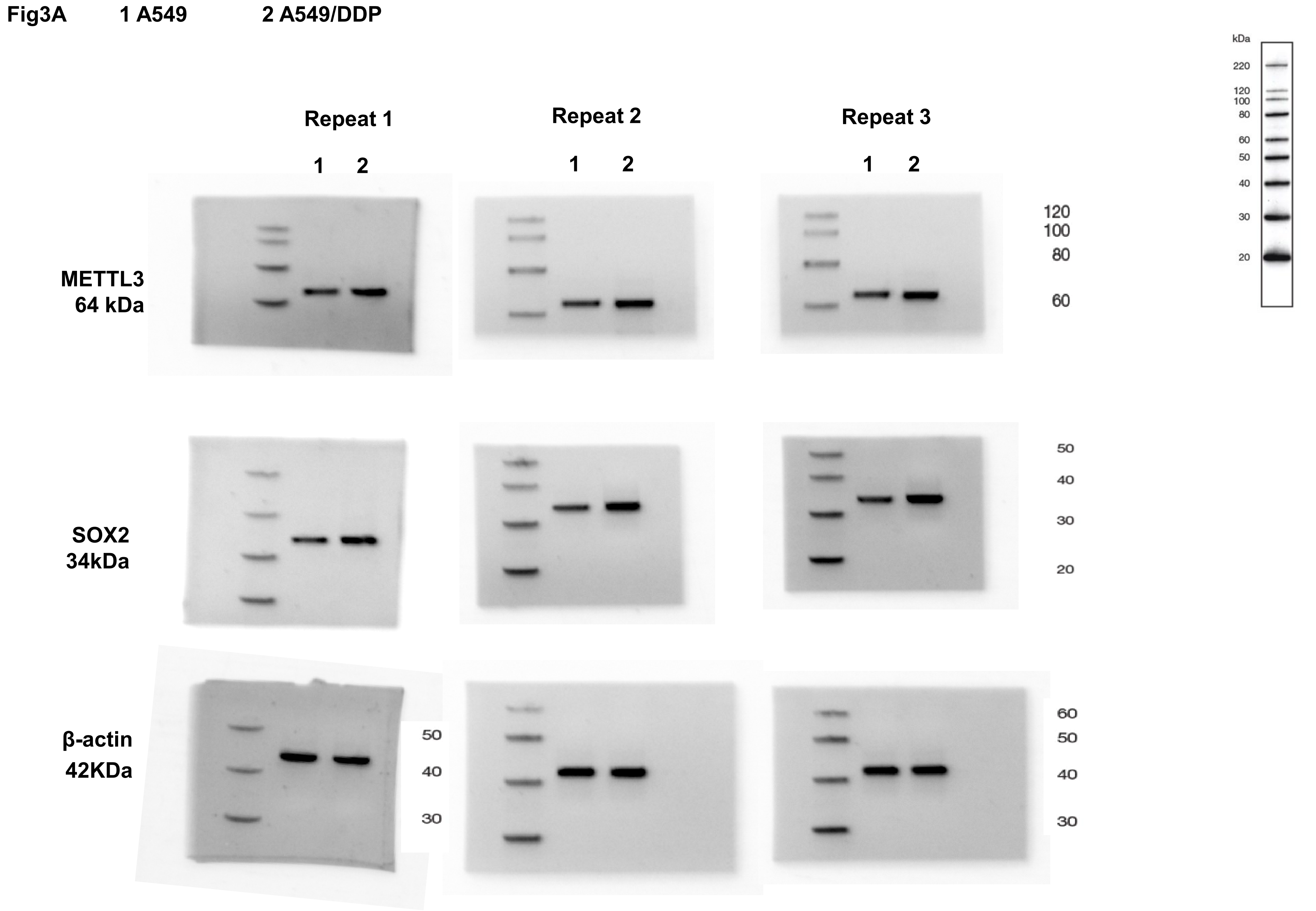
Fig3A
1 A549
2 A549/DDP
Repeat 1
Repeat 2
Repeat 3
1
2
METTL3
64 kDa
SOX2
34kDa
β-actin
42KDa
120
100
80
60
50
40
30
20
kDa
220

Fig3B 1 H1299 2 H1299/DDP

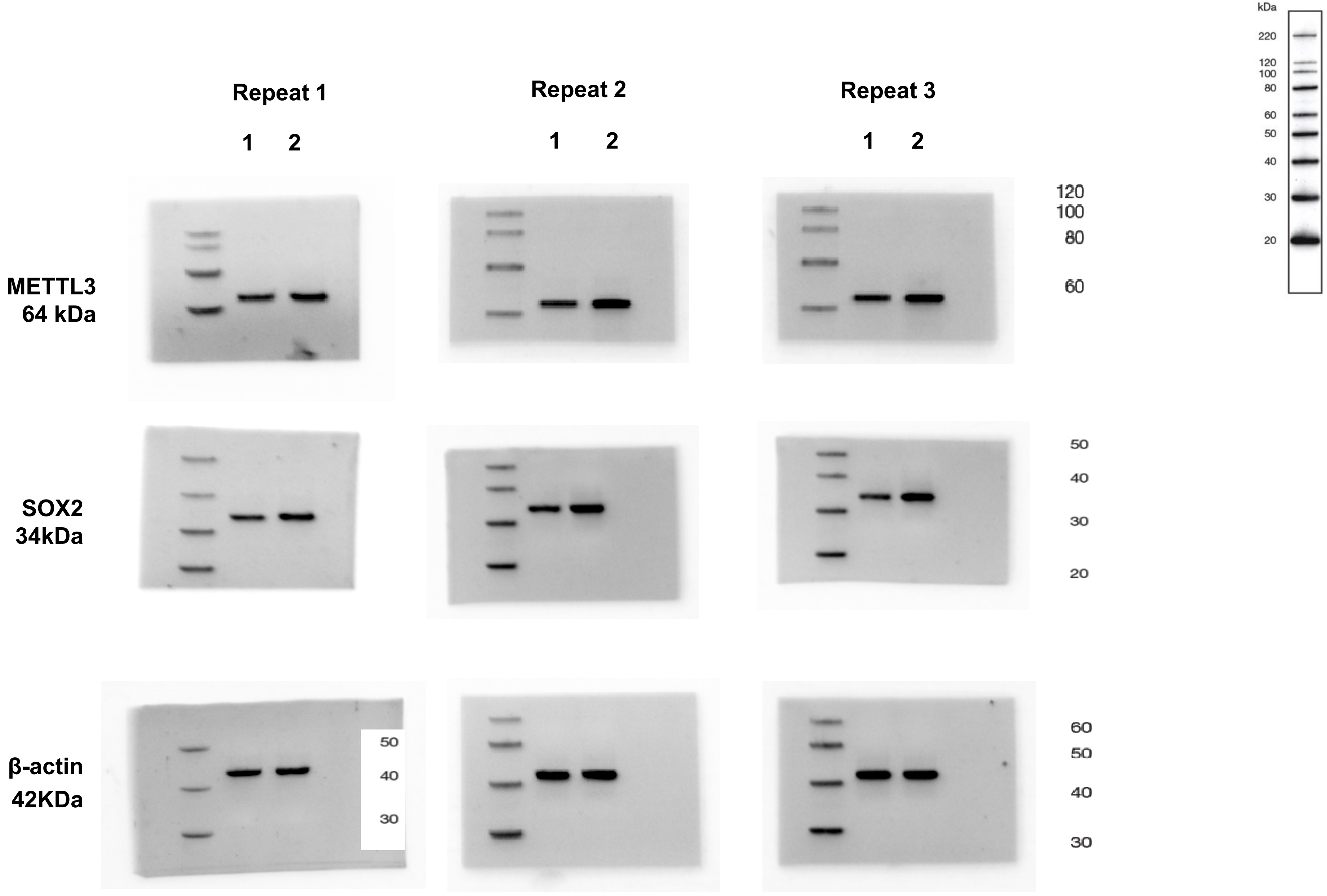

Fig3E 1 sh-NC 2 sh-METTL3

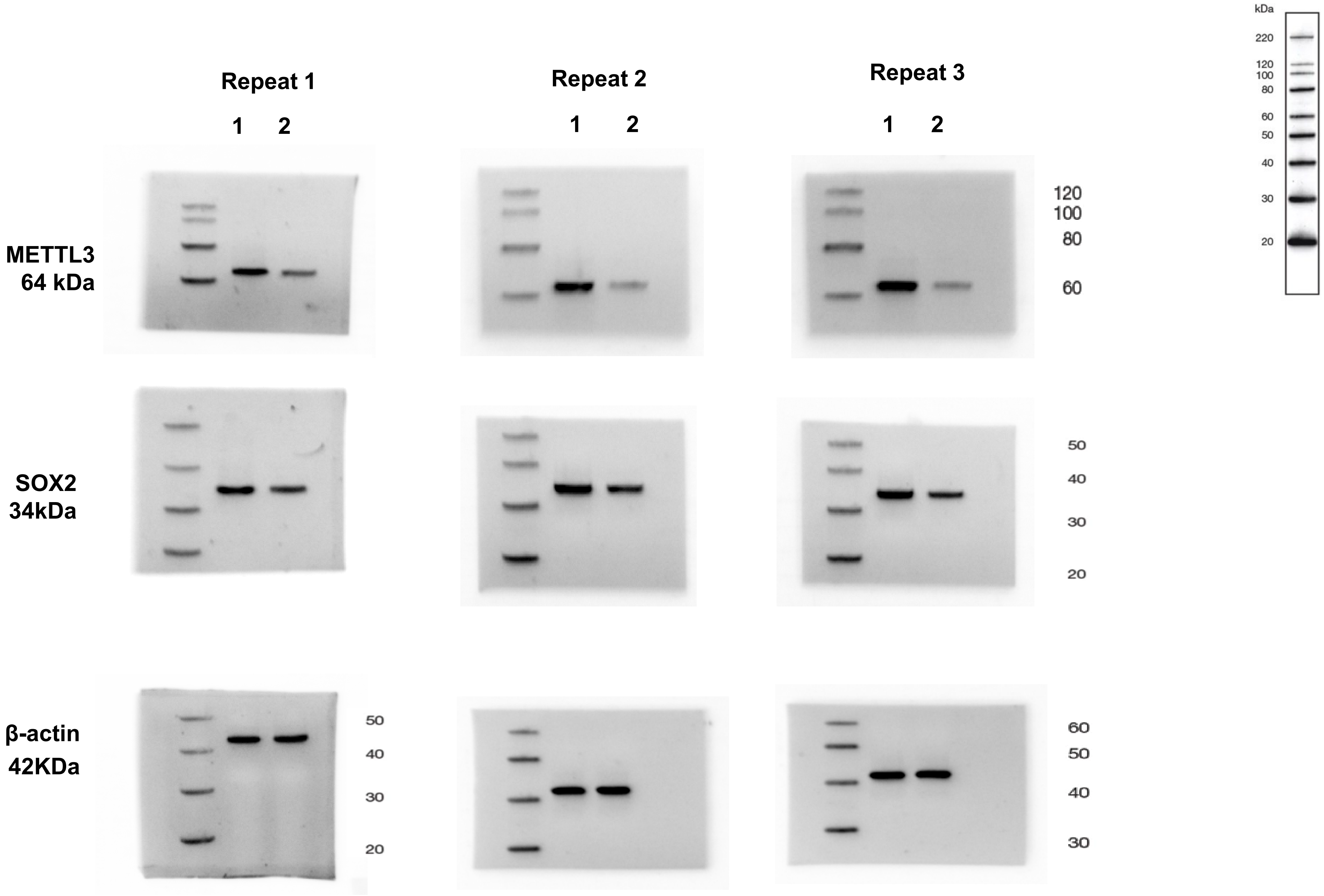

Fig3F 1 sh-NC 2 sh-METTL3

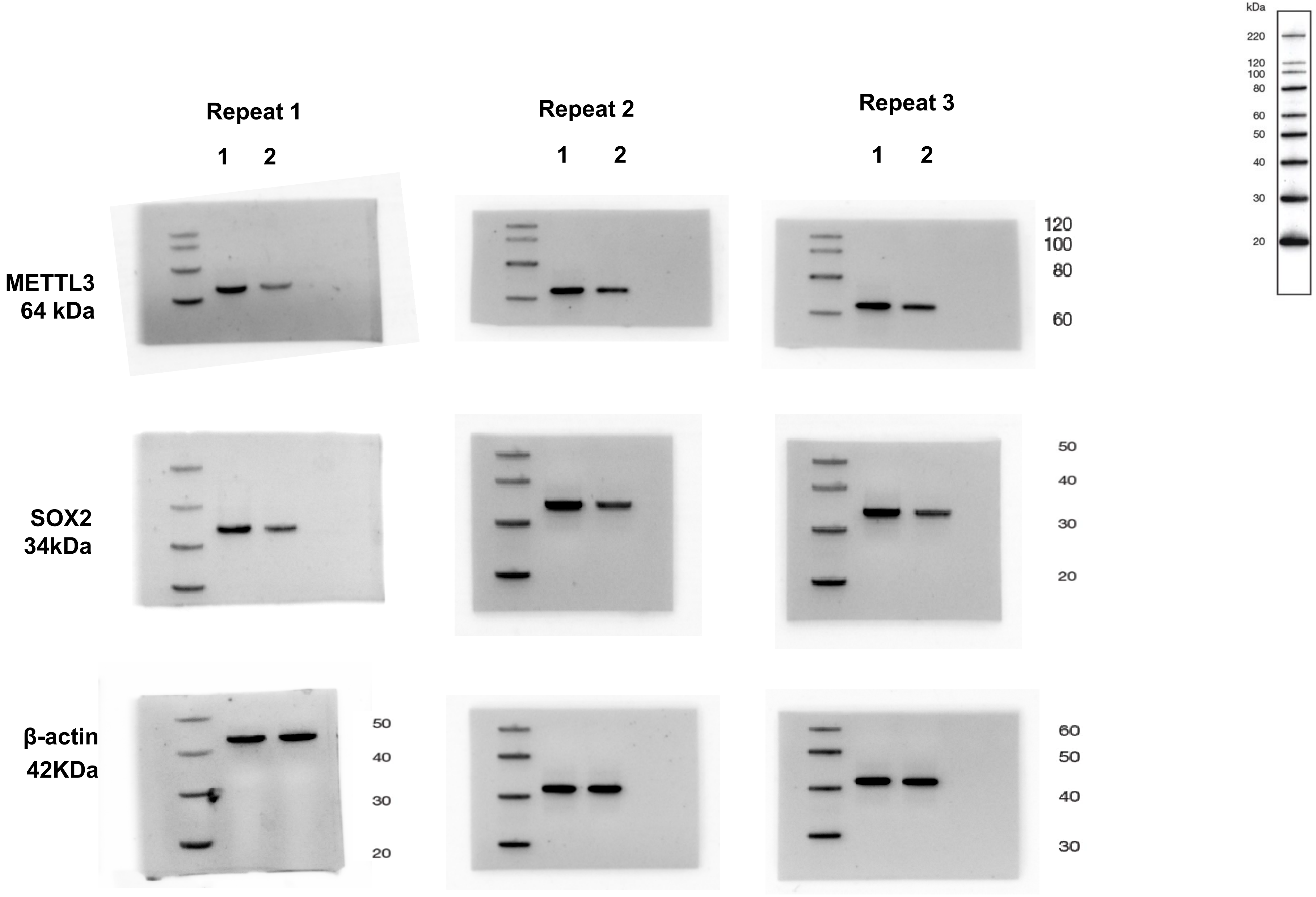

Fig3J 1 sh-NC 2 sh-circVMP1

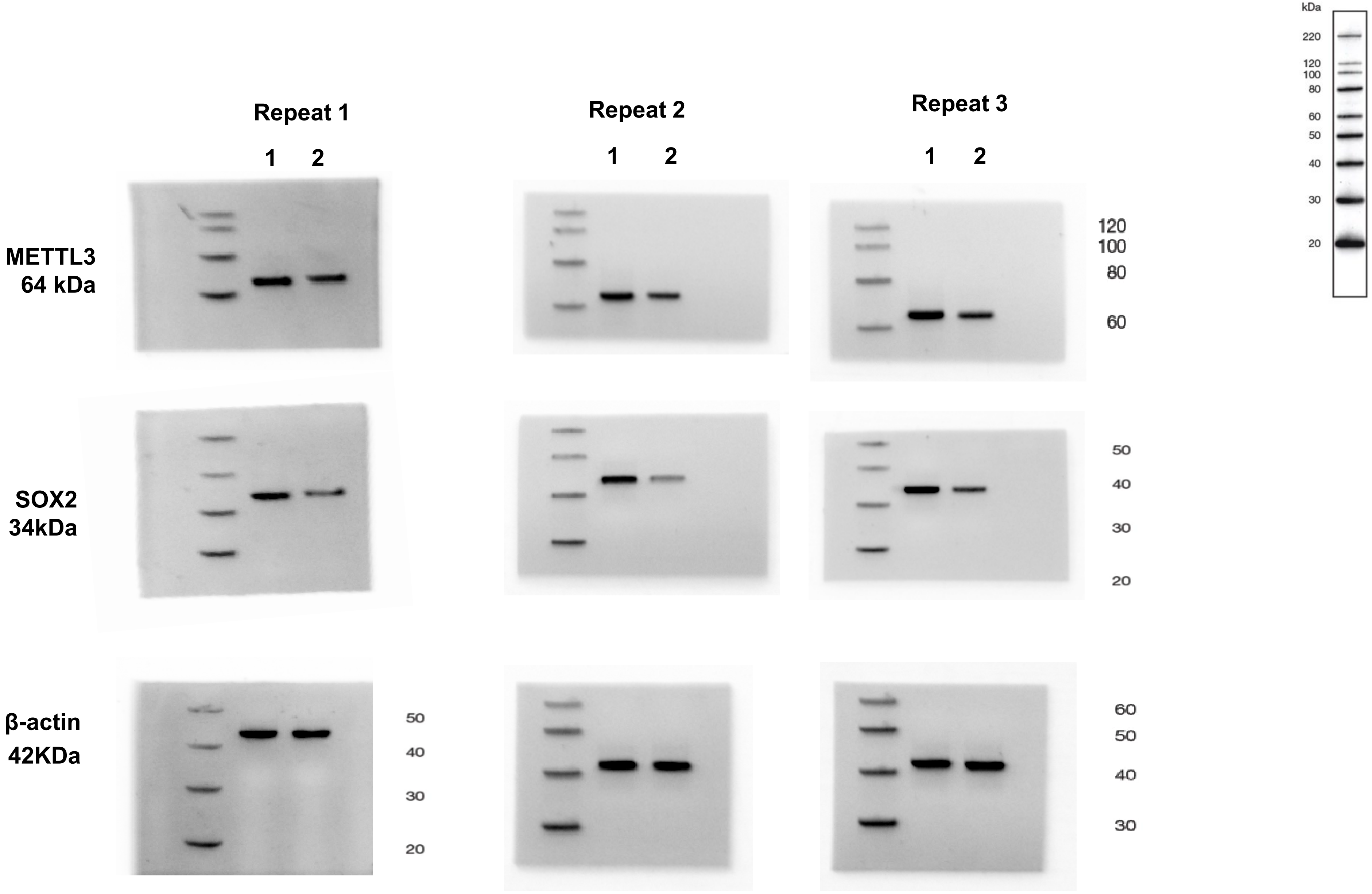

Fig3K 1 sh-NC 2 sh-circVMP1

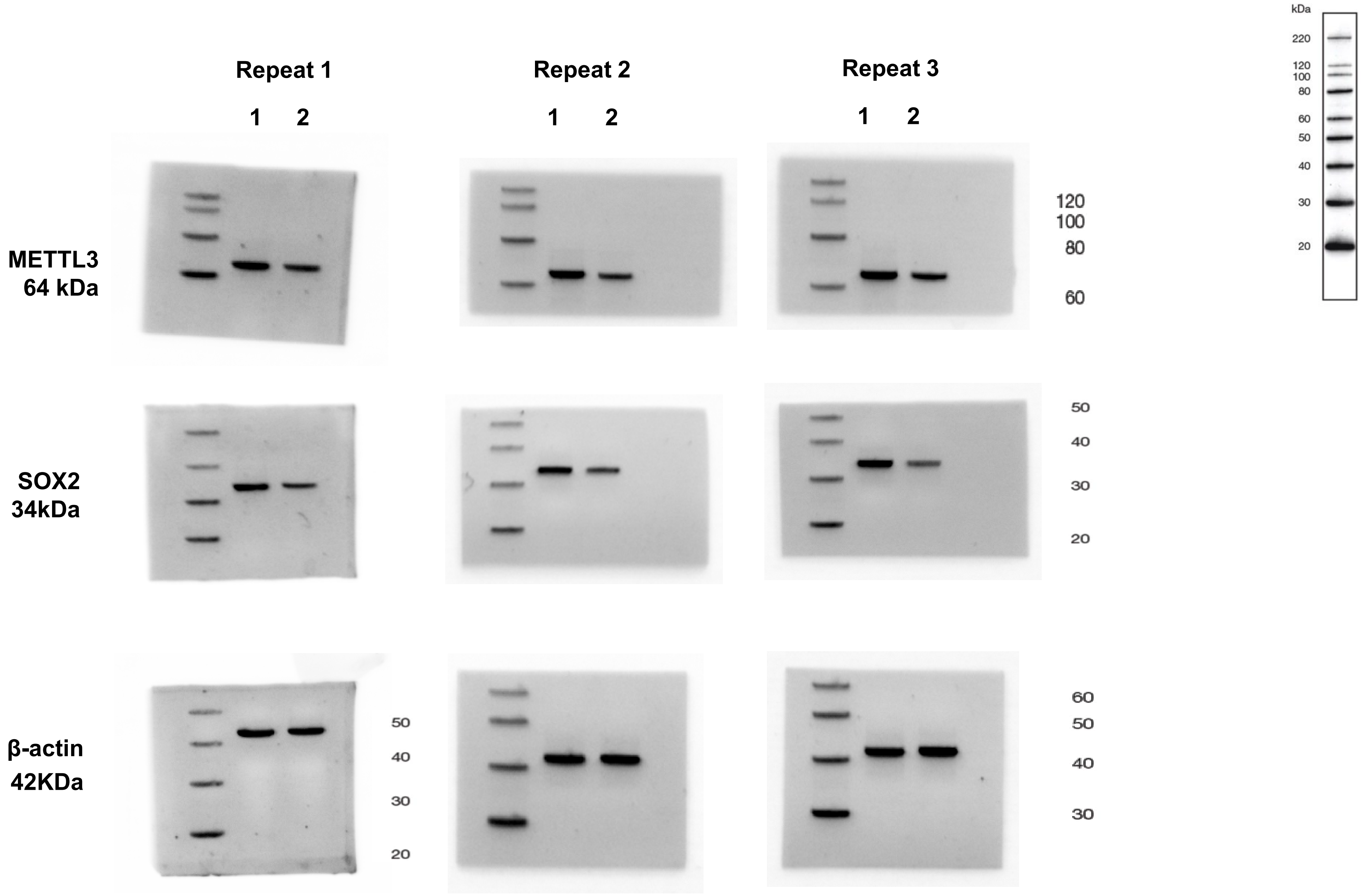

Fig4L 1 miR-NC 2 miR-524-5p

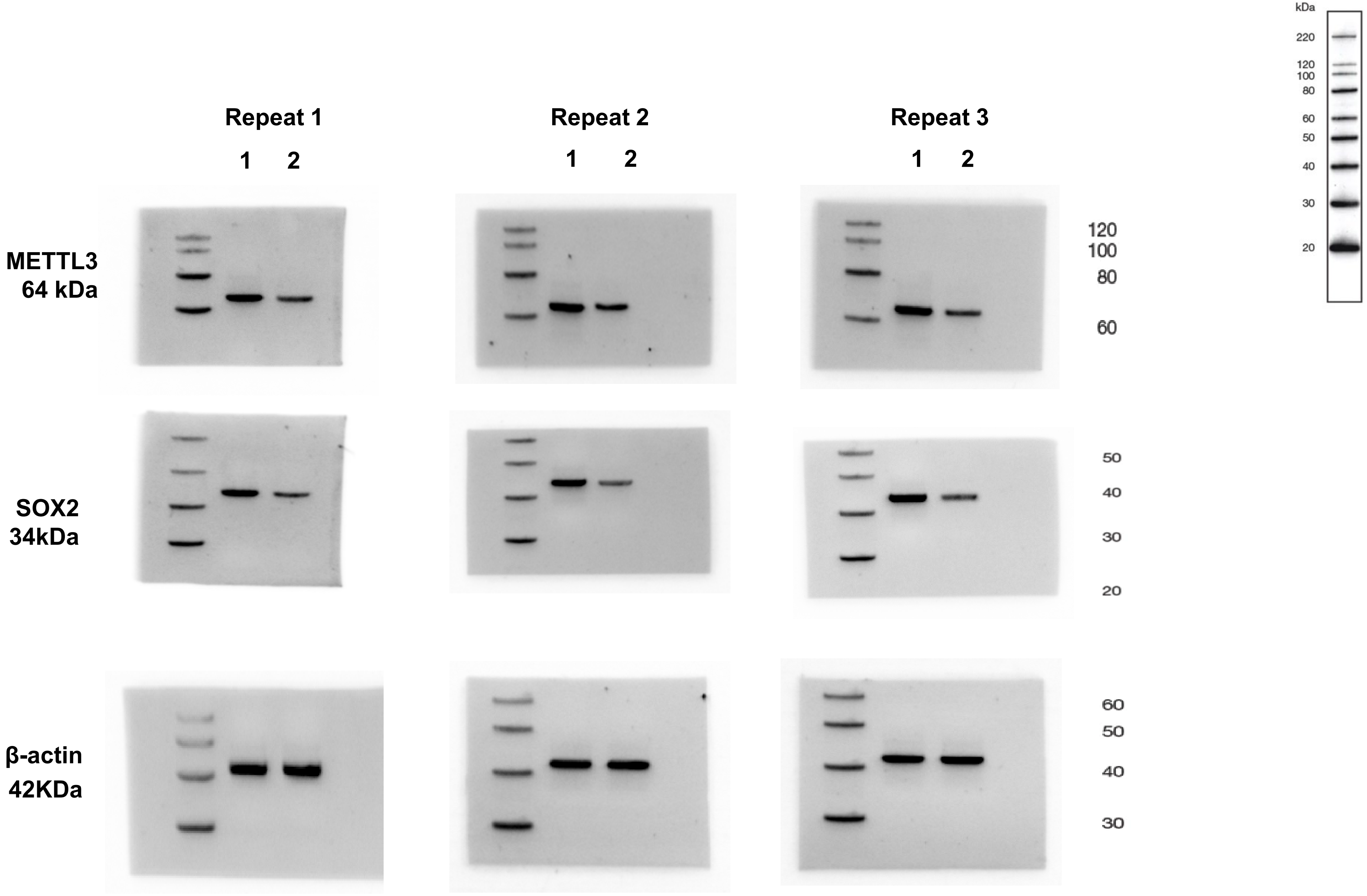

Fig4M 1 miR-NC 2 miR-524-5p

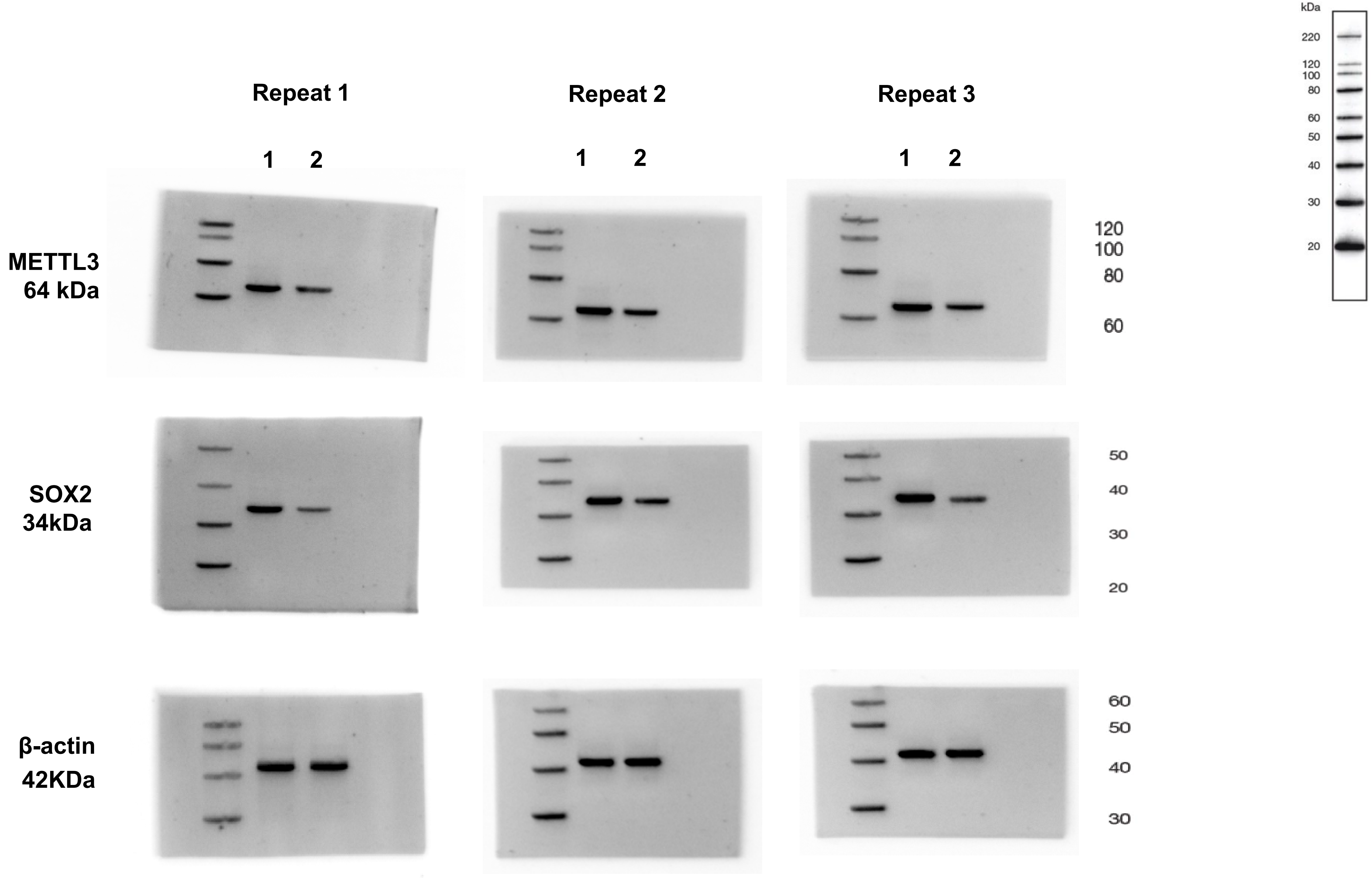

Fig4O 1 sh-NC+anti-NC 2 sh-circVMP1+anti-NC 2 sh-circVMP1+anti-miR-524-5p

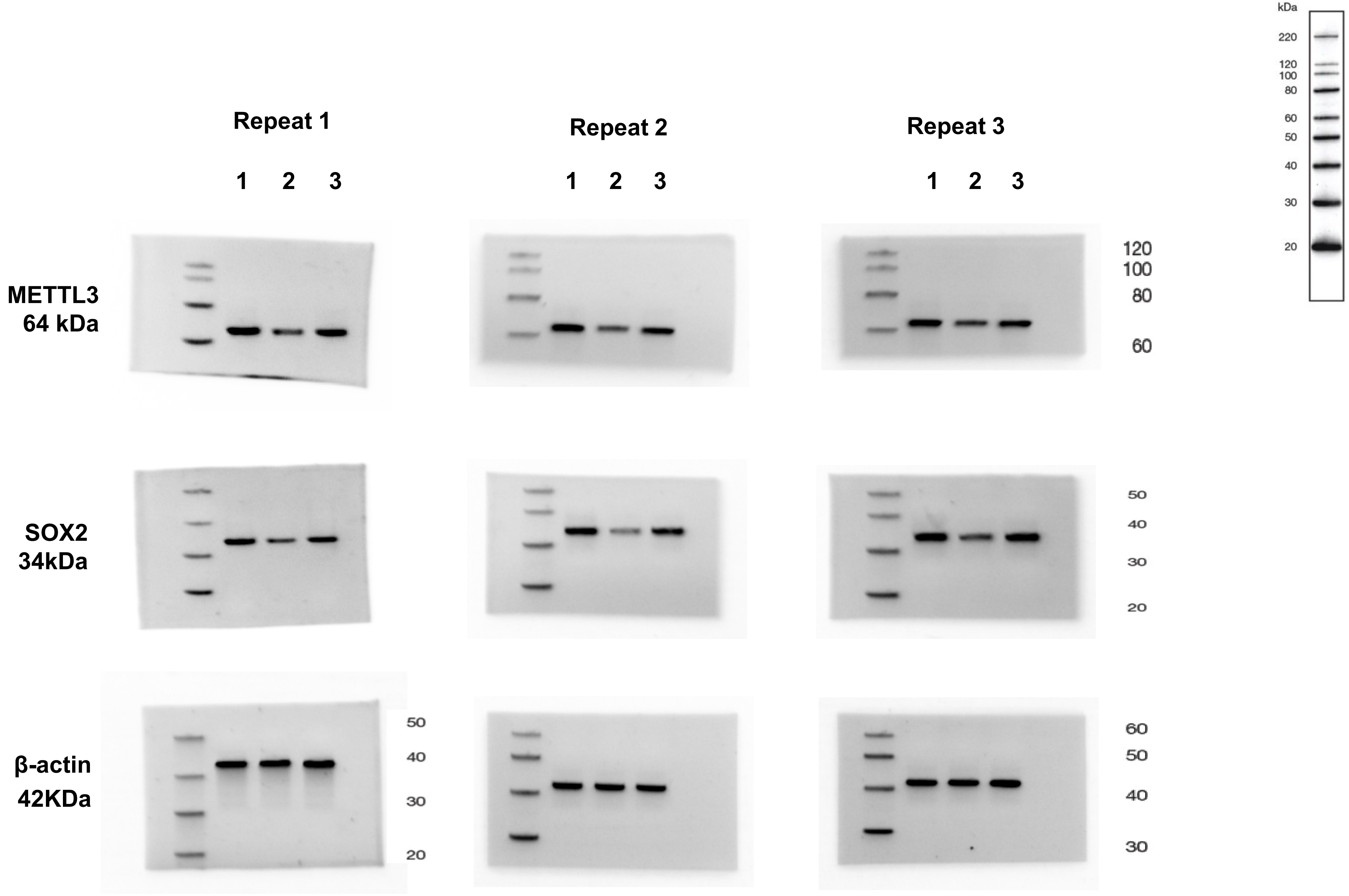

Fig4P 1 sh-NC+anti-NC 2 sh-circVMP1+anti-NC 2 sh-circVMP1+anti-miR-524-5p

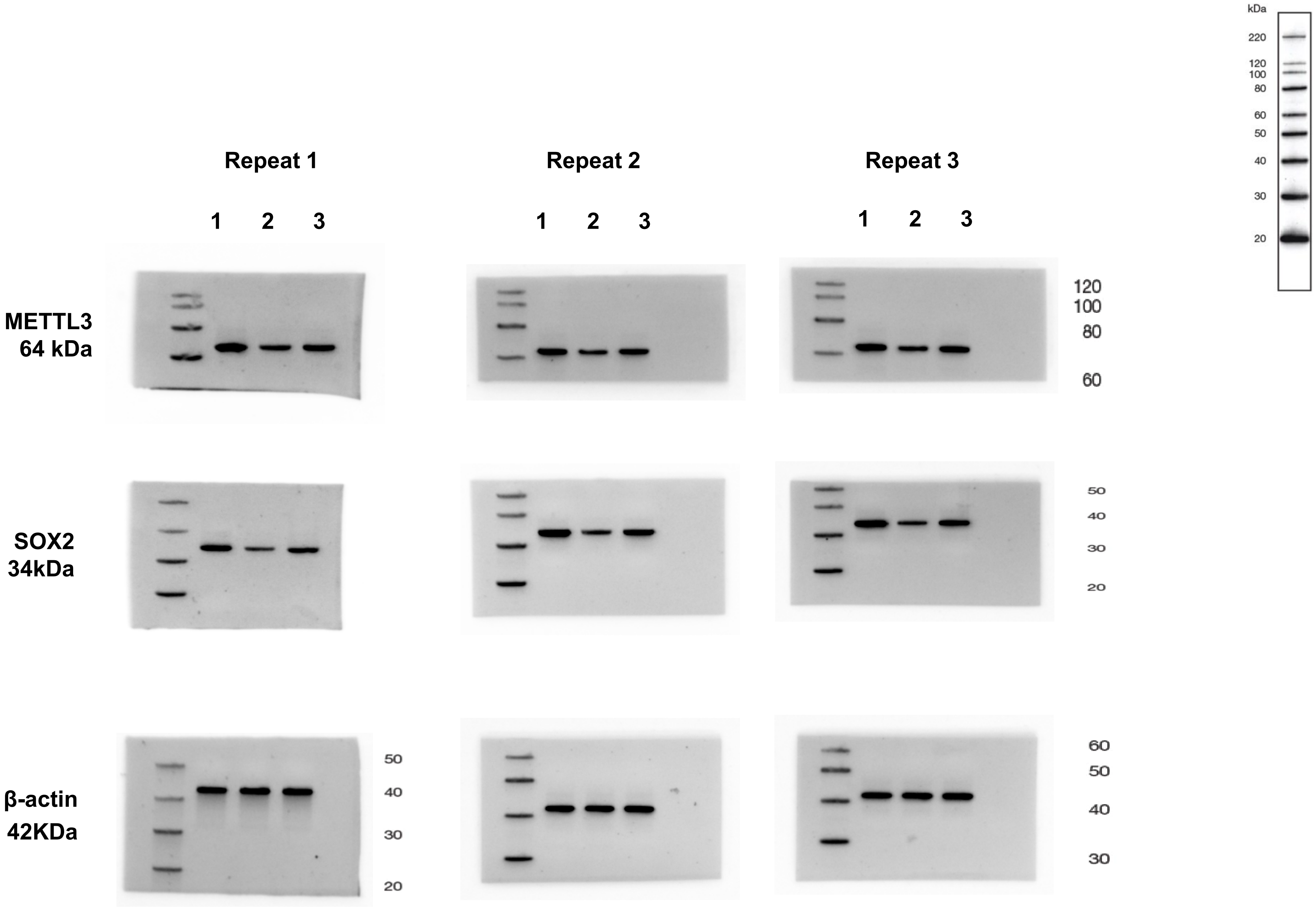

Fig5G 1 sh-NC+anti-NC 2 sh-circVMP1+anti-NC 2 sh-circVMP1+anti-miR-524-5p

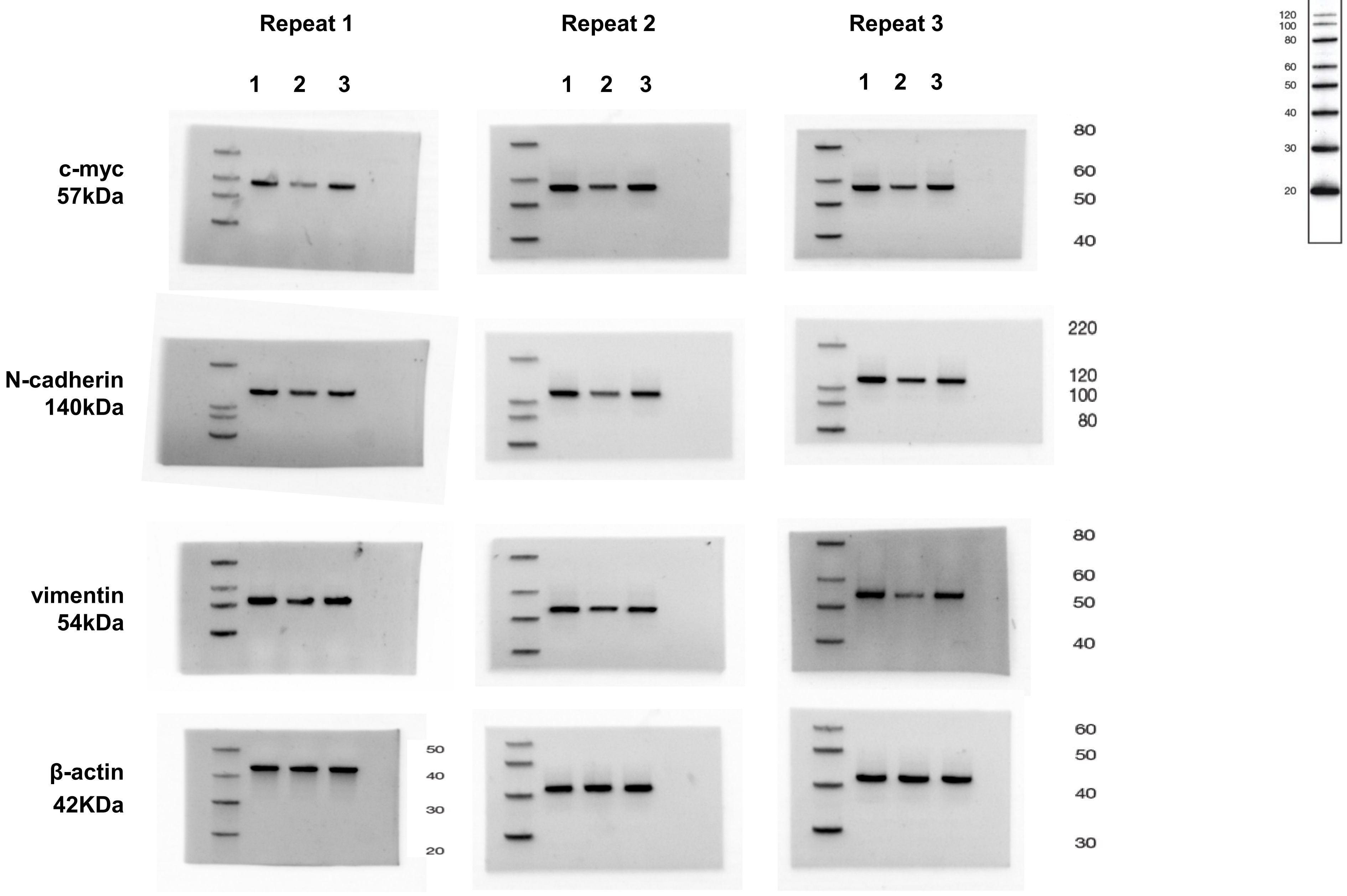

Fig5H 1 sh-NC+anti-NC 2 sh-circVMP1+anti-NC 2 sh-circVMP1+anti-miR-524-5p

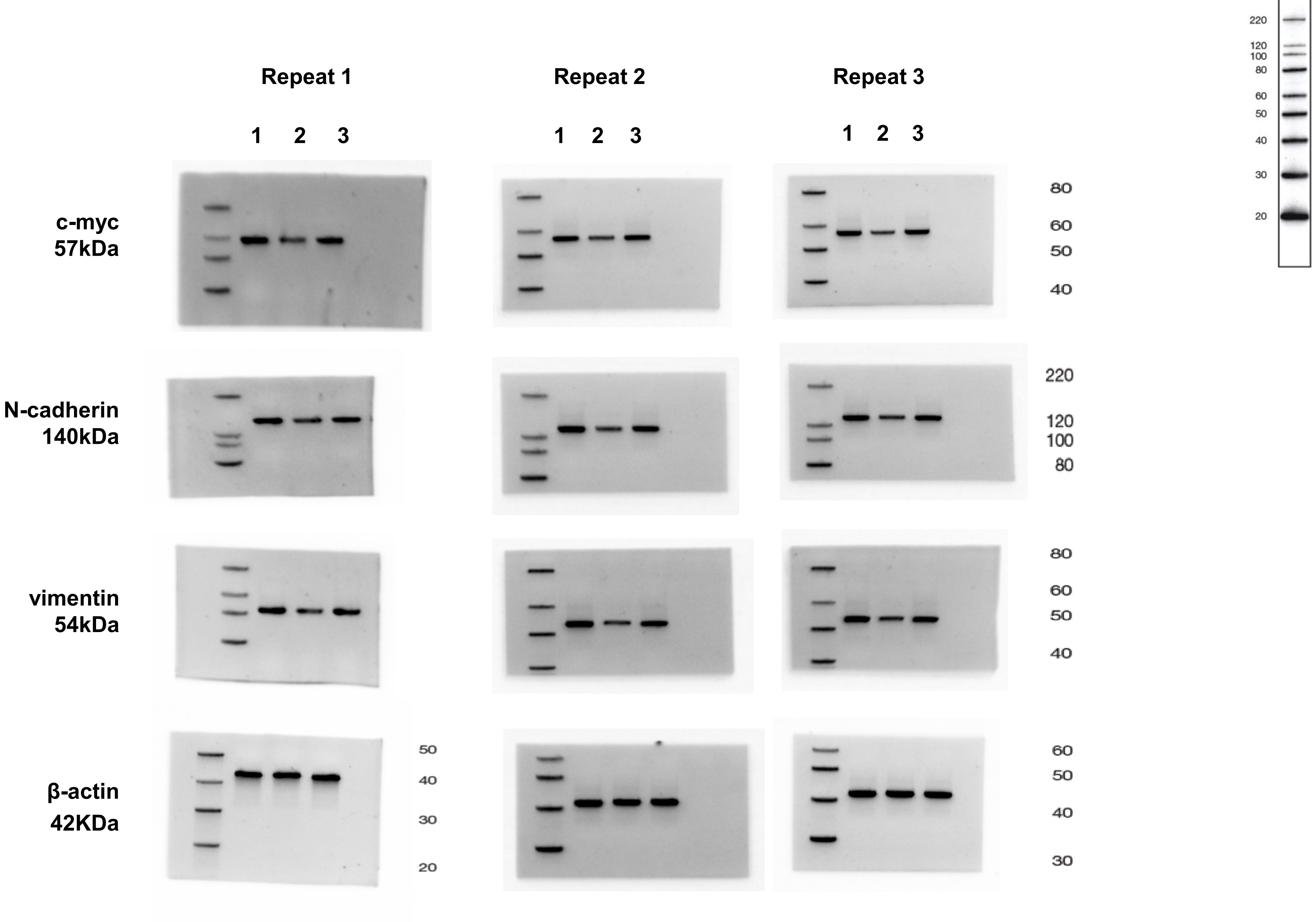

Fig6C 1 A549 2 A549/DDP 3 H1299 4 H1299/DDP

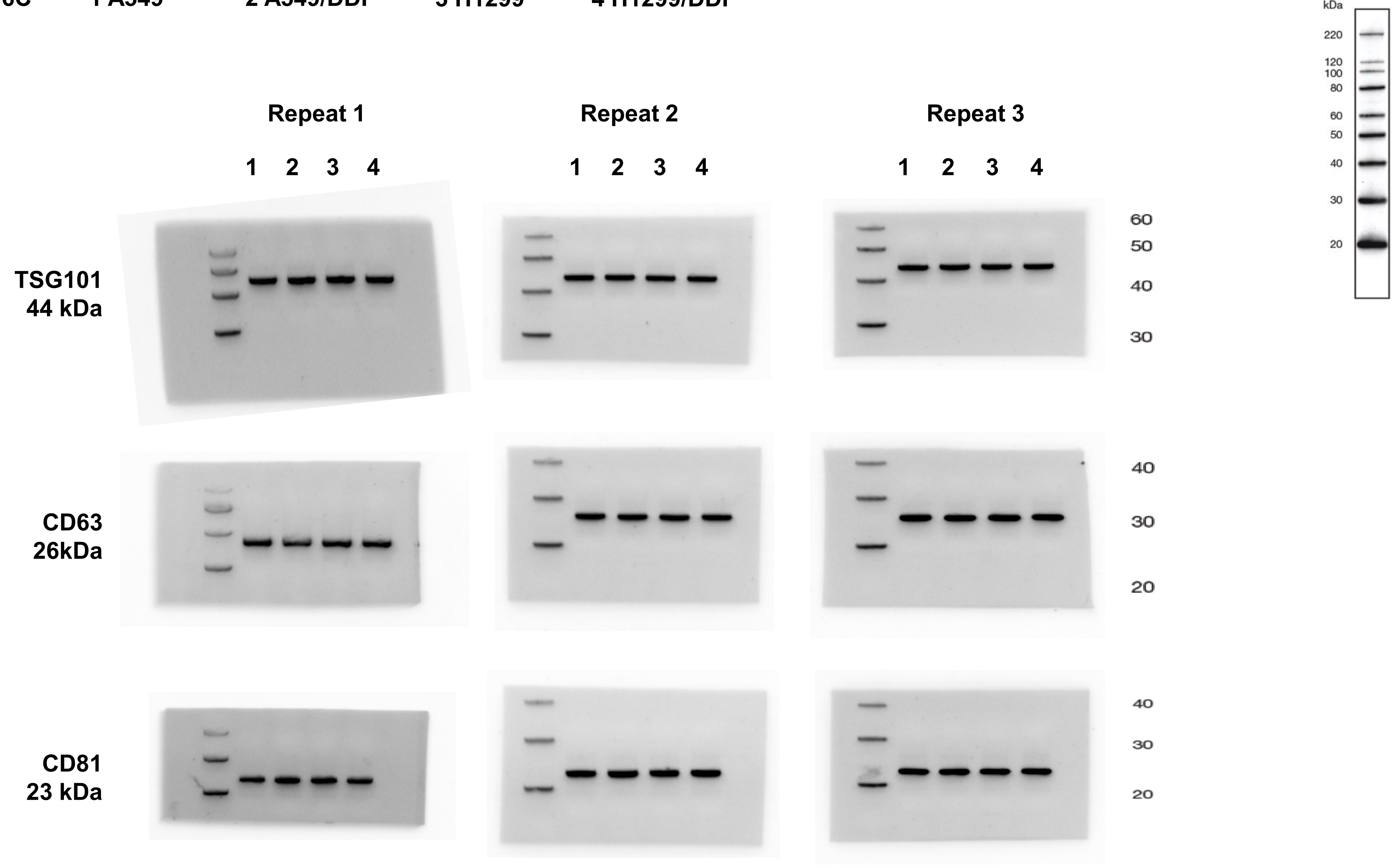

Fig7A 1 PBS 2 A549/DDP-sh-NC exo 3 A549/DDP-sh-circVMP1 exo

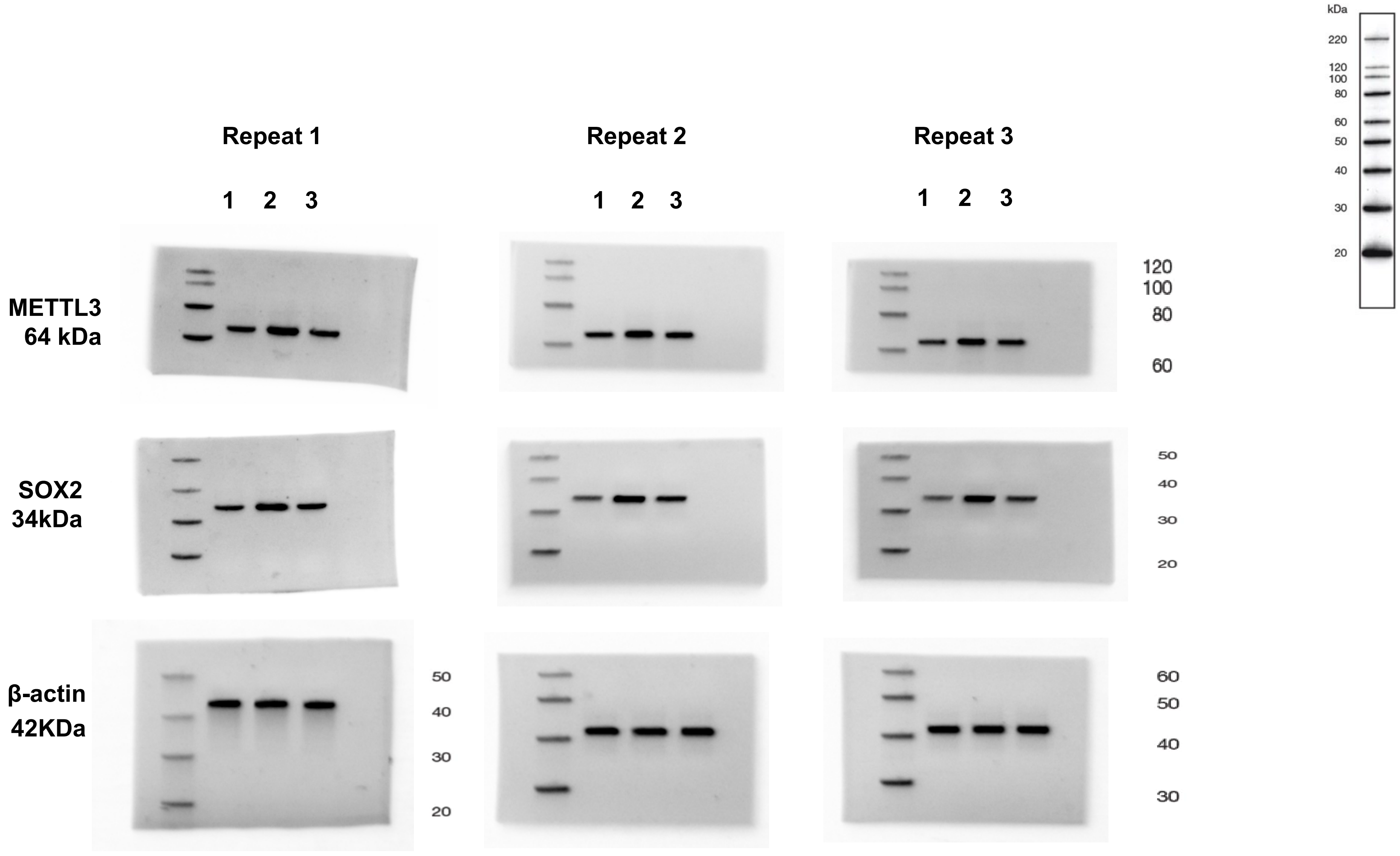

Fig7B 1 PBS 2 H1299/DDP-sh-NC exo 3 H1299/DDP-sh-circVMP1 exo

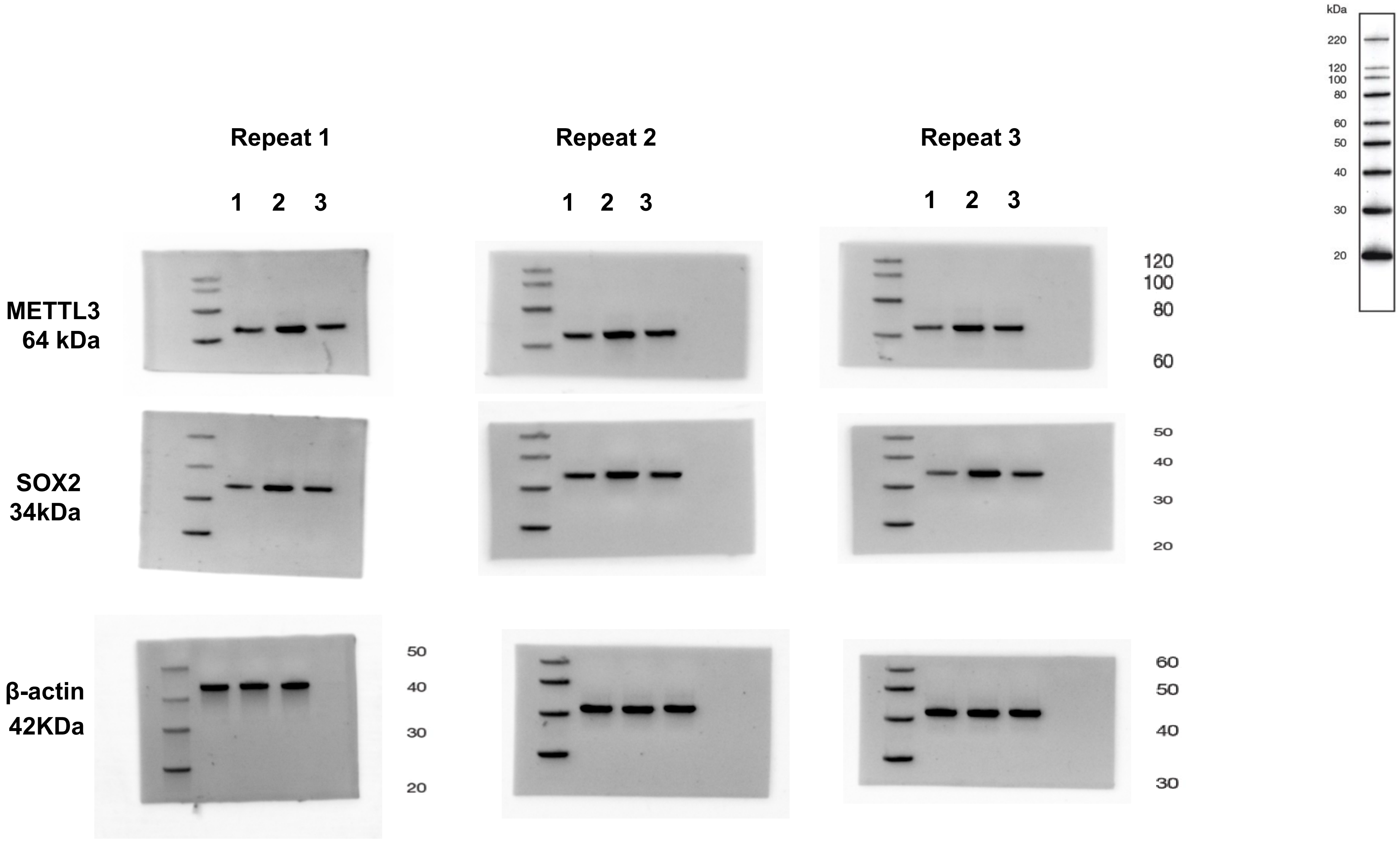

Fig7T 1 PBS 2 A549/DDP-sh-NC exo 3 A549/DDP-sh-circVMP1 exo

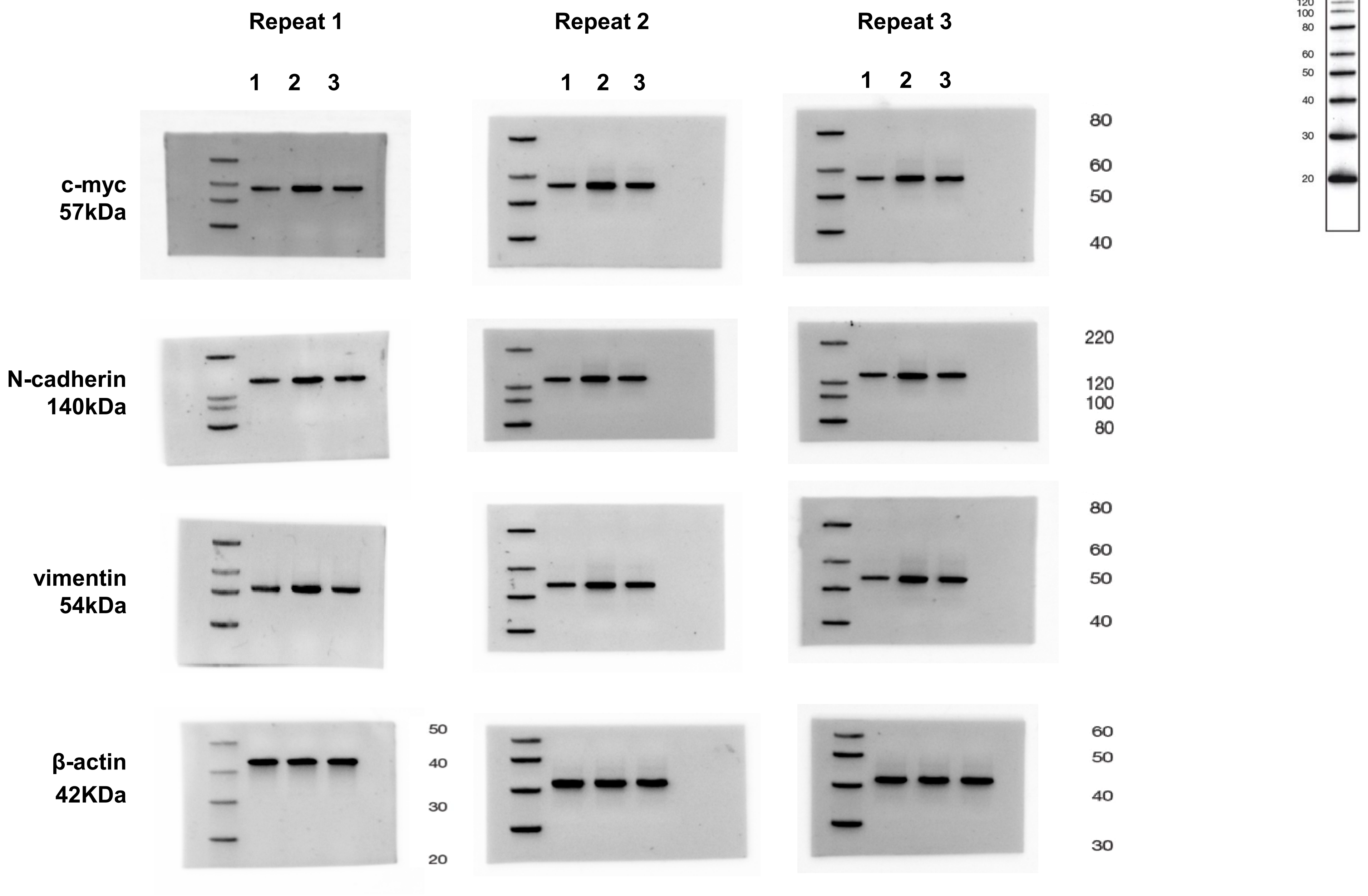

Fig7U 1 PBS 2 H1299/DDP-sh-NC exo 3 H1299/DDP-sh-circVMP1 exo

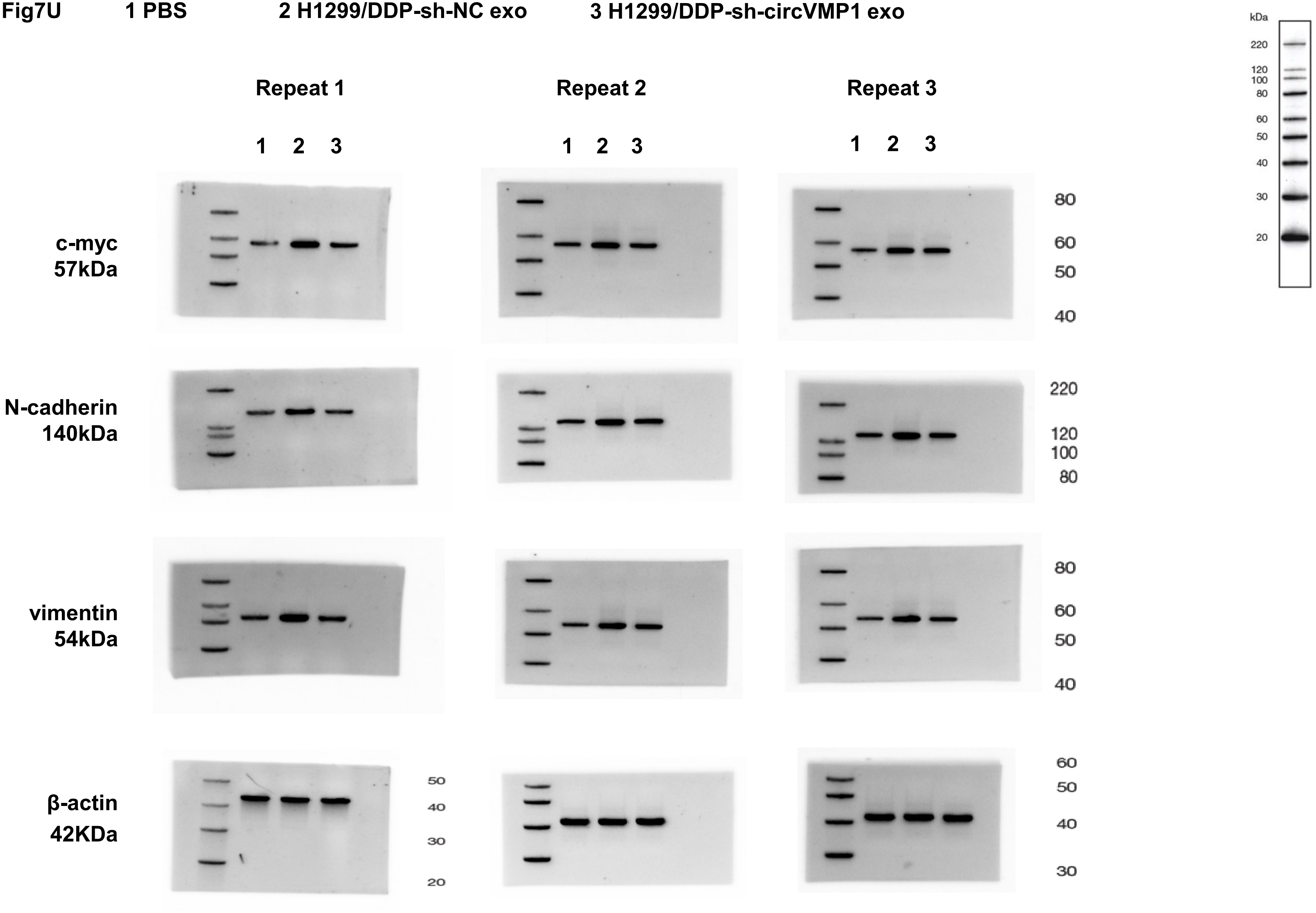

Fig8E 1 PBS 2 A549/DDP-sh-NC exo 3 A549/DDP-sh-circVMP1 exo

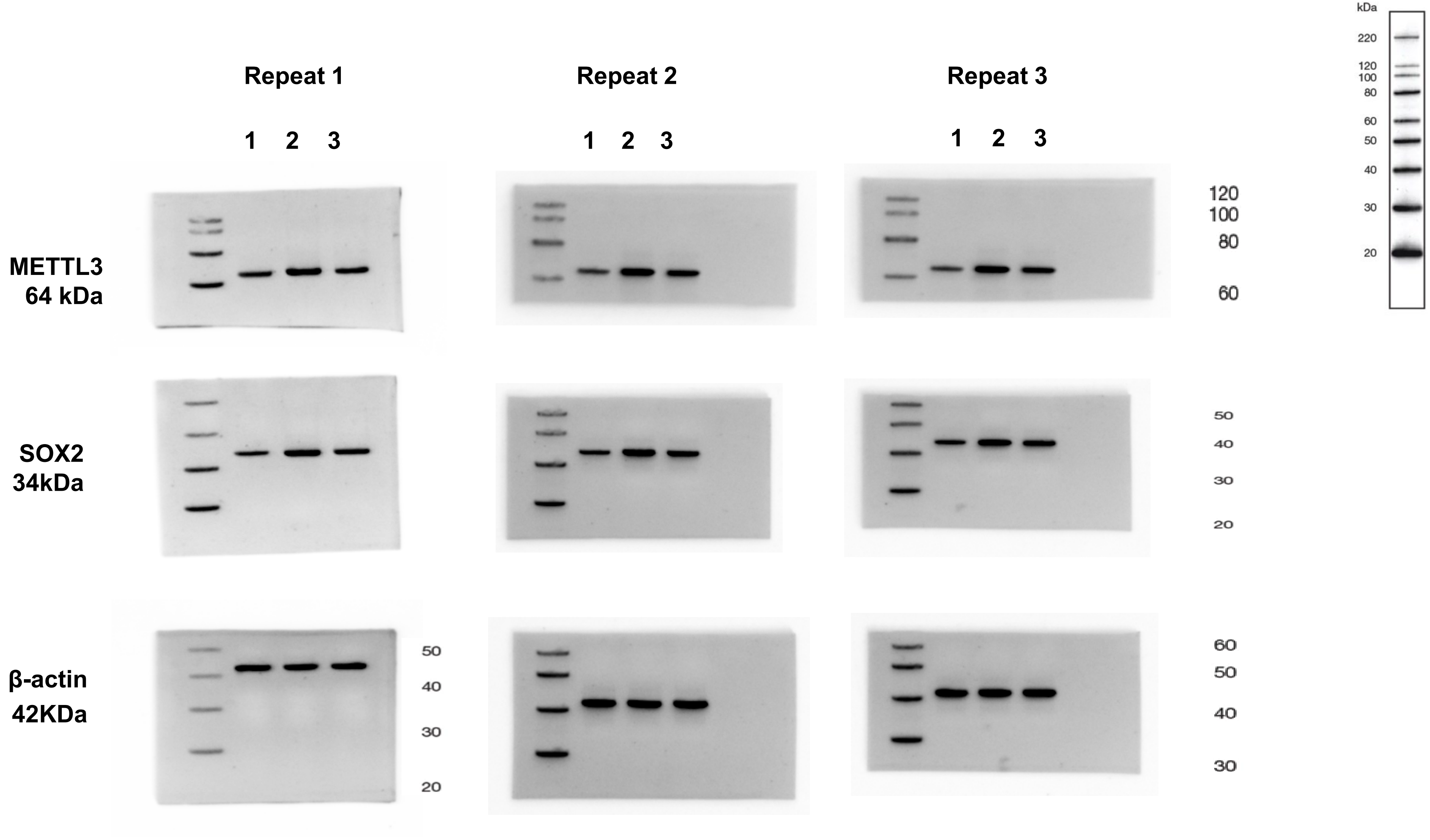

Fig9C 1 DDP-sensitive 2 DDP-resistant

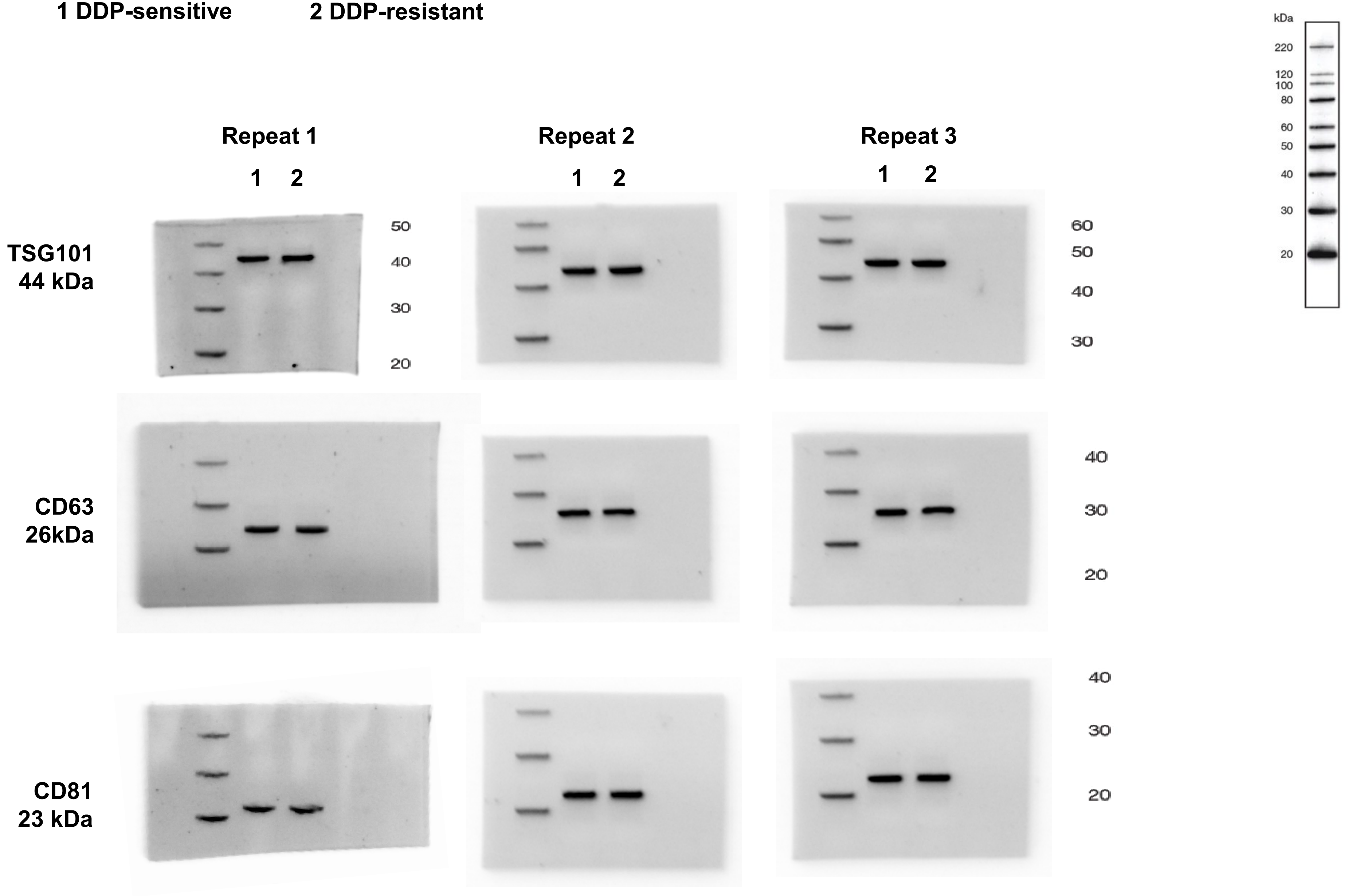

Fig1H

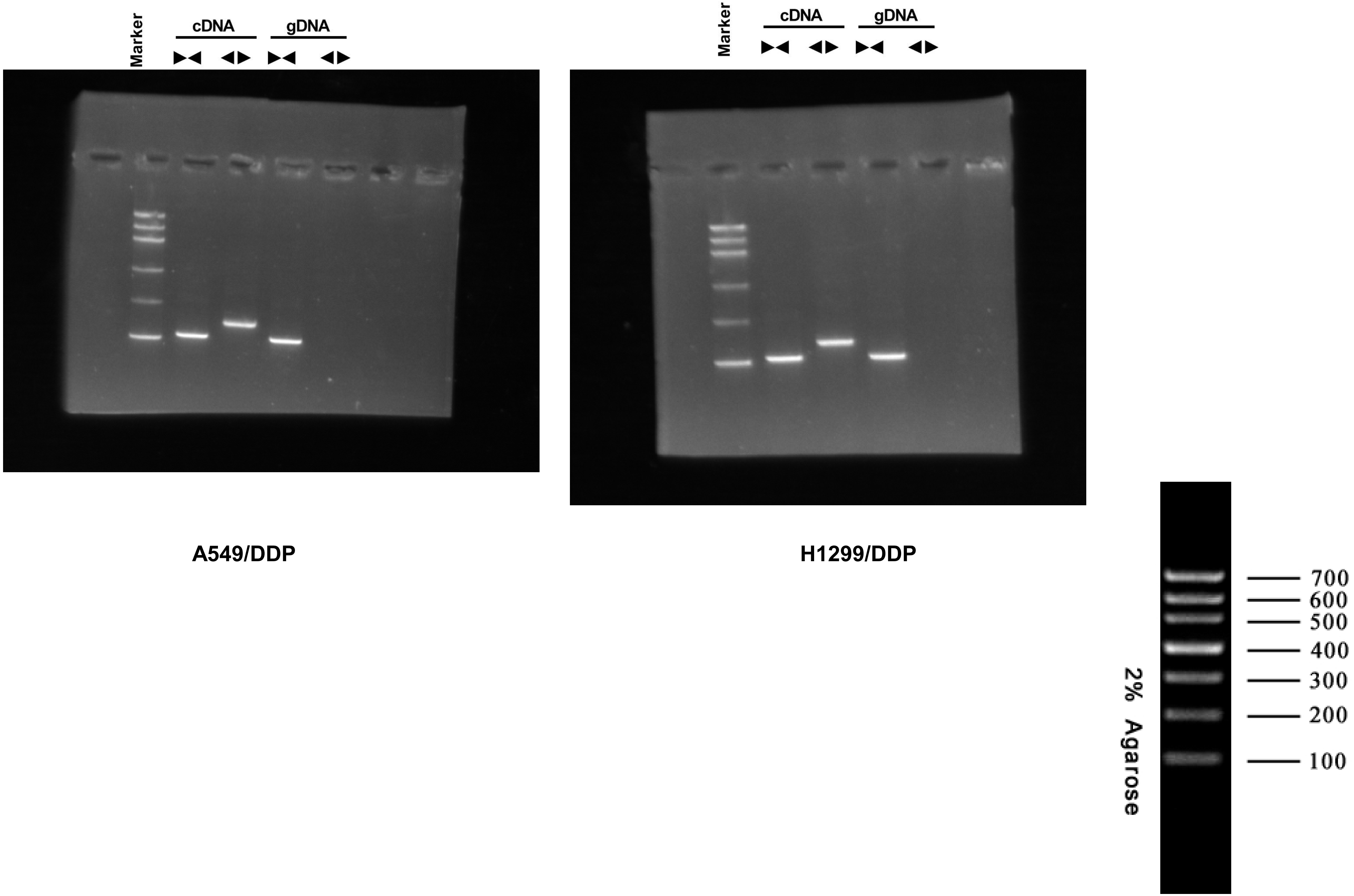
Marker
cDNA
gDNA
A549/DDP
H1299/DDP
700
600
500
400
300
200
100
2% Agarose

Fig2A

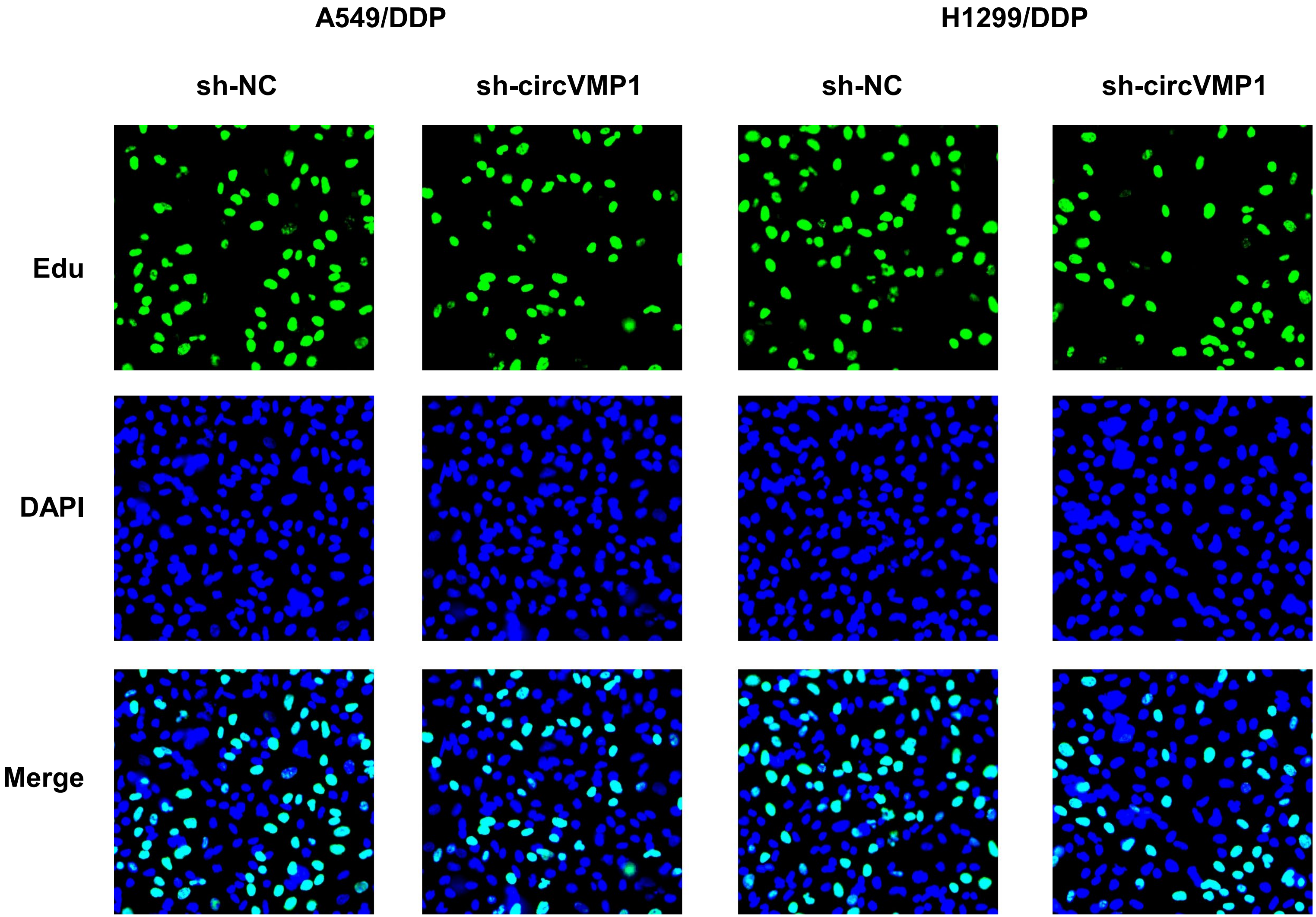
A549/DDP
H1299/DDP
sh-NC
sh-circVMP1
sh-NC
sh-circVMP1
Edu
DAPI
Merge

Fig2B

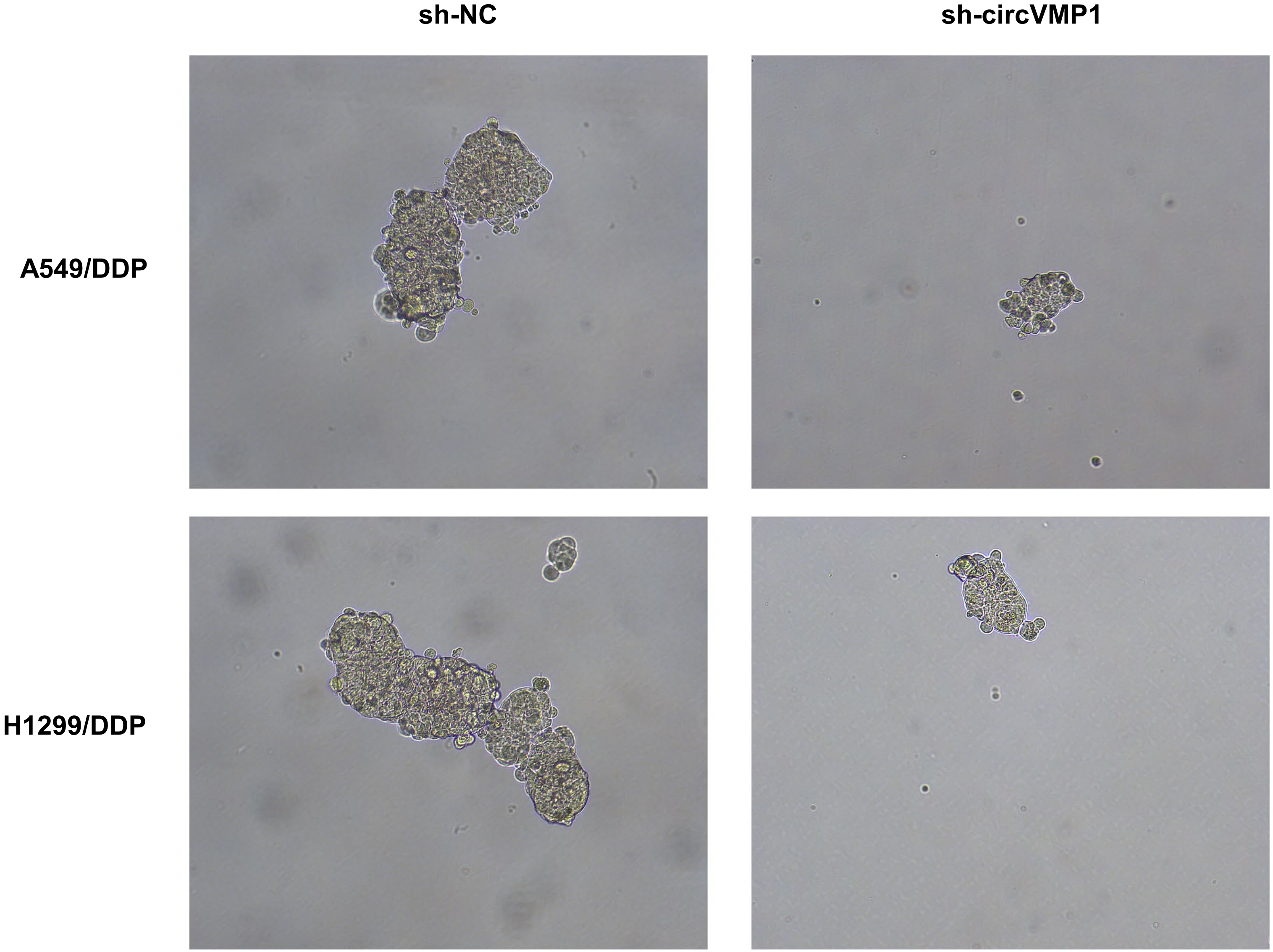

Fig2C

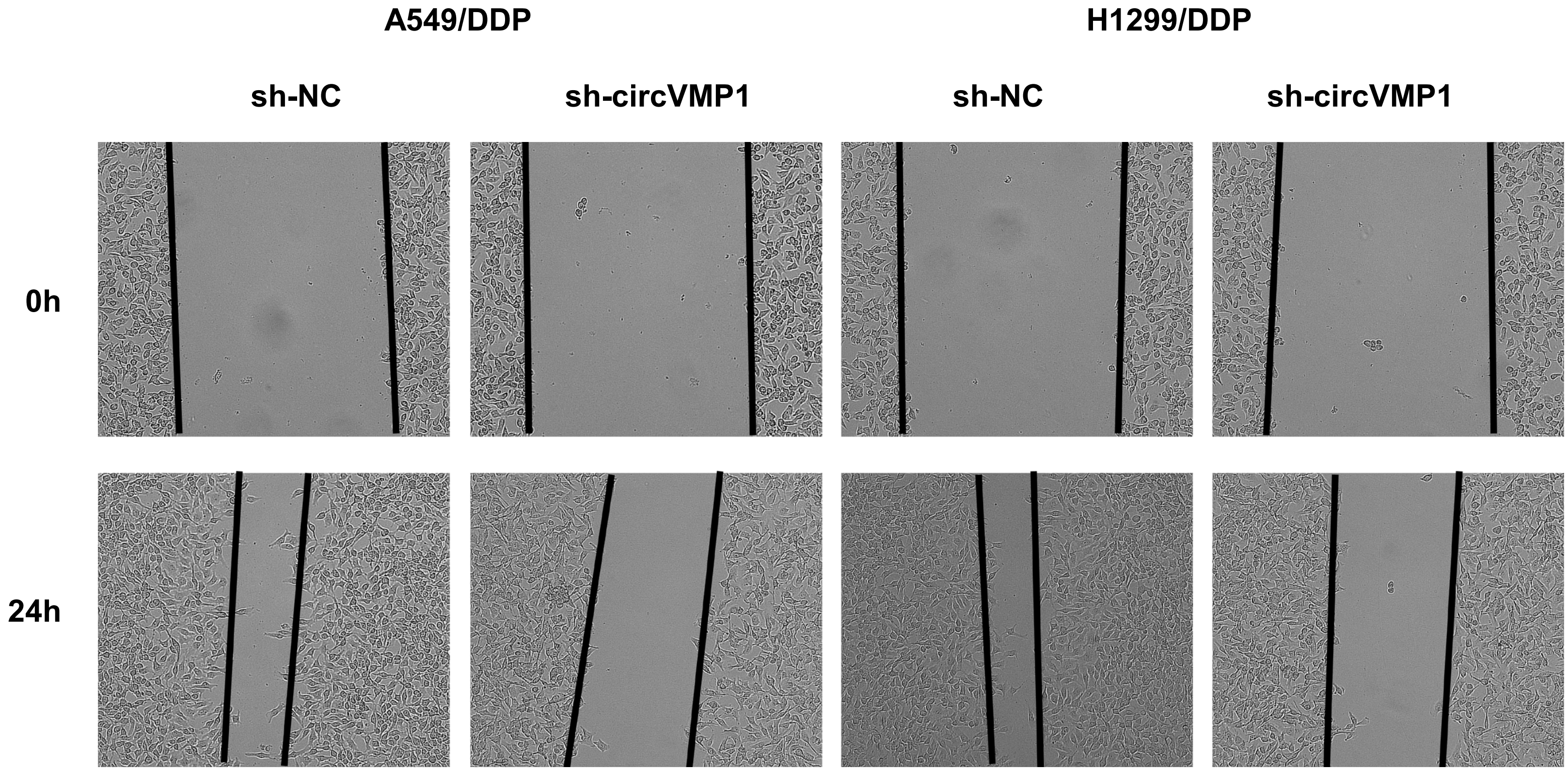

Fig2D

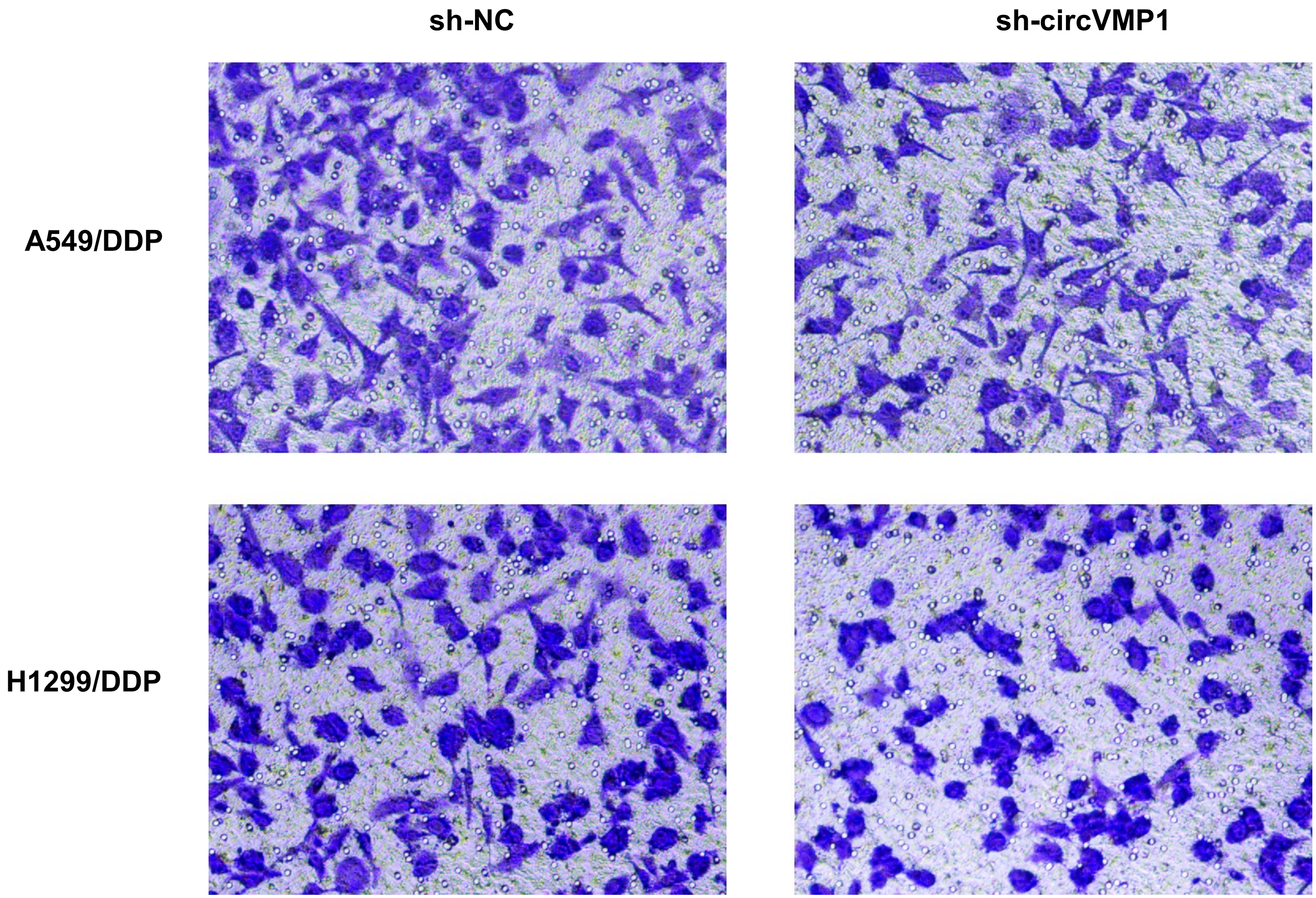

Fig2E

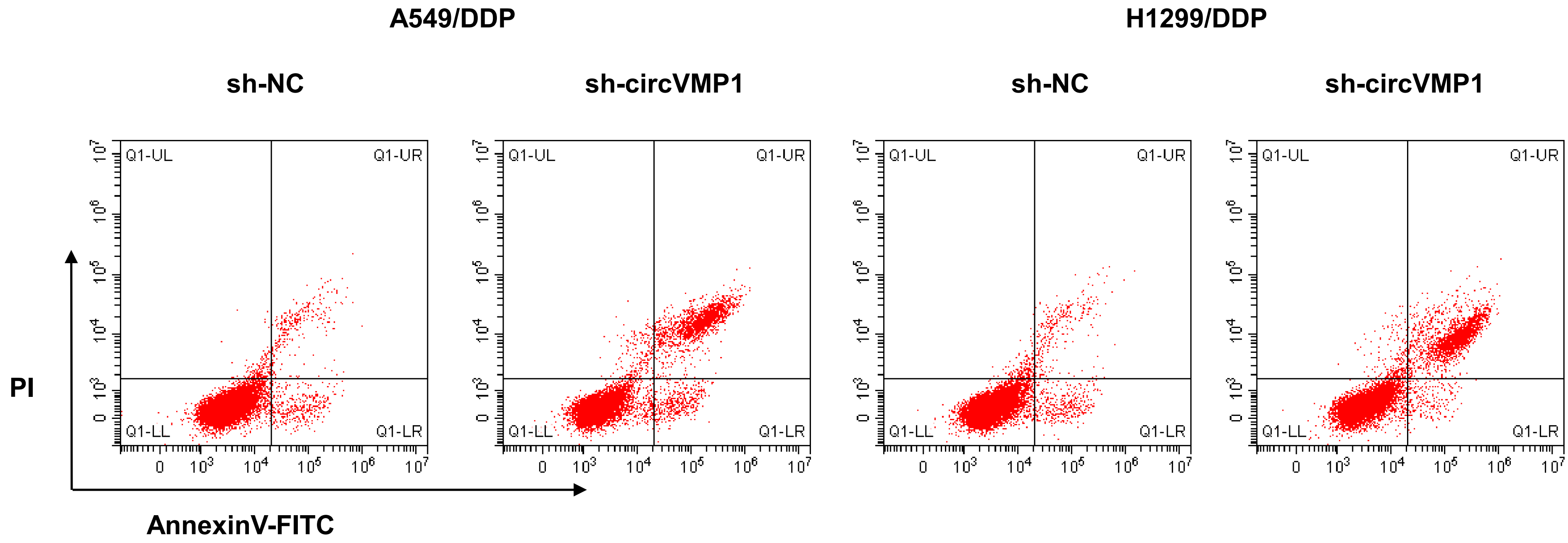

A549/DDP
H1299/DDP
sh-NC
sh-circVMP1
sh-NC
sh-circVMP1
Q1-UL
Q1-UR
Q1-LL
Q1-LR
PI
AnnexinV-FITC

Fig5A

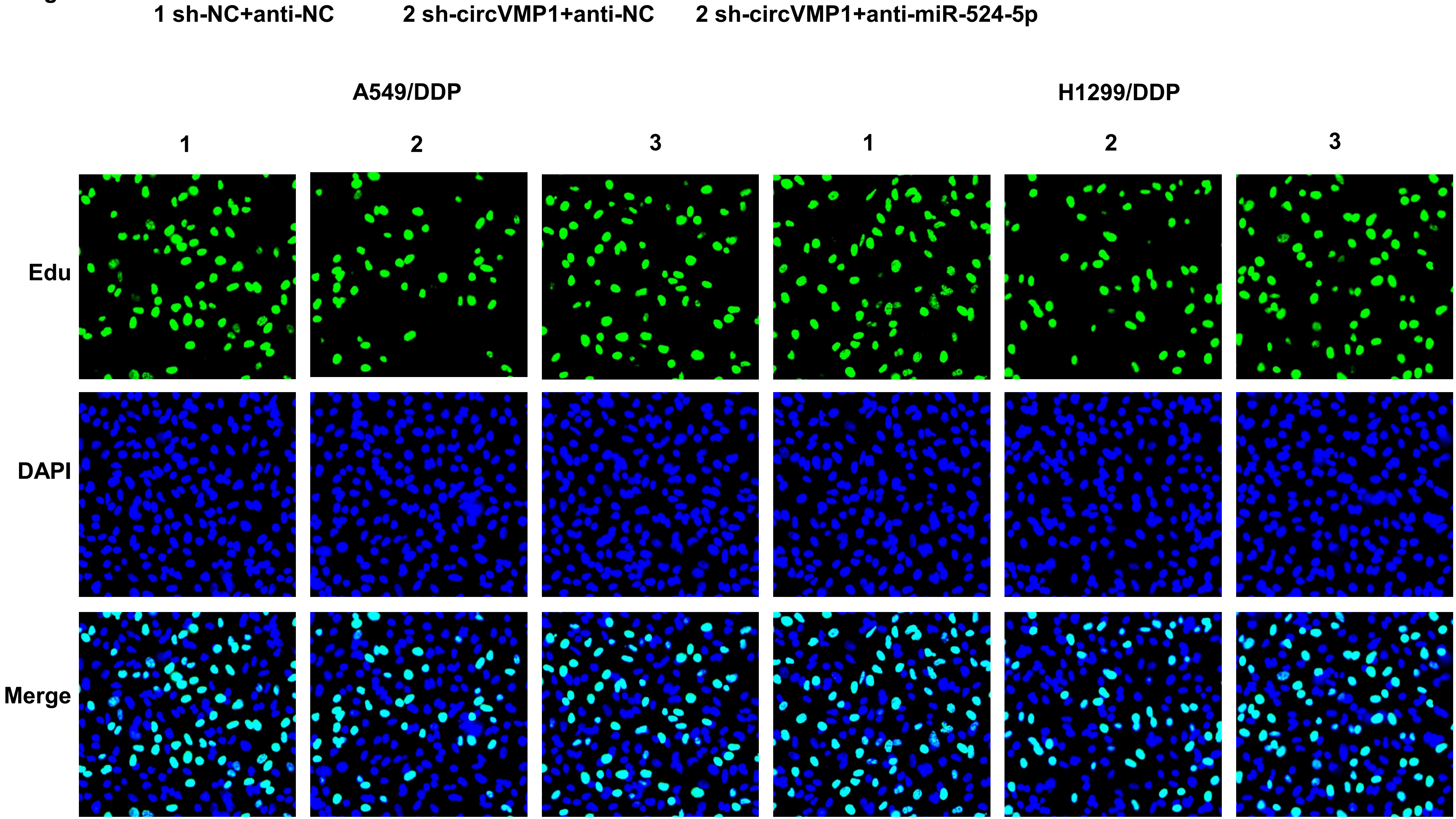

1 sh-NC+anti-NC
2 sh-circVMP1+anti-NC
2 sh-circVMP1+anti-miR-524-5p
A549/DDP
H1299/DDP
1
2
3
1
2
3
Edu
DAPI
Merge

Fig5B

1 sh-NC+anti-NC   2 sh-circVMP1+anti-NC   2 sh-circVMP1+anti-miR-524-5p

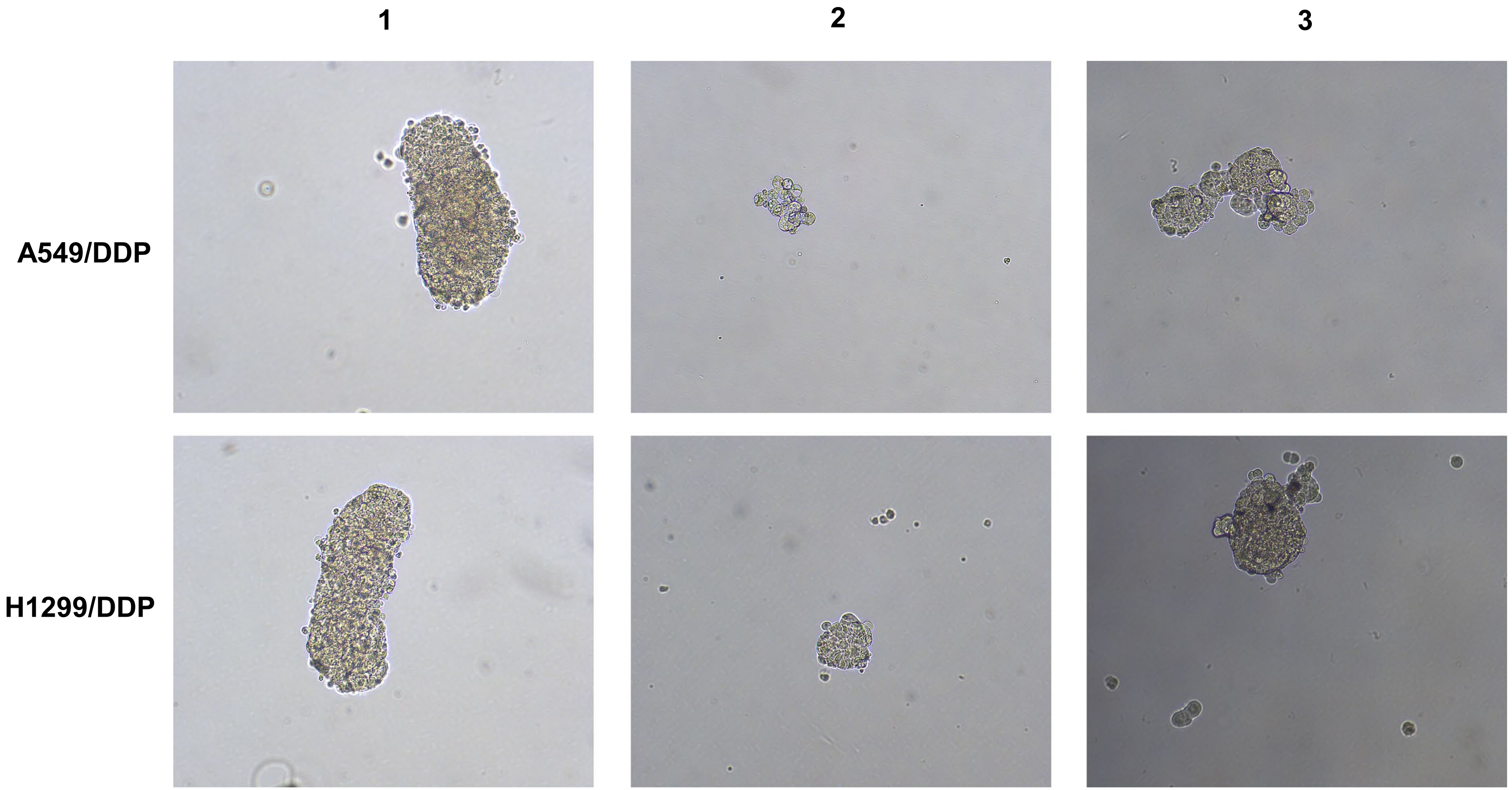

Fig5C

1 sh-NC+anti-NC 2 sh-circVMP1+anti-NC 2 sh-circVMP1+anti-miR-524-5p

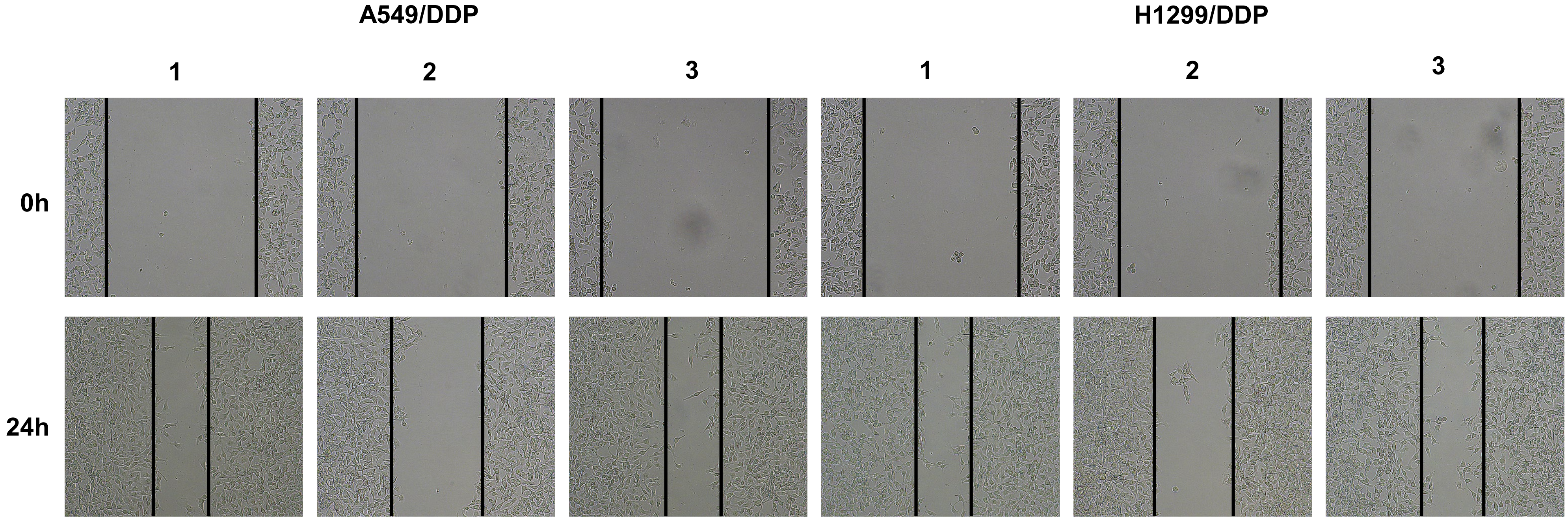

Fig5D 1 sh-NC+anti-NC 2 sh-circVMP1+anti-NC 2 sh-circVMP1+anti-miR-524-5p

A549/DDP H1299/DDP

1 2 3 1 2 3

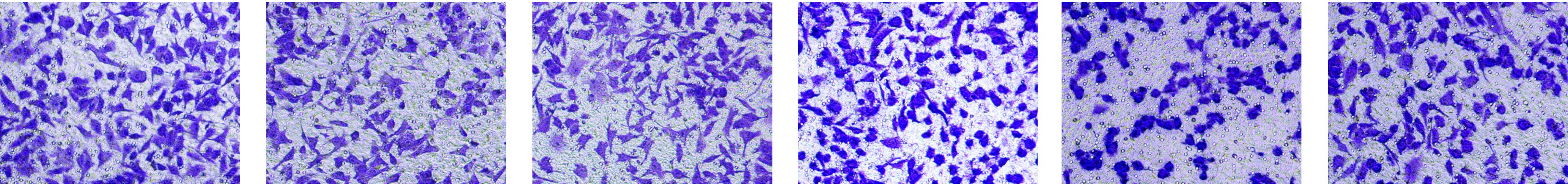

Fig5E 1 sh-NC+anti-NC 2 sh-circVMP1+anti-NC
2 sh-circVMP1+anti-miR-524-5p

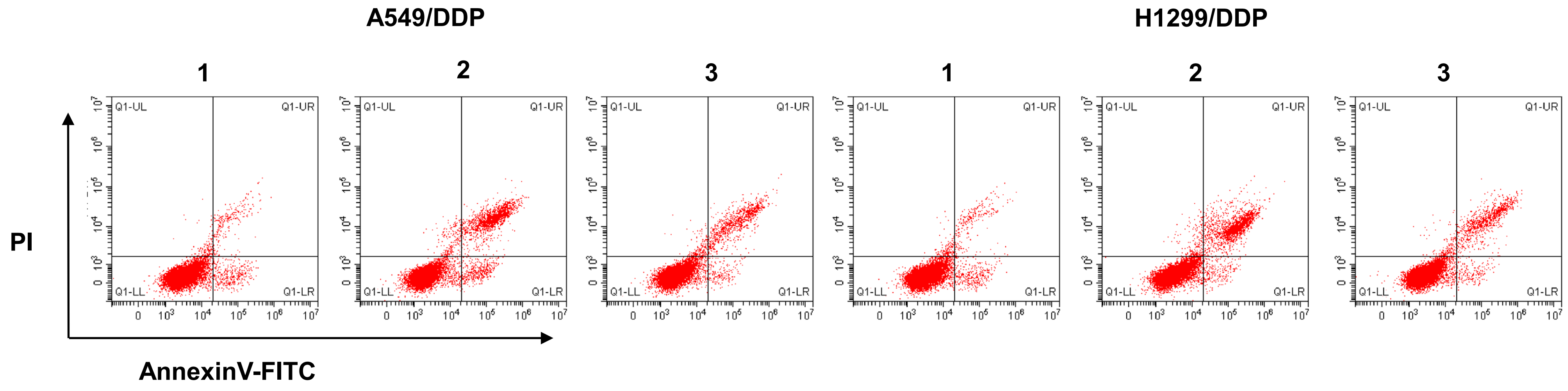

Fig6A

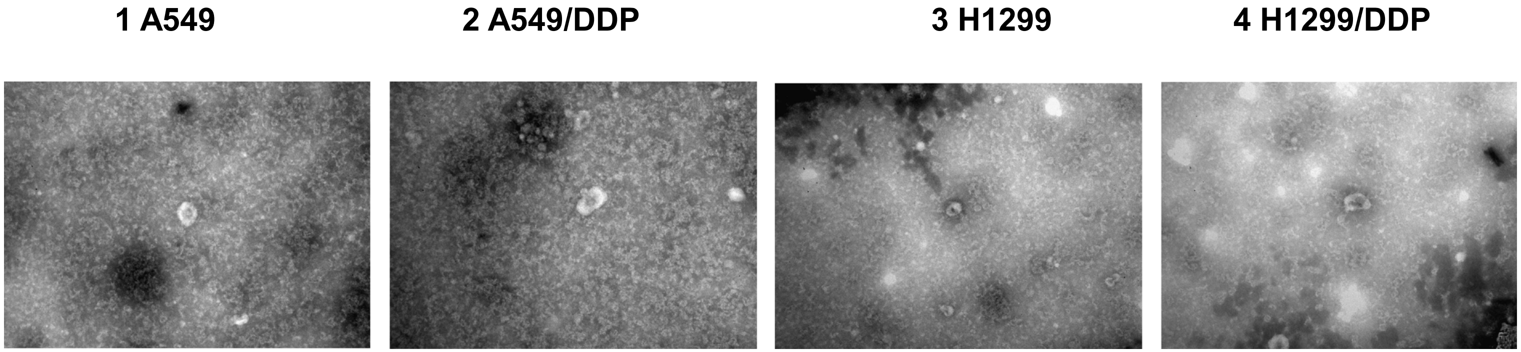

Fig7E

1 PBS 2 A549/DDP-sh-NC exo 3 A549/DDP-sh-circVMP1 exo

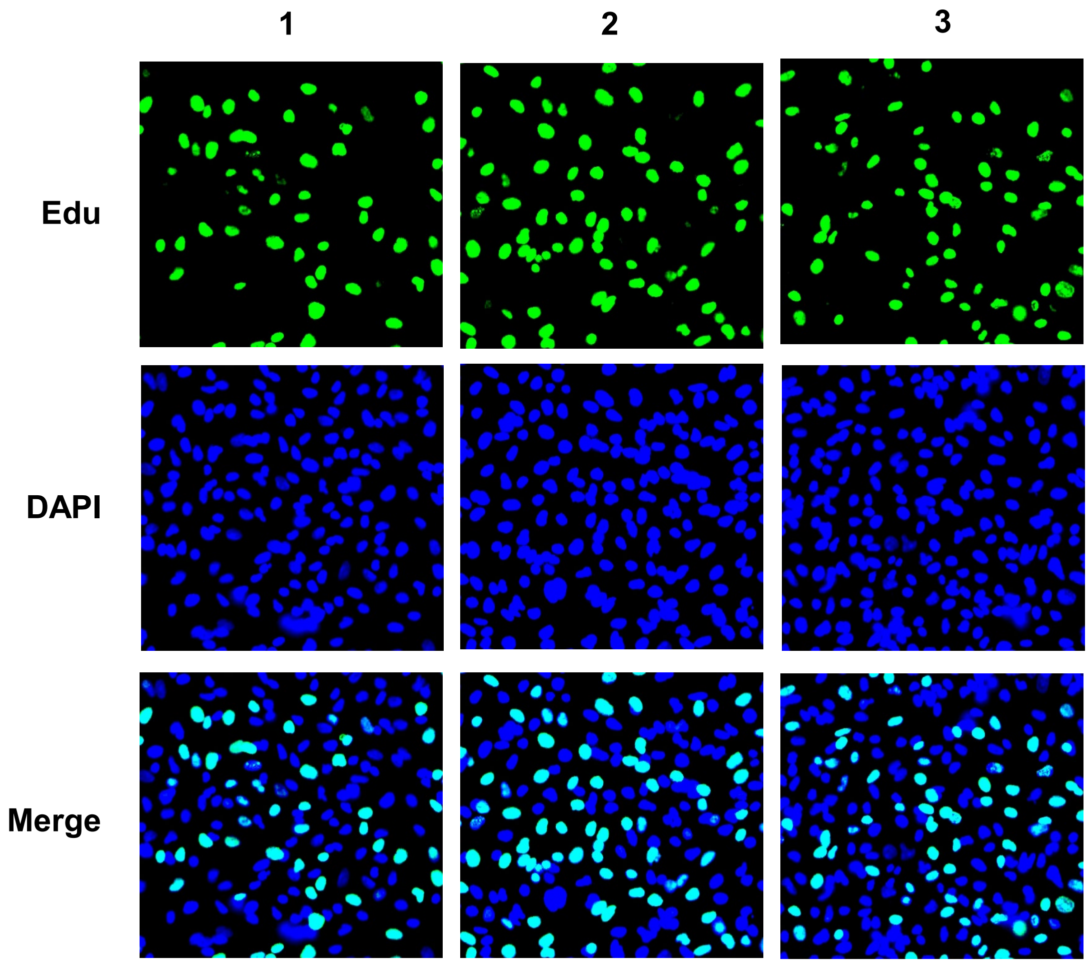

Fig7F

1 PBS 2 H1299/DDP-sh-NC exo 3 H1299/DDP-sh-circVMP1 exo

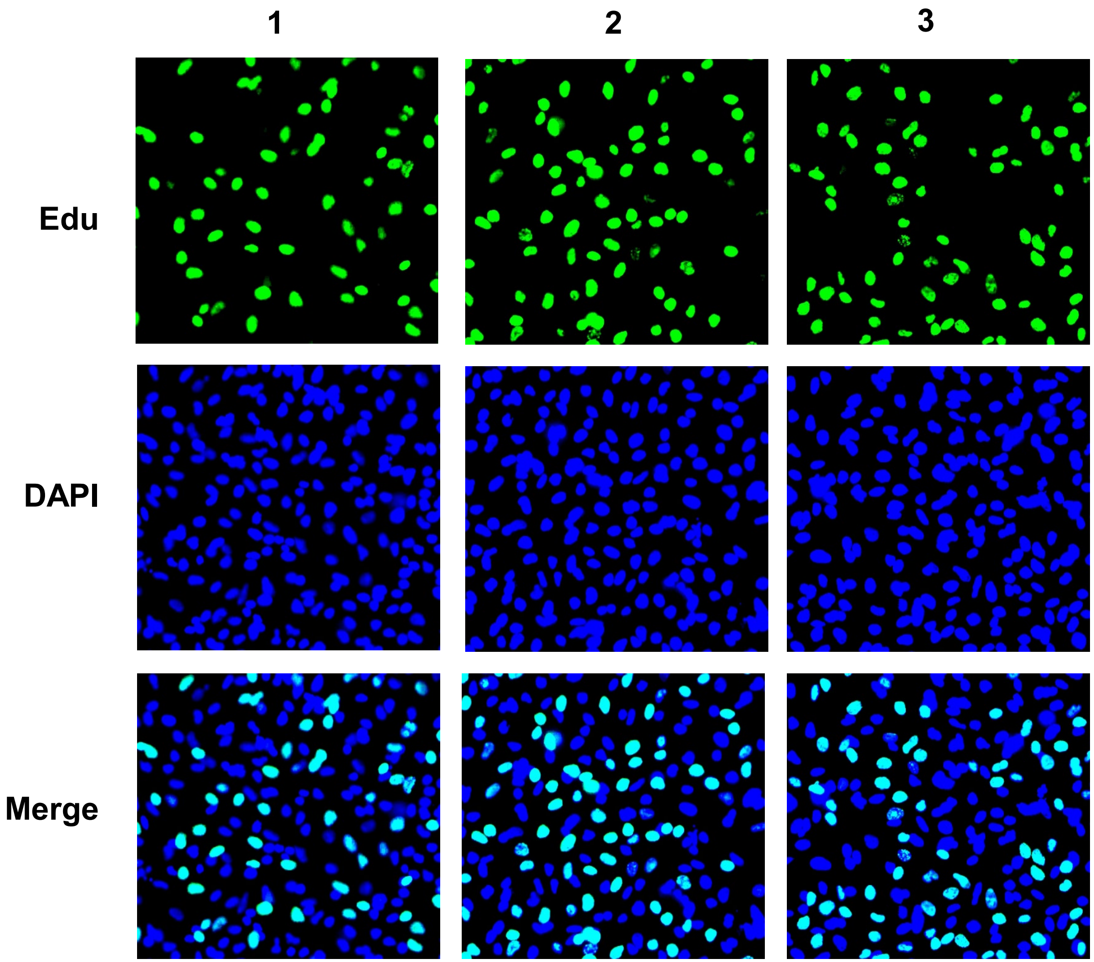

Fig7G

1 PBS 2 A549/DDP-sh-NC exo 3 A549/DDP-sh-circVMP1 exo

1 2 3

A549

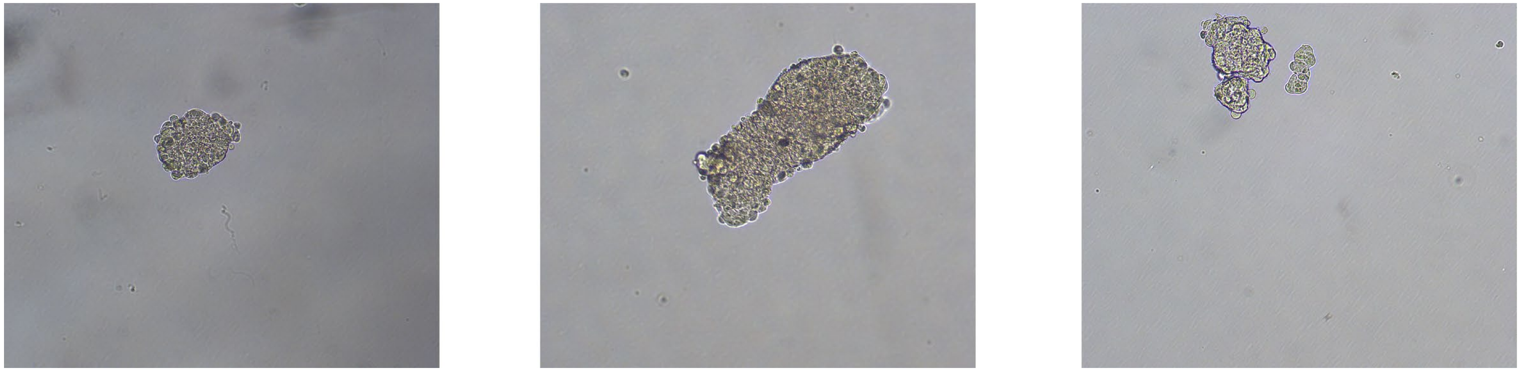

1 PBS 2 H1299/DDP-sh-NC exo 3 H1299/DDP-sh-circVMP1 exo

1 2 3

H1299

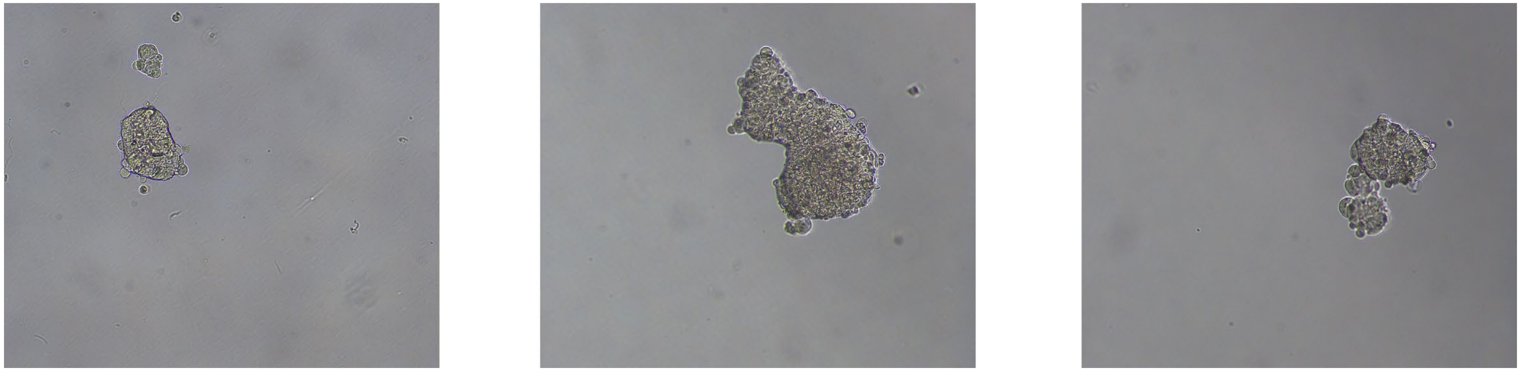

Fig7J

1 PBS 2 A549/DDP-sh-NC exo 3 A549/DDP-sh-circVMP1 exo

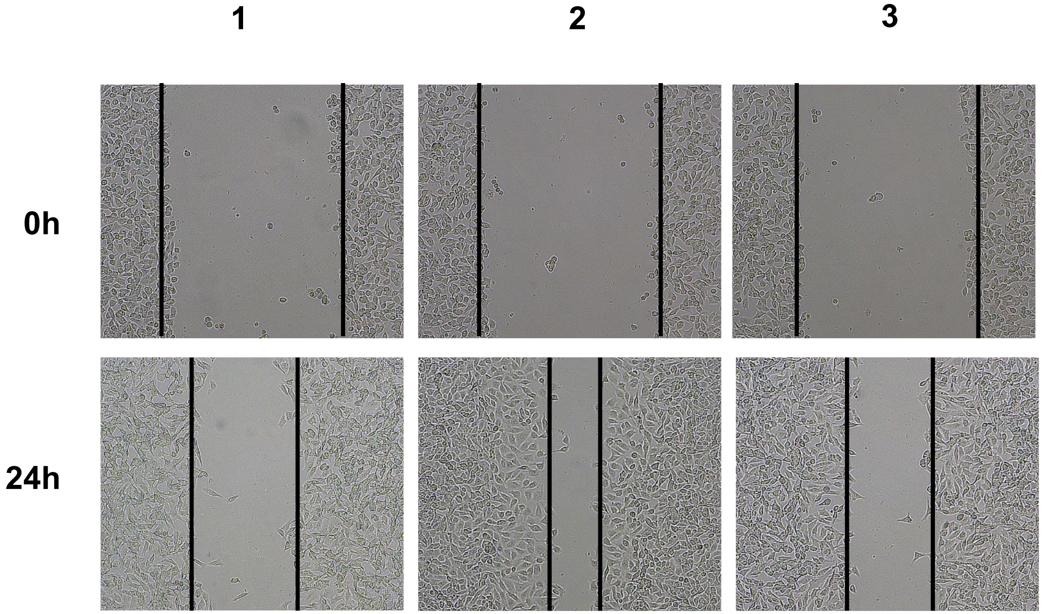

Fig7K

1 PBS 2 H1299/DDP-sh-NC exo 3 H1299/DDP-sh-circVMP1 exo

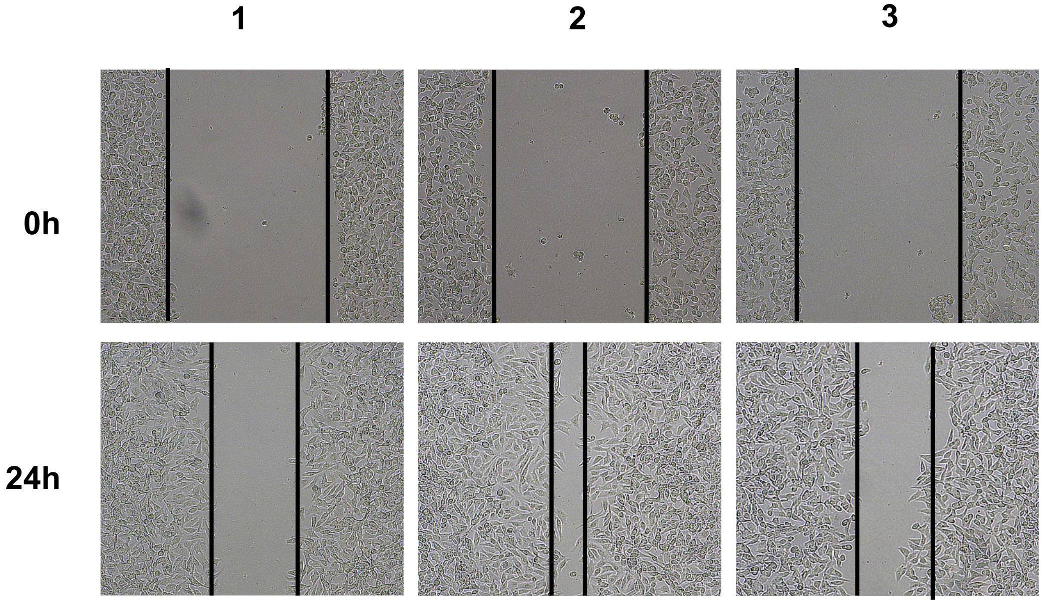

Fig7L

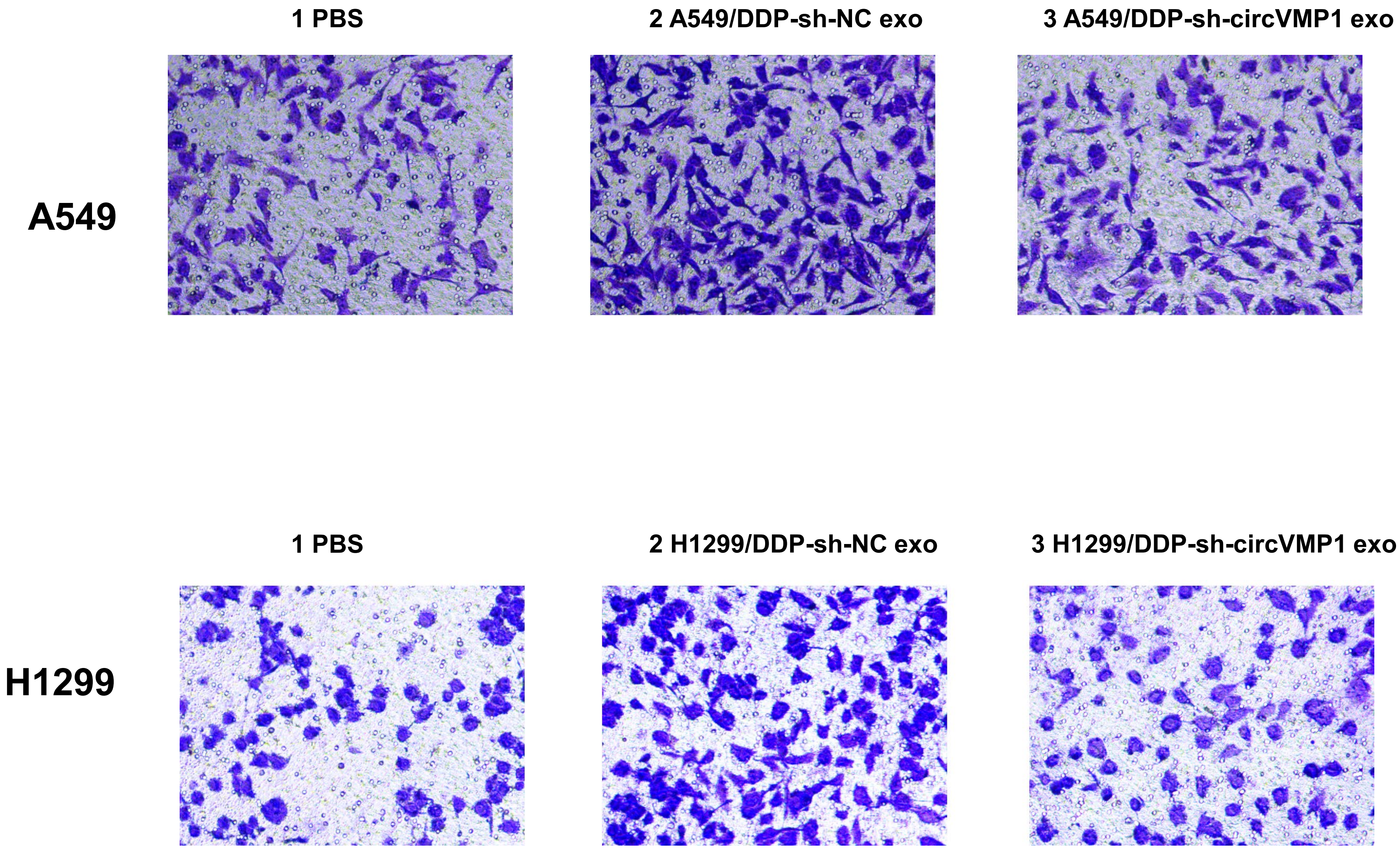

1 PBS
2 A549/DDP-sh-NC exo
3 A549/DDP-sh-circVMP1 exo
A549
1 PBS
2 H1299/DDP-sh-NC exo
3 H1299/DDP-sh-circVMP1 exo
H1299

Fig7O

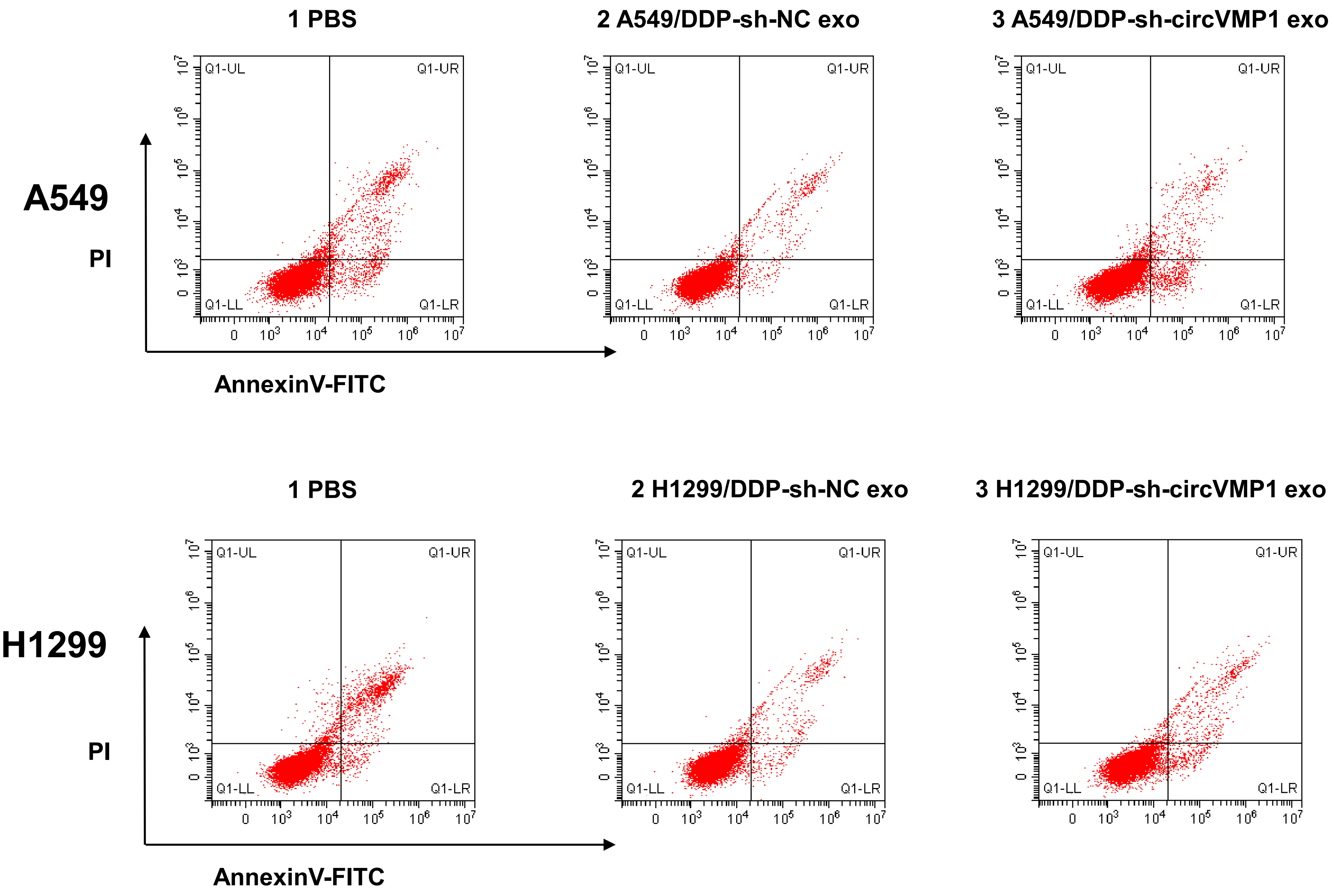

1 PBS
2 A549/DDP-sh-NC exo
3 A549/DDP-sh-circVMP1 exo
A549
PI
AnnexinV-FITC
1 PBS
2 H1299/DDP-sh-NC exo
3 H1299/DDP-sh-circVMP1 exo
H1299
PI
AnnexinV-FITC
Q1-UL
Q1-UR
Q1-LL
Q1-LR

Fig8A

1 PBS **2 A549/DDP-sh-NC exo** **3 A549/DDP-sh-circVMP1 exo**

1

2

3

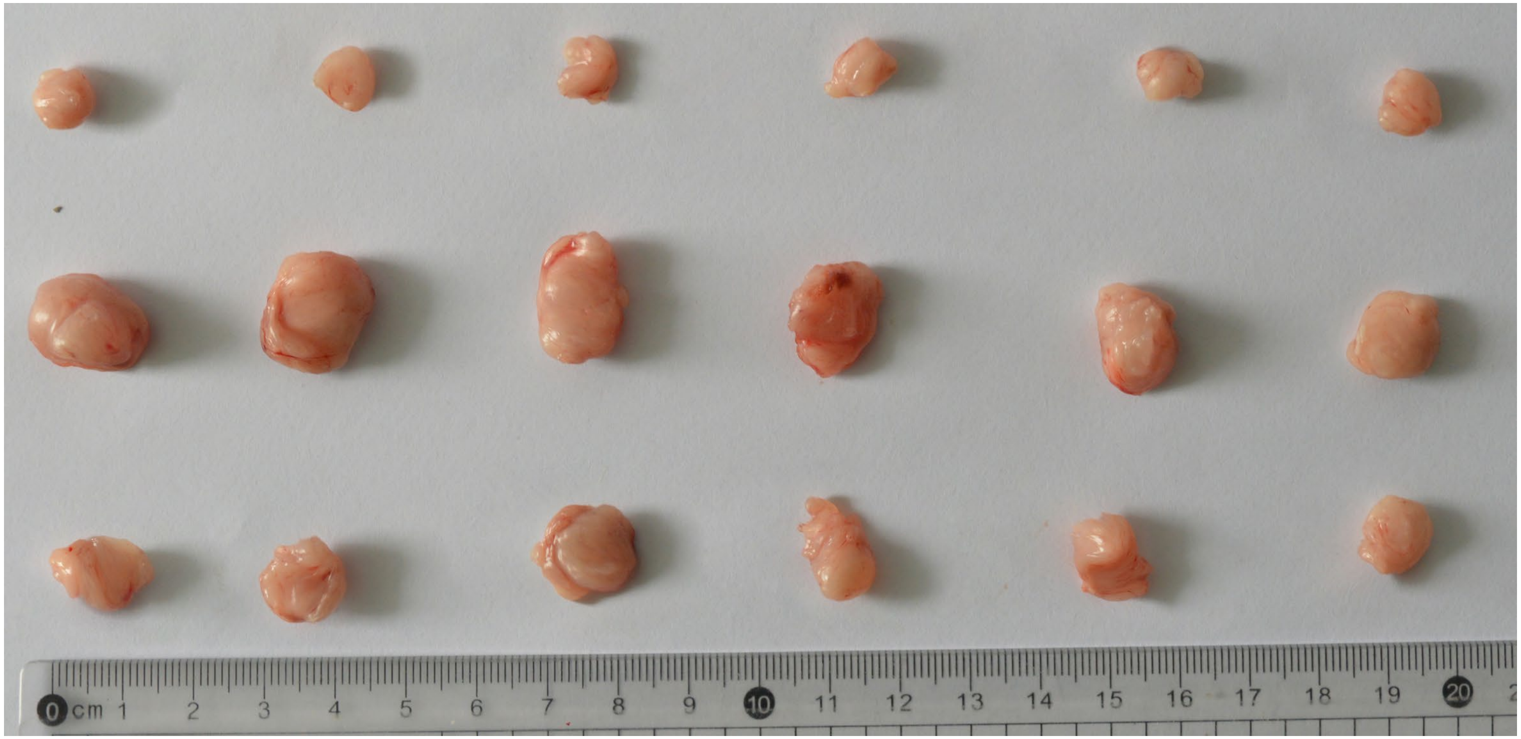

Fig8D

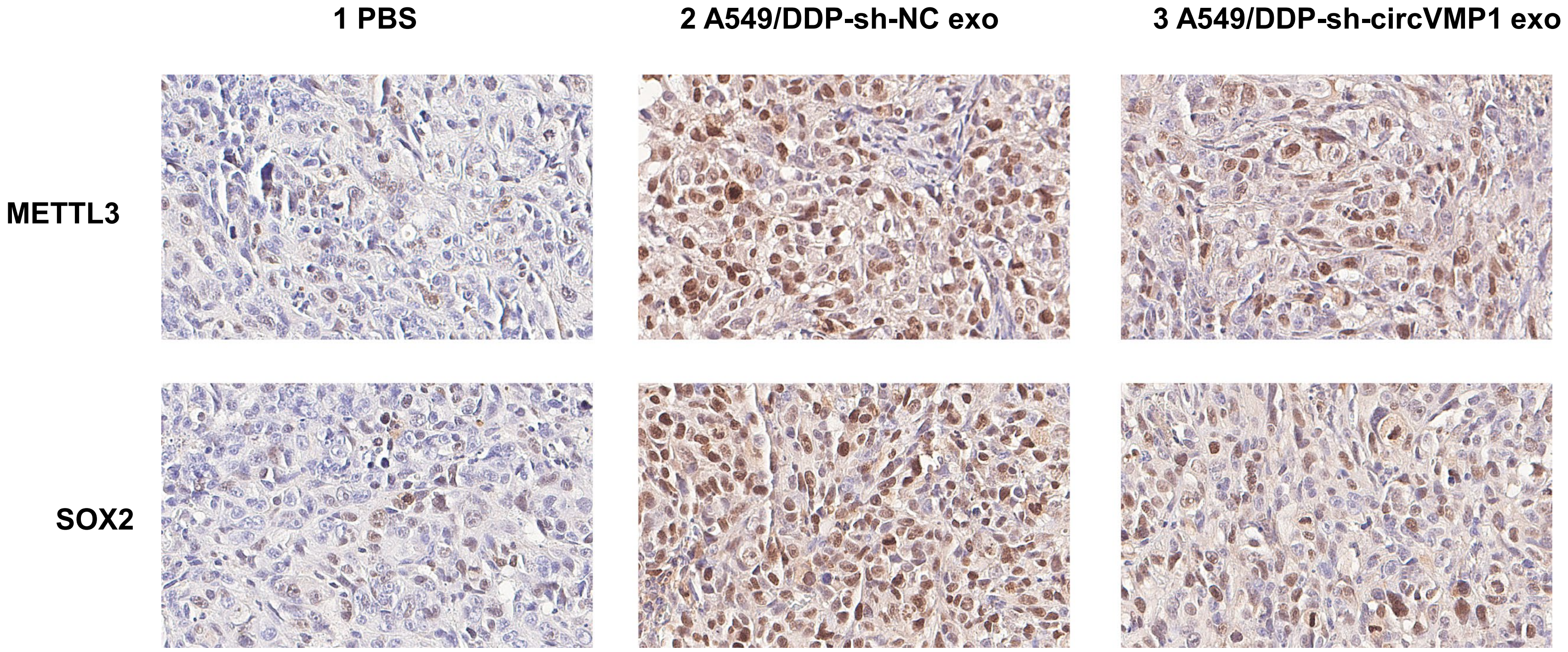
1 PBS
2 A549/DDP-sh-NC exo
3 A549/DDP-sh-circVMP1 exo
METTL3
SOX2

Fig9A

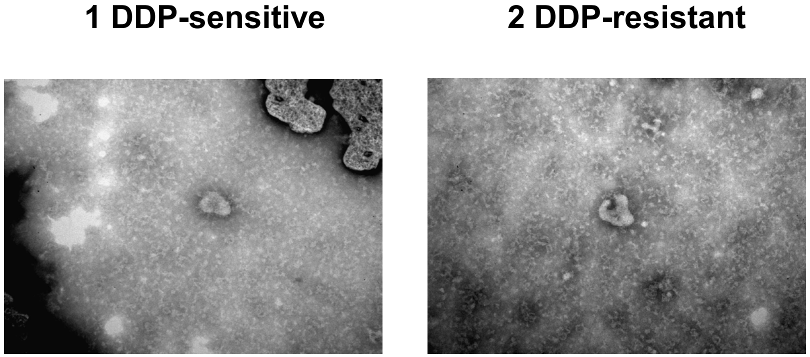

Supplement: Supplemental Material [file IDRD_A_2057617_SM7599.pdf]
